# Smoothing and extraction of traits in the growth analysis of noninvasive phenotypic data

**DOI:** 10.1186/s13007-020-00577-6

**Published:** 2020-03-10

**Authors:** Chris Brien, Nathaniel Jewell, Stephanie J. Watts-Williams, Trevor Garnett, Bettina Berger

**Affiliations:** 1 The Plant Accelerator, Australian Plant Phenomics Facility, The University of Adelaide, PMB 1, Glen Osmond, SA 5064 Australia; 2 School of Agriculture, Food and Wine, The University of Adelaide, PMB 1, Glen Osmond, SA 5064 Australia; 3 School of Information Technology and Information Sciences, University of South Australia, GPO Box 2471, Adelaide, SA 5001 Australia

**Keywords:** High-throughput phenotyping, Growth analysis, Growth traits, Functional analysis, Greenhouse experiments, Longitudinal analysis, Tomato, Random regression modelling

## Abstract

**Background:**

Non-destructive high-throughput plant phenotyping is becoming increasingly used and various methods for growth analysis have been proposed. Traditional longitudinal or repeated measures analyses that model growth using statistical models are common. However, often the variation in the data is inappropriately modelled, in part because the required models are complicated and difficult to fit. We provide a novel, computationally efficient technique that is based on smoothing and extraction of traits (SET), which we compare with the alternative traditional longitudinal analysis methods.

**Results:**

The SET-based and longitudinal analyses were applied to a tomato experiment to investigate the effects on plant growth of zinc (Zn) addition and growing plants in soil inoculated with arbuscular mycorrhizal fungi (AMF). Conclusions from the SET-based and longitudinal analyses are similar, although the former analysis results in more significant differences. They showed that added Zn had little effect on plants grown in inoculated soils, but that growth depended on the amount of added Zn for plants grown in uninoculated soils. The longitudinal analysis of the unsmoothed data fitted a mixed model that involved both fixed and random regression modelling with splines, as well as allowing for unequal variances and autocorrelation between time points.

**Conclusions:**

A SET-based analysis can be used in any situation in which a traditional longitudinal analysis might be applied, especially when there are many observed time points. Two reasons for deploying the SET-based method are (i) biologically relevant growth parameters are required that parsimoniously describe growth, usually focussing on a small number of intervals, and/or (ii) a computationally efficient method is required for which a valid analysis is easier to achieve, while still capturing the essential features of the exhibited growth dynamics. Also discussed are the statistical models that need to be considered for traditional longitudinal analyses and it is demonstrated that the oft-omitted unequal variances and autocorrelation may be required for a valid longitudinal analysis. With respect to the separate issue of the subjective choice of mathematical growth functions or splines to characterize growth, it is recommended that, for both SET-based and longitudinal analyses, an evidence-based procedure is adopted.

## Background

High-throughput phenotyping (HTP) has become an important tool in investigating shoot growth and structure in a range of plants that include rice, maize, sorghum, wheat, barley, chickpeas, setaria, medic, strawberries and tomatoes, either for studying shoot growth responses per se or as a precursor to genetic analysis [1–13]. Because it involves non-invasive phenotyping of the same plants at different time points, it is now possible to measure many plants at many time points so that the precision of growth analyses [14] is much improved. The basic structure of such data is that there are units, or ‘subjects’, on each of which measurements are made over time.

One approach to analyzing growth is to carry out a functional analysis in which a mathematical function, anticipated to be able to follow the growth pattern, is fitted. Fundamental to such analyses is the choice of function. It has been common to fit exponential, logistic and other mathematical functions of various forms to describe growth [15]. As noted in Hunt [14] and Shipley and Hunt [16], the problem with using specific functions is that the growth may not fit the assumed form and this led these authors and others [2, 5, 17–20] to recommend the use of splines to model growth. Shipley and Hunt [16] highlighted that it is often not possible to see deviations from the assumed functional form by examining a plot of the growth over time. Genetic markers have been successfully detected using semiparametric smoothing of the data [2, 5]. On the other hand, one of the attractions of using mathematical functions is that the parameters associated with them often have biological interpretations.

Here we outline and describe our experiences with two techniques for characterizing the dynamics of growth using data from HTP facilities. For both of these techniques, growth might be characterized by fitting either mathematical growth functions or splines to remove the transient deviations that occur in such data. The first technique is a computationally efficient method that we have developed and is called smoothing and extracted trait (SET) analysis. Essentially, the data for each individual is first smoothed and this is followed by the extraction of traits that are to be statistically analyzed. The second will be referred to as longitudinal analysis, also known as repeated measurements or growth curve analysis. Here, it employs fixed and random regression models (FRM and RRM) that are based on natural cubic smoothing splines (FRMS and RRMS) [21, 22]. The difference between FRM(S) and RRM(S) is that the intercepts and slopes are fixed for FRM(S) and, except for an overall intercept and slope, are random for RRM(S). Further, the statistical analysis will allow for unequal variance and autocorrelation between time points. For both techniques, the issue of fitting a valid model is canvassed.

To illustrate the two techniques, the data from a tomato (*Solanum lycopersicum*) experiment [11] is used. This experiment involved the eight combinations of four levels of zinc (Zn) addition (0, 10, 40, and 90 mg Zn kg^−1^ soil) and either the addition of an arbuscular mycorrhizal fungal (AMF) inoculum (+AMF) or of a mock inoculum (−AMF) to the soil in pots with a single plant. The experiment consisted of 32 potted plants that were placed in carts on the conveyor system within a Smarthouse (Australian Plant Phenomics Facility, University of Adelaide), where they were imaged daily from 17 to 51 days after planting (DAP). While the previously reported results [11] cover only 27–43 DAP, the full data set was processed and is the subject of the analyses reported here. ‘Cart’ will be used as the generic term for the unit in this experiment, each physical cart holding a pot with a single plant.

## Results

**Fig. 1:**
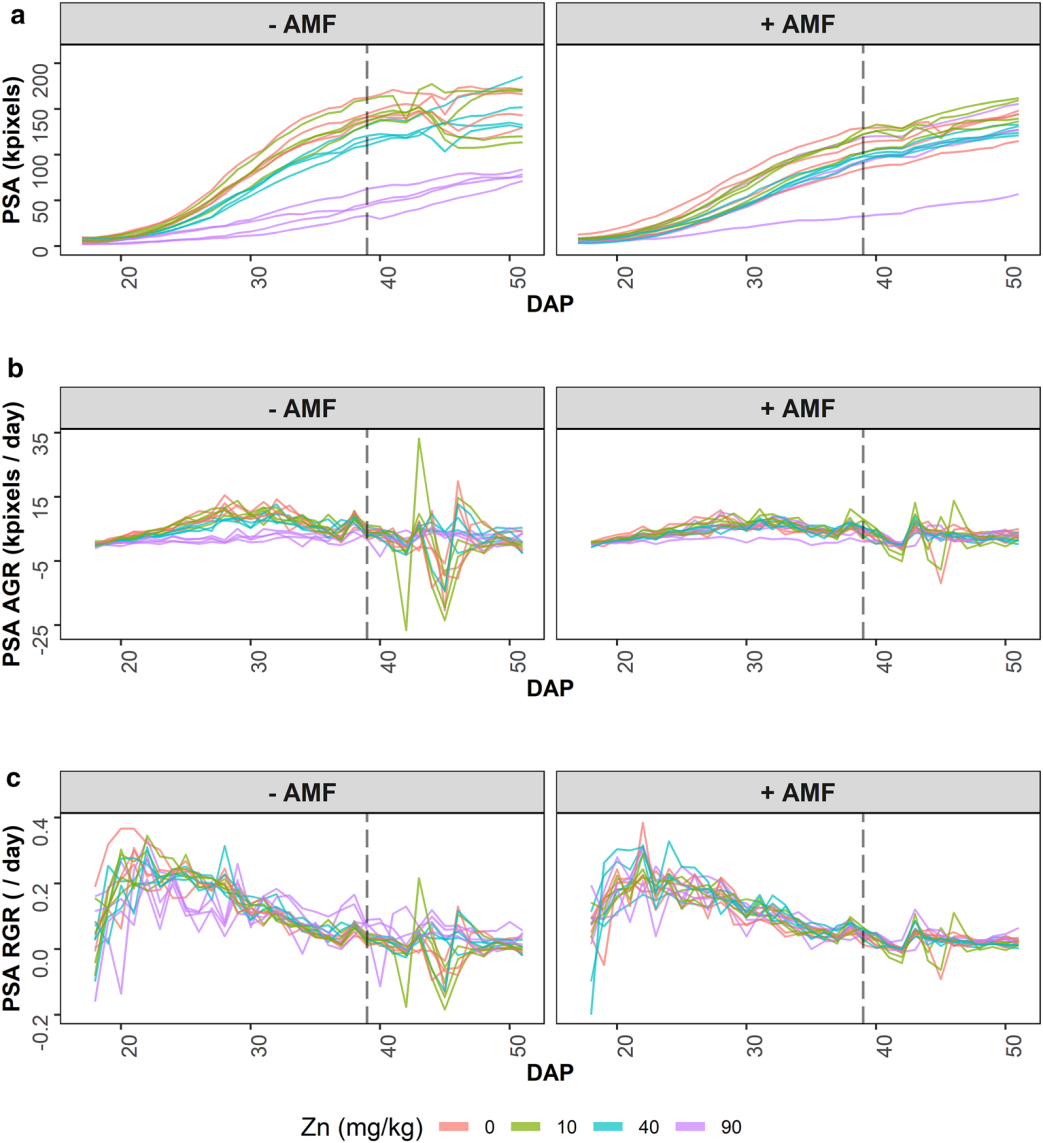
Profile plots for unsmoothed projected shoot area (PSA) and growth rates. The PSA (**a**) is shown over DAP 17–51, as are the continuous absolute growth rate (AGR) (**b**) and relative growth rate (RGR) (**c**) calculated from the PSA. For each trait, AMF treatments occupy separate panes. Each line corresponds to one of the 32 carts, each with a potted plant. The dashed, vertical black line indicates the start of a 3-day interruption to watering

The raw data obtained from the image processing is exhibited in the profile plots in Fig. 1. In addition to PSA, the continuous PSA absolute and relative growth rates (PSA AGR and PSA RGR) are shown, these being calculated by differencing consecutive PSA and ln(PSA) values, respectively. There is a marked “sawtooth” pattern evident in the PSA AGR and PSA RGR, this pattern not being evident in the PSA plot. The results of analyzing the PSA by a SET-based and a longitudinal analysis of the tomato data are now described.

### A SET-based analysis of the tomato data

**Fig. 2:**
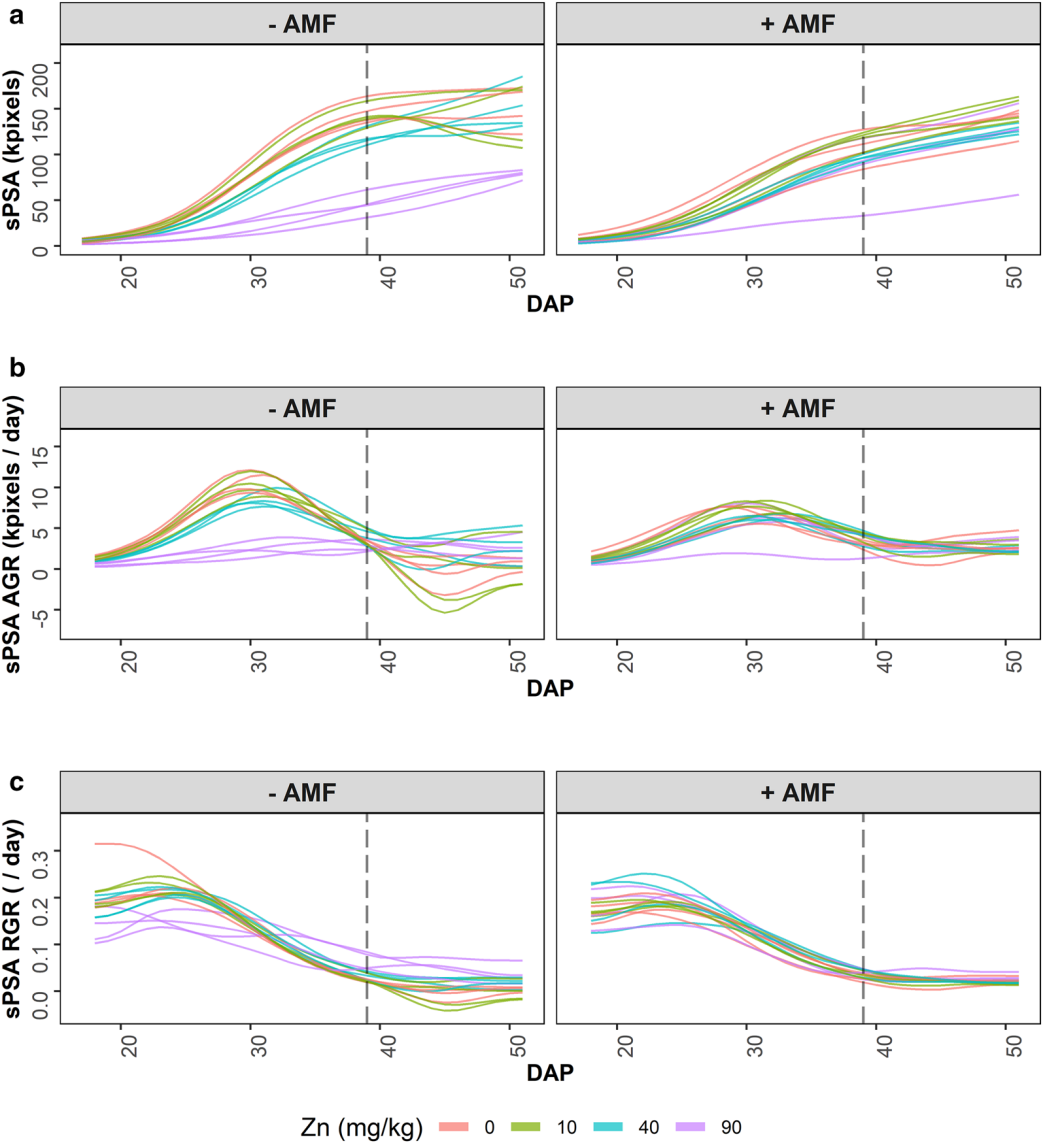
Profile plots for the smoothed projected shoot area (sPSA) and growth rates. The sPSA (**a**) is shown over DAP 17–51, as are the continuous absolute growth rate (AGR) (**b**) and relative growth rate (RGR) (**c**) calculated from the sPSA. For each trait, AMF treatments occupy separate panes. The dashed, vertical black line indicates the start of a 3-day interruption to watering

For this analysis the PSA is first smoothed. We investigated direct- and log-smoothing of the PSA using natural cubic splines for several values of the smoothing degrees of freedom (DF), as well as the fitting of a three-parameter logistic curve. Log-smoothing with six DF was chosen to yield the smoothed projected shoot area (sPSA). The continuous sPSA AGR and sPSA RGR are calculated by differencing consecutive sPSA and ln(sPSA) values. Figure 2 presents plots of these three responses. Using these, time intervals with end points at DAPs 18, 22, 27, 33, 39, 43 and 51 were chosen. For the analysis, the sPSA values for each of these end points were extracted and the mean sPSA AGR and sPSA RGR calculated for each of the intervals DAP 18–22, 22–27, 27–33, 33–39, 39–43, 43–51. Thus there are seven single-DAP traits extracted for sPSA and six interval traits extracted for sPSA AGR and six for sPSA RGR.

Each of these 19 extracted growth traits was analyzed separately and a summary of the hypothesis tests carried out to determine the significance of the effects of Zn and AMF inoculation on these traits is given in Additional file 1: Table S1. They show that only Zn had a significant effect (*p* ≤ 0.05) on sPSA at DAPs 18 and 22; subsequent to these DAPs (viz. DAPs 27, 33, 39, 43 and 51), Zn and AMF interacted significantly (*p* ≤ 0.05) in their effect on sPSA. For sPSA AGR, there were significant (*p* ≤ 0.05) interactions of Zn and AMF for DAP intervals 22–27, 27–33 and 33–39, while for sPSA RGR there were significant (*p* ≤ 0.05) interactions for DAP intervals 18–22, 22–27, 39–43 and 43–51.

**Fig. 3:**
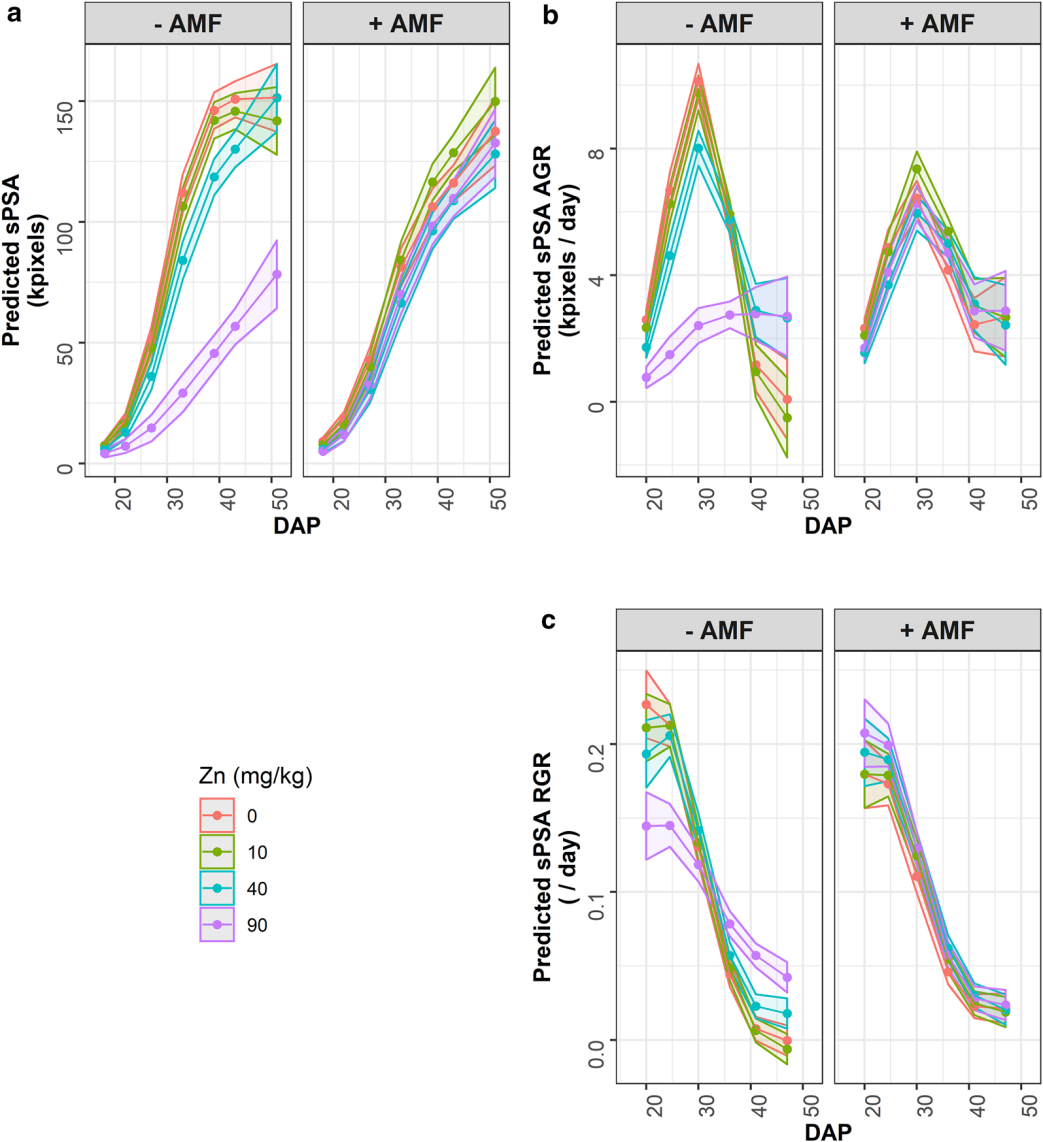
Results of the SET-based analyses, showing predicted sPSA (**a**), sPSA AGR (**b**) and sPSA RGR (**c**) for the chosen DAPs and time intervals. The error ribbons are the predicted values ± 0.5 LSD (*α*  =  0.05). Separated ribbons indicate a significant difference, in contrast to overlapping ribbons for which there is not a significant difference

To illustrate these effects, predicted values for each of the responses sPSA, sPSA AGR and sPSA RGR are combined and each presented in its own plot in Fig. 3. It shows that generally DAP 18–22 was a period of increasing AGR; DAP 22–27 was a period of even faster increases in the AGR; DAP 27–33 was a period during which the AGR peaked; DAP 33–39 was a period of reduced growth; DAP 39–43 is the period of restricted watering and followed by a 2-day recovery period when growth had slowed even more; DAP 43–51 was a period in which the AGR did not change or decreased further. The RGR, except for the first and last intervals, continued to decrease. There is little, if any, difference between the trends for the different Zn treatments over time for the plants grown in soils inoculated with AMF. For those grown with mock inoculation, the difference between the zero and 10 mg Zn kg^−1^ additions is small; the differences between these two concentrations and the other two concentrations varies over time for the sPSA, sPSA AGR and sPSA RGR. It can also be seen in Fig. 3 that the variance differs over time. However, each per-cart analysis involves a single time or time interval and the different variances between times are irrelevant to a single analysis.

The six interval traits for each of sPSA, sPSA AGR and sPSA RGR were jointly analyzed, as described in Additional file 2. The differences between the results of the joint analyses and those of the separate analyses just described are minor. However, the joint analyses were more difficult to perform than the separate analyses. It would appear that, as long as comparisons are limited to predictions at the same time, then separate analyses are valid.

### A longitudinal analysis of the tomato data

Each of PSA and ln(PSA) was subjected to a longitudinal analysis, the aim of which was to obtain estimates of the trend over time of the PSA, PSA AGR and PSA RGR for each combination of Zn and AMF. The analysis fitted a mixed model to describe the behaviour of the complete set of values for one of the traits, without any pre-smoothing of the data. The fitted mixed model employed an RRMS in that random nonlinear trends between individual carts were specified by fitting natural cubic smoothing splines with random intercepts and slopes, and a knot per observed DAP. Homogeneous splines were fitted so that the amount of smoothing was the same for all carts. The mixed model also allowed for the residual variance to differ between DAPs and for there to be first-order autoregressive correlation between DAPs. For both PSA and ln(PSA), the variances differed significantly between the DAPs (*p* < 0.001) and the autocorrelation was significant (*p* < 0.001), the estimated first-order autoregressive correlation parameter being 0.88 for both PSA and ln(PSA). An FRMS, for which ten equally spaced knots was specified, was chosen to describe the trend for each combination of Zn and AMF using heterogeneous splines, that is, splines for which the amount of smoothing was allowed to differ between the combinations. Analyses were conducted to investigate the simplification of the FFRMs and the results are summarized in Additional file 3: Tables S2 and S4. For the trends over time, the hypothesis tests for both PSA and ln(PSA) lead to the conclusion that the trends differed significantly between the combination of Zn and AMF. For PSA, heterogeneous splines for the different combinations of Zn and AMF were significant (*p* = 0.001) so that the amount of smoothing differed between the treatments. For ln(PSA), the three forms of heterogeneous splines were not significant (*p* > 0.05), but the homogeneous splines differed significantly between the combinations of Zn and AMF (*p* = 0.002); the amount of the smoothing was the same for all Zn-AMF combinations, but the fitted curves differed. For both responses, the intercept varied significantly between the Treatments or combinations of Zn and AMF (*p* < 0.001).

**Fig. 4:**
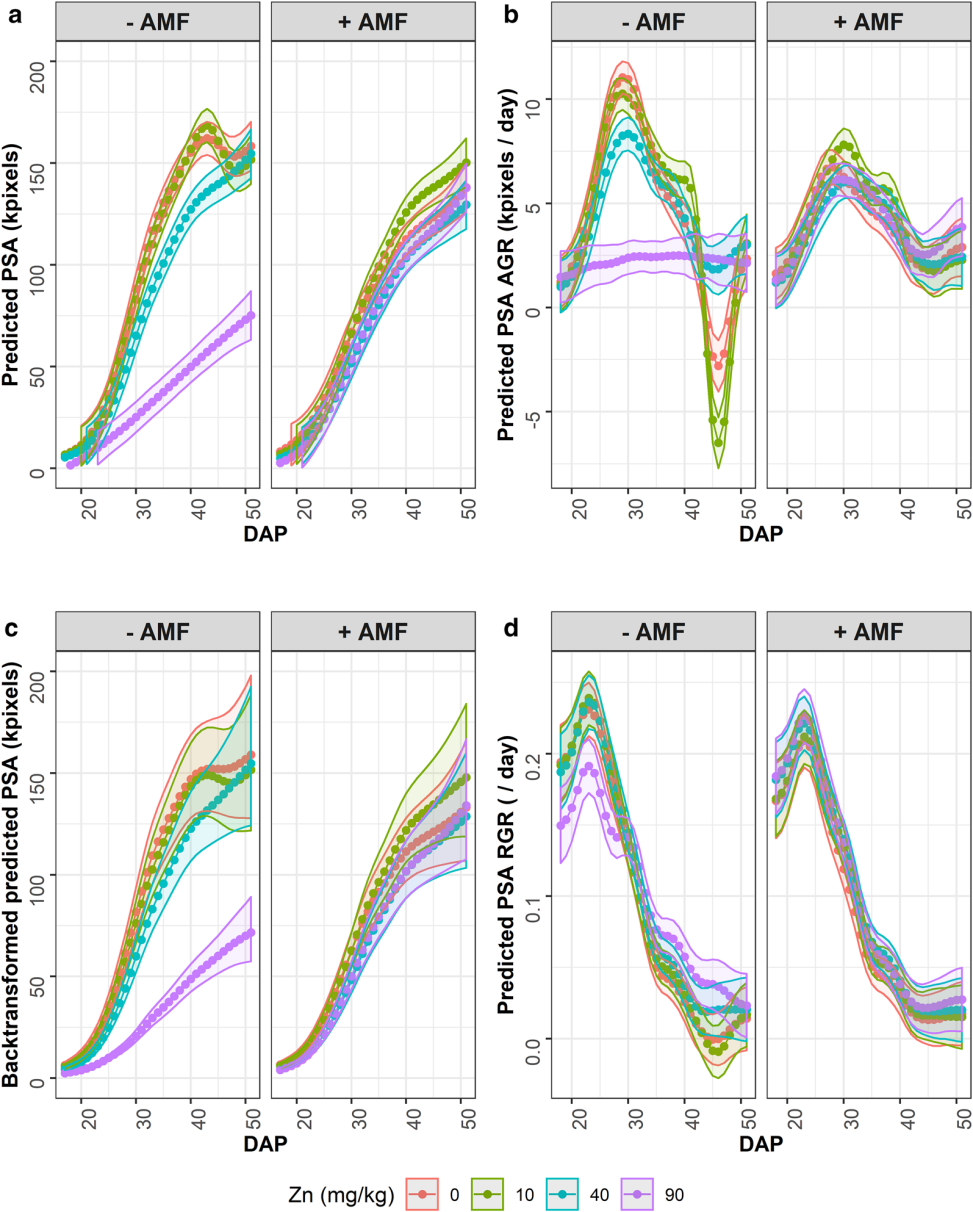
Results of the longitudinal analyses, showing predicted PSA (**a**), PSA AGR (**b**), backtransformed PSA (**c**), and PSA RGR (**d**) for smoothing with 10 knots. The error ribbons are the predicted values ± 0.5 LSD (*α*  =  0.05). Separated ribbons indicate a significant difference, in contrast to overlapping ribbons for which there is not a significant difference

The predicted values obtained from the analyses are exhibited in Fig. 4. It is noticeable that the predicted PSA and the backtransformed predicted PSA differ: the (Least Significant Difference) LSD increases as the DAP increases for the backtransformed predicted PSA, so that there are not the significant differences between the treatment with 40 mg kg^−1^ of added Zn and those with the lower levels of added Zn. There is some evidence in the residual plots in Additional file 3 of more outliers from the analysis of ln(PSA). Most differences in the predicted PSA trend occur for the −AMF treatment; also, the 90 mg kg^−1^ Zn treatment stands out from the other levels of Zn. These conclusions are similar to those reached from the SET-based analyses, although there are more significant differences for the SET-based analyses, especially for PSA RGR.

### Assuming residuals have equal variance and are uncorrelated for the longitudinal analysis

An analysis in which the significant unequal variances and autocorrelation of the residuals are removed from the fitted PSA model was performed to demonstrate the kind of effect that the reduced model can have on the predictions and their standard errors. Additional file 3: Figure S9, compares the predictions and their standard errors under the full and reduced models. The effect on the predictions is small, but there is a tendency for the standard errors to be somewhat larger under the reduced model as compared to those under the full model.

## Discussion

### Use descriptive plots to make evidence-based choices in deciding how to smooth growth data

An exploratory analysis is a crucial first step in analyzing data and is descriptive in nature; it does not involve any formal statistical modelling. For longitudinal data, it is often founded on profile plots that include a profile of a trait for each physical unit or ‘subject’ in the experiment by graphing the trait values for each unit over time.

Figure 1 gives the profile plots of the PSA, PSA AGR and PSA RGR for the tomato experiment. The “sawtooth” pattern evident in the PSA AGR and PSA RGR, but not the PSA, in Fig. 1 generally results from transient effects on the plants. They have been observed in all the experiments conducted in the Smarthouses at the Australian Plant Phenomics Facility, University of Adelaide. Secondly, it is clear that growth is not exponential because the RGR is decreasing, rather than constant. However, it may be logistic prior to DAP 39, when a 3-day, unintentional watering interruption occurred, because up to that point the AGR shows a single, symmetrical peak.

These profile plots are particularly useful because they provide an impression of the growth dynamics in the experiment, thus allowing an assessment of whether or not particular growth models may be appropriate for smoothing the data. The primary function of smoothing the PSA is to refine the description of the data by removing the transient effects evident in Fig. 1 in order to establish the underlying growth trajectories of the plants. Smoothing may be achieved by fitting (i) smoothing splines or (ii) a mathematical function, usually in the form of a nonlinear model. Smoothing with splines involve the subjective process of deciding on the method of smoothing and the smoothing DF; otherwise, if a mathematical function is to be used, the particular function has to be chosen and this involves a similarly subjective process.

**Fig. 5:**
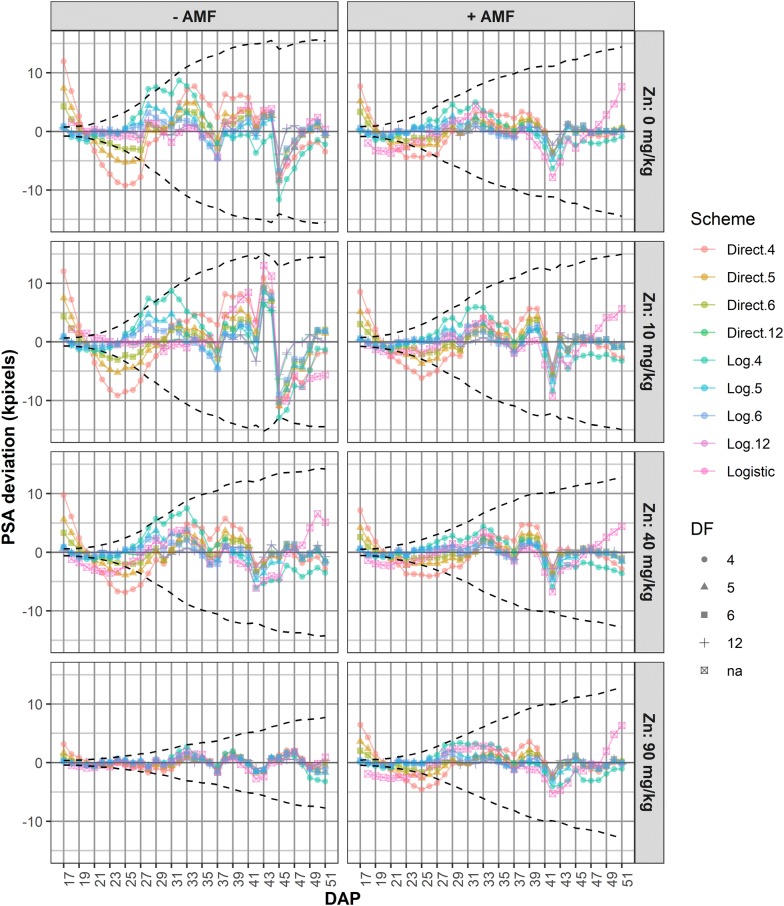
Median deviations of observed minus smoothed PSA values. Each line corresponds to a smoothing scheme, a scheme being the combination of a smoothing method with a value for the DF. Median values above zero indicate that the smoothed value is under-estimating the trend or that there is a positive transient effect; median values below zero indicate the opposite. The dashed black envelope is 10% of the log-smoothed sPSA for six DF

**Fig. 6:**
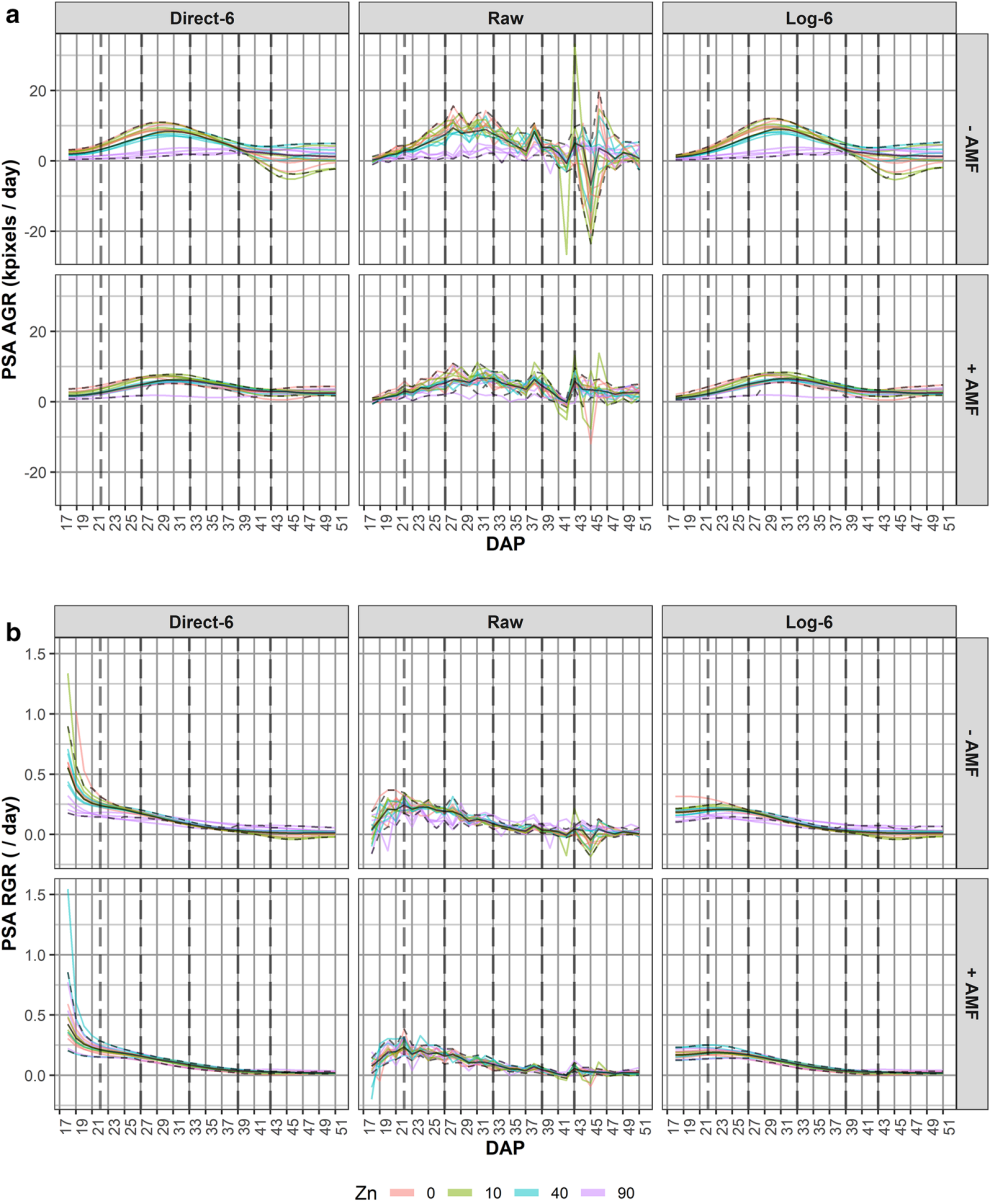
Comparison of the smoothed and raw PSA AGR (**a**) and PSA RGR (**b**) curves for six DF. The left hand panes were obtained by direct smoothing of the PSA RGR values using six smoothing DF, whilst the right hand panes are based on log smoothing. The central panes are derived from the raw, observed data. The vertical lines delineate intervals of reasonably homogeneous growth dynamics observed in the sPSA, sPSA AGR and sPSA RGR. The black line through the profiles is the median profile and the dashed lines are the outer whiskers (points outside the whiskers are potential outliers). The colours differ between the Zn treatments

The smoothing spline DF are somewhat analogous to the degree of a polynomial: smaller smoothing DF result in a smoother fit. Generally, the transient deviations that occur in HTP facilities mandate that a high degree of smoothing be used, typically smoothing DF varying between four and six. Automated techniques for choosing the amount of smoothing, such as cross-validation and mixed model fitting, do not result in sufficient smoothing. Instead, we advocate an evidence-based choice of the amount of smoothing and the smoothing method, with the aid of median-deviations plots of observed minus smoothed values of the PSA, PSA AGR and PSA RGR (Fig. 5). Median values above zero indicate either that the smoothed value is under-estimating the trend or that there is a positive transient effect; median values below zero indicate the opposite. Comparisons of unsmoothed and smoothed profile plots assist in deciding between whether the estimation is faulty or a transitory response has occurred (Fig. 6). The underestimation of the initial PSA values and the overestimation of the initial PSA AGR and PSA RGR with direct smoothing when the degrees of freedom are small is, in our experience, common when the range of the PSA values being smoothed cover a range of two or more orders of magnitude. It seems that this is due to the high degree of smoothing being imposed, in conjunction with the uneven influence of the smoothing penalty over this large range of values. In such cases, log smoothing is likely to be preferred, unless conclusions are to concentrate on time points beyond the first few time points and so the smoothed values for the initial DAPs can be discarded in subsequent analyses. Then the selection of the smoothing method should be based on the behaviour of the methods within the range of primary interest. It can happen in an experiment that there is an abrupt change in the growth rate, for example when the watering regime for at least some of the plants changes from restricted watering to full watering. In such cases the use of segmented smoothing, where the data is divided into subintervals each of which is separately smoothed, may be more appropriate than smoothing the undivided data.

An alternative method of smoothing the data is to fit a mathematical function, such as a three- or four-parameter logistic function. Such nonlinear models are popular [15], and have the advantage that they attempt to represent the underlying mechanism. Pinheiro and Bates [23] catalogue as their advantages that they incorporate parameters (e.g. an asymptote) that have a natural biological interpretation, are more parsimonious in that they involve less parameters, and provide more reliable predictions outside the observed times. Against this, making predictions beyond the observed times is dangerous and is often not a requirement in the context of HTP. Further, the mechanistic property is only of advantage if the response is actually being generated by the hypothesized mechanism. A particular problem for nonlinear models arises when treatments are applied during growth because it is difficult for nonlinear models to cope with the changes in growth rates that ensue. The tomato experiment provides an example of this difficulty, in this case resulting from the unintentional “treatment” due to the interruption to watering at DAP 39. In such cases, and more generally, the semiparametric approach has the advantage that it seeks to follow the observed trend. Further, it has been demonstrated here, and is elaborated upon below, that the semiparametric method is capable of producing a variety of biologically relevant parameters, including a number that are similar to those produced using nonlinear models. The amount of smoothing achieved with nonlinear models is comparable with that achieved with smoothing splines using low smoothing DF. However, as noted previously mathematical functions assume a form to the growth that may not apply in a particular case and a subjective choice of a function is necessary. The tools that have been outlined for choosing the amount and method of smoothing can also be used to assess the applicability of particular mathematical growth functions.

It is emphasized that this smoothing process is a purely descriptive procedure and does not entail formal statistical inference. As a result no attempt is made to formally model the variance of the observed response around the smoothed trend at this stage.

### Profile plots of the smoothed data are a useful descriptive tool and aid outlier identification

Profile plots of the smoothed traits are useful as descriptions of the original longitudinal data, particularly with the addition of loess smooths for each treatment, and as a diagnostic tool. For example, Fig. 2a is presented, in a different form, without loess curves and without the outlier, in Figure 3 in Watts-Williams et al. [11]; profile plots with loess curves are given in Figure 1 in Al-Tamimi et al. [2].

The traces over time of each cart that make up a profile plot (i) show the underlying growth trajectories (ii) display the variability in replicate plants, and (iii) make it easier to identify unusual (outlier) plants, the latter because the plots involve multiple observations of each plant. The R package growthPheno [24] can include outer “whiskers” over time, such as are often included in boxplots; potential outlying data is indicated where lines move outside these whiskers. Other criteria that might be used in deciding if a plant is a potential outlier, and so a candidate for exclusion, include: (i) plants whose sPSA values are below, or above, a specified threshold value in a particular time interval, for example the last few days of imaging; (ii) plants whose sPSA AGR or sPSA RGR values are close to zero when the bulk of plants are not, or plants with excessive or haphazardly fluctuating growth rates. The threshold values may well vary with different treatments. Generally, it is advisable to attempt to verify that something irregular happened with a particular plant before removing it. Thus, lab books and images are examined for possible reasons for a plant’s exceptional behaviour to justify removal.

### Extracting per-cart growth traits using the smoothed profile plots

Here the focus is on extracting PSA-based traits that capture the growth dynamics. For the SET process, smoothed profile plots, like those in Fig. 2, play a central role in determining the traits to be extracted. All three plots are examined to subjectively identify time intervals during each of which growth dynamics appear to be homogeneous. For example, an interval might cover a period in which (i) the AGR was increasing for all plants, (ii) the AGR was increasing at a different rate as compared to the previous interval, (iii) there is no increase in AGR, (iv) biomass did not change, (v) biomass decreased, (vi) the RGR is constant, (vii) there is a limited number of plant groups that differ in their growth dynamics, for instance two groups, one of which is growing rapidly and the other is growing much less during the proposed interval, or (viii) plant behaviour is generally inconsistent. If treatments are applied during imaging then intervals will need to be aligned with their application. For example, a treatment on a single day is likely to result in an interval stopping and another starting with the last imaging before treatment; a period of treatment is likely to necessitate one or more intervals covering the period. Once the intervals are chosen, the sPSA values for the end points of these intervals and the mean sPSA AGR and sPSA RGR for each interval are extracted. Such traits have been successfully used in QTL and GWAS analyses, as published results [2, 6, 7, 9, 25] demonstrate.

Other possible PSA-based extracted traits include the maximum growth rate, the time at which the maximum sPSA was reached or the time at which maximum growth rate was attained. Additional imaging variables, like maximum height and top view convex hull, could be used to form extracted traits. The transpiration use efficiency (TUE) [2], the water use index (WUI) [8] and total and individual leaf length [9] have also been employed as traits. Further, traits could be based on chemical measurements or results from hyperspectral imaging data. All of these traits can be smoothed and have traits extracted over the entire period of imaging or for one or more intervals, in the same manner as the PSA.

### Longitudinal analysis is popular, but producing a valid analysis is challenging

The aim of the longitudinal analysis is to obtain estimates of the time trend for the traits by fitting a model to describe the behaviour of the complete set of values for one of the traits, without any pre-smoothing of the data. The intuitively obvious approach to the analysis of HTP data is longitudinal analysis and it is often what is used, either via nonlinear models or smoothing splines. Such analyses have the advantage that the trend is exhibited continuously.

The longitudinal analyses reported here have two components: an analysis and a prediction component. For the analysis component, both the PSA and the logarithm of the PSA are analysed, the analysis of ln(PSA) only being necessary if the estimation of the PSA RGR is required. For the prediction component, the predicted values over time are obtained for the PSA and ln(PSA) from the results of the analysis component. Then the estimates of the trends over time are obtained for (i) the PSA AGR, using the predicted values from the analysis of the PSA, and for (ii) the PSA RGR, using the predicted values from the analysis of the ln(PSA). For each growth rate (GR), the predicted values for consecutive DAPs are differenced. Calculating the GRs in this way maintains a consistency between a GR and its parent trait and allows the calculation of the standard errors and LSD values from those for the parent trait.

An issue that commonly arises in connection with longitudinal analysis is how to adequately model the variation in the experiments. If this is not achieved then the standard errors and the appraisal of the significance of differences between predictions is likely to be in error. Examination of Fig. 2 reveals that the spread amongst the four replicates in the tomato experiment is not the same over the entire imaging period so that variances that differ between DAPs will need to be incorporated into the model. Also, general experience suggests that correlation between neighbouring DAPs is likely. Thus, these aspects need to be modelled via covariance models. Random regression modelling can be used to model various covariance functions for a particular set of data [26] and this has been extended to RRMS [27]. For example, genetic or phenotypic covariance functions might be modelled by specifying splines for the lines or for the individual subjects in the experiment, respectively. The subject-specific splines model phenotypic covariance. However, subject-specific splines may not provide an adequate description of the phenotypic covariance at the subject level [21, 28] and it may be necessary to allow for unequal variances and correlation between the times as well. Several authors [18–20, 29] have recently advocated the use of RRMs to characterize plant growth. However, the specification of the RRM is somewhat variable. Some authors are under the incorrect impression that there is no need to investigate the correlation structure of the residuals if subject-specific RRMs are used, and even that equal variances between times is unnecessary when using RRMs. Also, it is not necessarily appreciated that RRMs can be fitted at several levels and that only subject-specific RRMs contribute to the modelling of the residual covariance structure. Here FRMSs were used at the treatment level and RRMSs were used at the subject level. However, the incorporation of unequal variance and first-order autoregressive correlation between DAPs in the mixed model was found to be necessary to adequately model the variation in the tomato experiment. Not including them in the model resulted in some overestimation of the standard errors.

Clearly, longitudinal mixed models can be quite complex and fitting them can be quite difficult, even for a relatively simple example like the tomato experiment. Frequently models do not converge to a stable solution and so have to be abandoned, as happened with the tomato example. Also, fitting splines as an integral component of the mixed model was not entirely successful in that the degree of smoothing appeared to be insufficient to deal with the amount of temporal variability in the data, a problem others have noted [18].

### SET-based analyses avoid the complications of RRMS-based analyses, while both result in similar conclusions

The SET-based method, like longitudinal analysis, can be employed in any situation in which there are (at least approximately) normally distributed, longitudinal measurements to be analysed. However, for a SET-based analysis it is necessary that there are observations for a reasonable number of time points, of the order of ten or more. There were 35 in the tomato experiment. While we focus on PSA in this paper, as has already been discussed, the SET technique is not restricted to this trait.

A fundamental difference between SET-based and longitudinal analyses is that the parsimonious description of the growth dynamics in a trait is integral to SET-based analysis. Nonetheless, optionally, summary growth parameters can be extracted post-analysis from the predictions obtained from a longitudinal analysis, including for specific intervals, as is done in a SET-based analysis. However, the significance of differences in the parameters is generally limited to those that are linear functions of the analyzed response and producing them may be convoluted. A SET-based analysis is a more straightforward option.

There is an intrinsic efficiency to analyzing intervals as compared to individual time points in that neighbouring time points are often highly correlated (for the tomato experiment, the estimated correlation for neighbouring time points was 0.88 and 0.77 for time points with a single intervening time point). Consequently, the results of neighbouring time points will be similar and there will be a larger number of hypothesis tests so that more falsely significant conclusions are likely.

Comparison of Figs. 3 and 4 reveals that the SET-based analysis has picked up essentially the same trends in the traits as the longitudinal analysis, albeit on a coarser scale, and results in similar conclusions. Crucial to achieving this is the choice of intervals for the SET-based analyses.

Not only are the results similar, but some of the problems noted for a longitudinal analysis are avoided in a SET-based analysis. The pre-smoothing of the data allows the imposition of a greater degree of smoothing so that more of the transitory variation is removed. Because analyses are performed on per-cart traits, convergence of the fitting process is much less of an issue and the technique is computationally cheap. Valid results are more likely because the need to deal with correlation between times is avoided and heterogeneous variances across times is automatically accommodated. The analysis of individual time points, as is proposed here and in Kwak et al. [17], may have the problem that the correlation between time-points is not taken into account [18]; similar comments apply to a series of time intervals. However, it would appear, from the results of the study reported in Additional file 2 that, as long as comparisons are limited to predictions at the same time, then the proposed separate analyses are valid. However, one still needs to be alert to the possibility of unequal variances arising from different treatments; this often occurs with well-watered as compared to restricted watering of plants for example.

It also may be argued that the longitudinal analysis has the advantage of utilizing all of the data. However, the pre-smoothing process that is part of the SET-based analysis also uses all of the data. As for summarizing growth rates within intervals using interval estimates of growth rates for smoothed data, the proposed estimator is the weighted average of that data for all of the time points in the interval. It is true that, for an interval, this simplifies mathematically to the difference between the smoothed values for the interval end points. However, neighbouring observed values have contributed to these values through the smoothing process and anomalous time points can be avoided by ensuring that they are not chosen as end points for intervals. In any case, the same calculations would be performed on predictions from longitudinal analyses to produce equivalent interval growth rates.

### Choosing between a SET-based and a longitudinal analysis

Which of these two methods is used to analyze the longitudinal data from an experiment will depend upon the objectives of the experiment, the practicalities of the situation and the preferences of the researchers. A SET-based analysis is better suited than longitudinal analysis to situations in which at least one of the following applies: (i) an objective is to provide a parsimonious description of growth, perhaps, focusing on differences in specific intervals (ii) biologically relevant traits are to be extracted for subsequent analysis, a common objective of the functional analysis of growth data that our method also facilitates, or (iii) an analysis that is computationally less demanding or based on a simpler model, while still capturing the essential features of the exhibited growth dynamics, is wanted. A longitudinal analysis is appropriate when the objective is solely to characterize the broad growth trajectory and one is able or prepared to undertake the analysis. The SET-based technique has been used successfully in a number of published analyses [2, 3, 6–9, 11, 25, 30]. On the other hand, a difficult longitudinal analysis was used when a broad description of the growth trajectories with error intervals for the different treatments was required [31].

### Subjectivity in growth analyses

Subjectivity in growth analyses arises in choosing an analysis method and in the models employed in the analysis:

1. It occurs at the outset of a growth analysis with the need to decide whether a SET-based or a longitudinal analysis is to be conducted. As we discussed in more detail above, the objectives of the researchers and their preferences have a role to play in making this decision.
2. A subjective element is involved, for both SET-based and longitudinal analyses, in the choice of the models to be used to describe (i) the growth trend and (ii) the variation in the experiment. For modelling the growth trend, the objective is to identify a model that follows the underlying growth trajectory as accurately as possible. For this, there is the subjective choice between nonparametric smoothing and growth models in the form of mathematical functions. For nonparametric functions, subjectivity arises in selecting the method of smoothing and the associated smoothing parameters. For growth models, the particular form of mathematical function is often subjectively selected. We have suggested median deviations plots as a means of making these choices evidence-based. The choice of a variation model is somewhat more vexed, especially for a longitudinal analysis. The model needs to account for spatial variation in the data, unequal variance between treatments and correlation and unequal variances between time points. Because the occurrence of these different forms of variation varies between experiments, a set of potential models that cover the anticipated forms of variation in an experiment is subjectively identified. Choosing a model from this set that is appropriate to describing the data can be based on hypothesis tests and diagnostic plots of residuals.
3. It may be an objective of the analysis to produce estimates of growth parameters in order to obtain a parsimonious description of specific aspects of growth with a view to comparing different lines and/or treatments with respect to these parameters. When a mathematical function is used to model the growth, the possible growth parameters are often restricted to those based on the parameters for the selected growth model, As an example, the parameters for the three-parameter logistic described in “Methods” section under the heading “Data smoothing” are the upper asymptote, the time at which half the asymptote is reached and a scale parameter that is approximately the time elapsed between reaching half and three-quarters of the asymptote. In addition to the subjectivity involved in selecting a mathematical function, the choice of one or more of these parameters, or others derived from them, as summaries of particular aspects of growth has a subjective component. When splines are used to model the growth, the number of growth parameters available is considerable, as has been outlined in the discussion of the extraction of per-cart growth traits. In the same manner as for choosing aspects of growth to summarize when mathematical functions are used to model growth, subjectivity applies in making these choices when splines are used to model growth.

## Conclusions

We have compared two methods for analyzing data from HTP facilities: SET-based and longitudinal analyses, both of which used cubic smoothing splines to describe the trend in the traits of interest over time, although the SET-based analysis also investigated the use of a logistic curve.

It has been demonstrated that features of the SET-based method include that it focusses attention on specific time periods and produces traits that can be subject to further analysis. SET-based analyses are easier to perform than longitudinal analyses and the technique is flexible, efficient, valid and widely applicable. Thus, the SET-based analysis will appeal to those researchers who want to perform a valid growth analysis without having to contend with the complications associated with longitudinal analyses, or for whom focusing on growth in distinct time periods will allow them to test relevant scientific hypotheses. The SET-based analysis has been successfully employed for several published phenotypic analyses [2, 3, 6–9, 11, 25, 30]. We have developed the R package growthPheno [24] that facilitates the SET.

On the other hand, if a traditional analysis is chosen, it is necessary to carefully consider the modelling of the variation in the experiment. This is crucial to ensuring that the *p*-values used in model selection and hypothesis testing are correct and in obtaining accurate estimates of the standard errors to be employed in assessing prediction differences.

## Methods

### Plant growth and data acquisition

The 32 pots involved in the experiment were placed into 32 carts on the conveyor system within a Smarthouse at the Australian Plant Phenomics Facility, University of Adelaide, where they occupied two lanes by 16 positions. There were four replicates of each treatment and a randomized complete-block design was used to assign the treatments. The plants were imaged daily from 17 to 51 DAP using RGB cameras. From these images the PSA of the plant was obtained by summing the areas as measured (in kilopixels or kpixels) from two side views at an angular separation of 90° and a view from above [2]. This resulted in 1120 PSA values, which are used as a measure of plant biomass, having been shown to be related to plant fresh weight for numerous species [2, 12, 32–34]. Note that watering was unintentionally interrupted for 3 days (39–41 DAP inclusive).

### Calculating growth rates

The AGR and RGR between two time points, *t*_*j*_ followed by *t*_*k*_, can be calculated as follows [14]:

1 $$AGR_{{\left( {t_{j} ,t_{k} } \right)}} = \frac{{PSA_{{t_{k} }} - PSA_{{t_{j} }} }}{{t_{k} - t_{j} }}\;\;\;{\text{and}}\;\;RGR_{{\left( {t_{j} ,t_{k} } \right)}} = \frac{{\ln \left( {PSA_{{t_{k} }} } \right) - \ln \left( {PSA_{{t_{j} }} } \right)}}{{t_{k} - t_{j} }}$$

If there are observations at several time points between *t*_*j*_ and *t*_*k*_, it can be proved that the weighted mean of the AGRs and RGRs for all pairs of observed time points in the interval *t*_*j*_ to *t*_*k*_, is given by the formulae in (1), when the weight for each GR being averaged is the time range for each GR. Such a GR is referred to as an interval GR and those for successive time points are called continuous GRs. That is, the AGR and RGR for a time interval from *t*_*j*_ to *t*_*k*_ that covers several subintervals are given by Eq. (1). The formulae can be adapted to traits other than PSA. Thus, if a WUI has been obtained for a set of time points, then the continuous and interval WUIs can be derived from the AGR formula. This method avoids the problematic assumption of a homogeneous GR for the interval, as required for the use of linear regression as outlined in Paine et al. [15].

The growth rates can also be calculated using the first derivatives of the smoothing splines. However, not all software provides them and they are more complicated to use, especially for obtaining interval GRs. Further, differencing is the only way to calculate observed or raw GRs.

### SET procedures

**Fig. 7:**
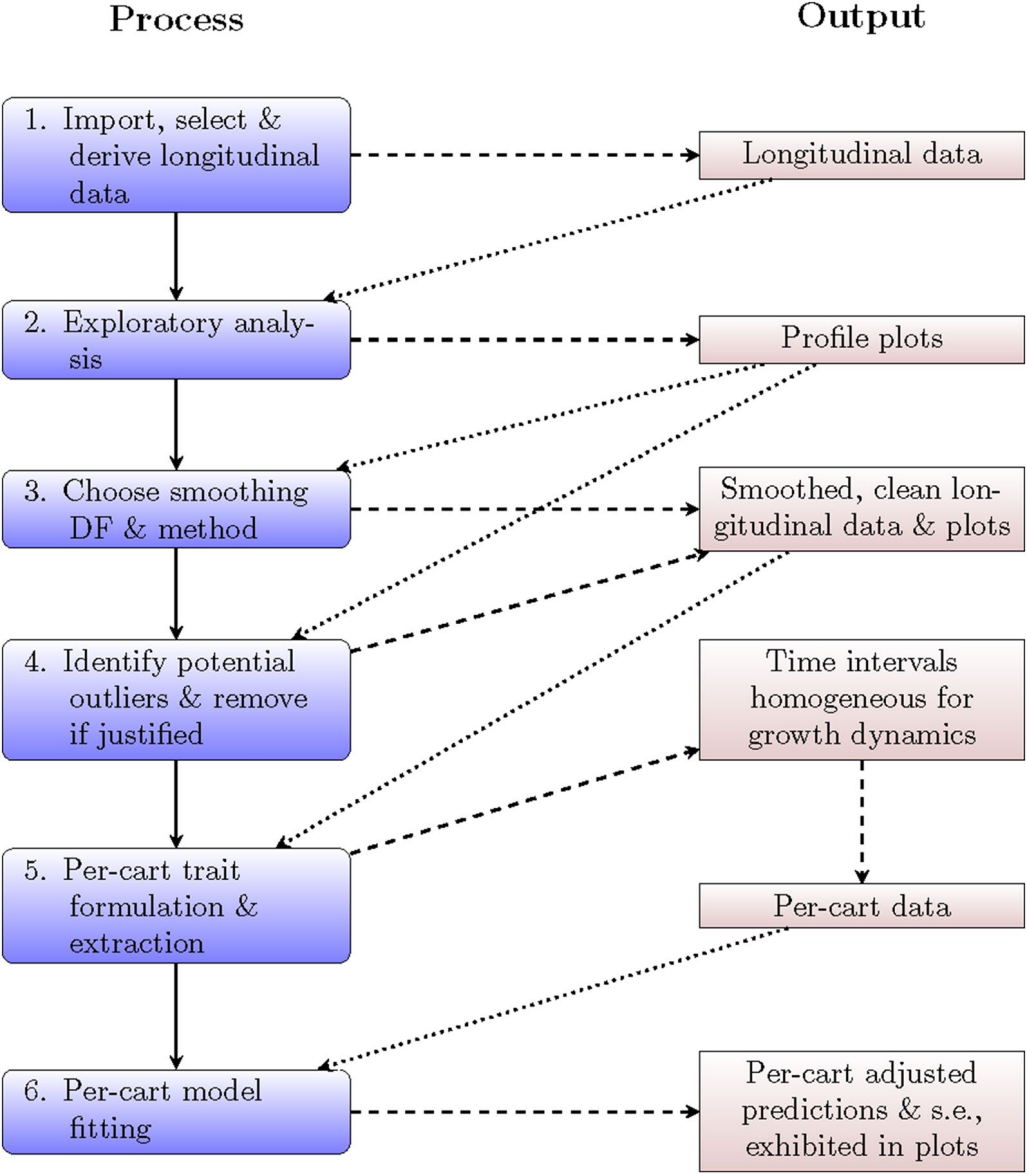
Outline of the SET-based analysis processes and outputs. The first five steps are the SET and the remaining set is the analysis step. The solid lines indicate a move onto the next step in the process, the dashed lines indicate the production of outputs by the process and the dotted lines indicate input into a step in the process

A SET-based analysis, as exemplified for the tomato data, involved the six steps shown in Fig. 7. The first five of these steps, the SET, amount to data preparation for the remaining analysis step. In the SET, the raw data is explored, smoothed and cleaned to obtain a data set that has had transient deviations from trend removed by the smoothing and has had outlying plants that can be identified as being unambiguously flawed, removed They are graphics intensive, as is also advocated by others [15].

The SET was done using growthPheno [24] and nlme [35], both of which are packages for the R statistical computing environment [36]. The functions used from these packages are those that allow the simultaneous processing of all plants from an experiment. They require a single column for (i) a plant identifier, (ii) the times for each observation, (iii) each of the factors specifying the treatment associated with an observation, and (iv) each of the traits. The package growthPheno has options that allow the specification of the handling of missing values. While the traits that we are using as examples are botanical in nature, the software is not restricted to processing this type of trait.

#### Import, select and derive longitudinal data

The data from processing the images of the plants in the 32 carts in the tomato experiment over DAP 17–51 was supplied in an Excel file and imported into R [36]. The trait of interest is the projected shoot area (PSA); the PSA values were used to derive the continuous absolute growth rates (AGRs) and relative growth rates (RGRs) for adding to the data, a continuous growth rate (GR) being a GR obtained for each time of imaging. Based on Eq. (1), the continuous AGR was calculated by taking the difference between successive PSA values and the continuous RGR calculated by taking the difference between the logarithms of successive values, the time difference being one day; a value for the first imaging time is not produced for either GR.

#### Exploratory analysis

Profile plots for PSA, PSA AGR and PSA RGR have been produced for the exploratory analysis, a profile plot for a trait containing a line for the trait values over time for each cart.

#### Data smoothing

The PSA values are to be smoothed either by fitting natural cubic smoothing splines or by fitting a three-parameter logistic function to the data for each cart. For smoothing using splines, there is a choice of two methods: (i) log-smoothing for which splines are fitted to the natural logarithms of the PSA values for each cart and then backtransforming the fitted values, i.e. by taking the exponentials of the fitted values; (ii) direct smoothing, for which the spline is fitted to the untransformed data. In addition, the smoothing DF must be specified. The equation for a three-parameter logistic function is given by

$$PSA_{t} = {{\phi_{1} } \mathord{\left/ {\vphantom {{\phi_{1} } {\left\{ {1 + \exp \left[ {{{ - \left( {t - \phi_{2} } \right)} \mathord{\left/ {\vphantom {{ - \left( {t - \phi_{2} } \right)} {\phi_{3} }}} \right. \kern-\nulldelimiterspace} {\phi_{3} }}} \right]} \right\}}}} \right. \kern-\nulldelimiterspace} {\left\{ {1 + \exp \left[ {{{ - \left( {t - \phi_{2} } \right)} \mathord{\left/ {\vphantom {{ - \left( {t - \phi_{2} } \right)} {\phi_{3} }}} \right. \kern-\nulldelimiterspace} {\phi_{3} }}} \right]} \right\}}},$$

where *ϕ*_1_ is the upper asymptote, *ϕ*_2_ is the time at which half the asymptote is reached and *ϕ*_3_ is a scale parameter that is approximately the time elapsed between reaching half and three-quarters of the asymptote. It is noted that, if there is missing data for one or more time points, imputed smoothed values can be obtained for them.

To choose the smoothing method and smoothing DF for the tomato experiment, median-deviations plots were generated for smoothed data obtained from the PSA, PSA AGR and PSA RGR by (i) both direct and log smoothing in combination with four, five, six and 12 smoothing DF, and (ii) the fitting of a three-parameter logistic curve.

The median deviations plots for PSA are in Fig. 5, while the complete set is in Additional file 1: Figures S1–S3. For these plots, the median deviations are obtained by (i) calculating the deviation, observed minus smoothed value, for each plant at each time point, and (ii) calculating the median of the deviations at a single time point for the plants to be plotted in a single pane of the graph. One would expect the magnitude of the deviations to increase as the smoothing DF decreases, because there is, as ever, a trade-off between the amount of smoothing and the magnitude of the deviations from the observed trend.

The median deviations of the observed PSA values from directly smoothed PSA values for four, five and six smoothing DF deviate markedly from the observed data, especially prior to DAP 31; the median deviations for the logistic also have larger negative values up to DAP 23, especially for the +AMF treatment. The deviations in this earlier period have many values that are in excess of 10% of median smooth PSA values. It seems that direct smoothing with these DF underestimates the initial trend and the logistic overestimates it. Log smoothing with smoothing DF equal to four or five and the logistic tend to produce the largest deviations post DAP 25, while the logistic unsurprisingly produces large deviations post DAP 47. In these latter periods overestimation seems to be occurring. On the other hand, Additional file 1: Figures S2 and S3, reveal that the PSA AGR and PSA RGR are underestimated prior to DAP 23, particularly when direct smoothing is used with small smoothing DF. This is to be expected given that the PSA values range from 1.75 to 185.50 kpixels.

Taking into account all of the plots presented in Additional file 1, log smoothing with six smoothing degrees of freedom is chosen for representing the PSA trend over DAPs 17–51. This differs from the previously reported analysis [11], where direct smoothing with six smoothing DF were used because the statistical analyses focused on DAP 27–43. To confirm this choice, a comparison of the results of direct and log smoothing for PSA, PSA AGR and PSA RGR when six smoothing DF are used is made using Additional file 1: Figures S4–S6, with the comparison for PSA AGR and PSA RGR also shown in Fig. 6. These profile plots have the feature that the medians of the data for each pane of the plot are included as a black line and the outer “whiskers” are included as dashed black lines. The lower (upper) outer whisker is the median minus (plus) 1.5 times the interquartile range, the interquartile range being the 75th quartile minus the 25th quartile of the data. Points outside the outer whiskers are regarded as potential outliers. The largest difference between the two smoothing schemes occurred with sPSA RGR, where direct smoothing with small smoothing DF is clearly over-estimating the RGR in the first 3 days. The effect of the unintentional watering interruption from DAP 39–43 on subsequent growth is apparent, especially in the GRs. This is a situation in which segmented smoothing may have been appropriate. However, the smoothed trend lines for log smoothing with six DF appear to follow adequately the overall trend through this period. Besides, smoothing a short interval, such as DAP 43–51, is problematic and in this case there is a large decrease in the PSA AGR for DAP 44, especially for −AMF.

#### Data cleaning

In the tomato experiment, there was a plant in the +AMF treatment that was obviously much slower growing than other plants (see Additional file 1: Figures S4 and S5, where this plant is outside the lower outer whisker at times). It was a small plant whose AGR was low, but whose RGR was similar to other plants and so does not show in Fig. 6. It had low AMF root colonization and a random mutated shoot phenotype, which could explain why its behaviour was consistent with a plant that was not inoculated with AMF. As before [11], we have omitted the plant from the analyses reported here.

#### Extracting per-cart growth traits

The plots in Fig. 2 and the Log-6 plots in Fig. 6 and Additional file 1: Figures S4–S6 were examined by statisticians and researchers to subjectively identify time intervals during each of which growth dynamics appear to be homogeneous. The vertical dashed lines in Additional file 1: Figures S4–S6 and in Fig. 6 mark the chosen intervals: DAP 18–22 was a period of increasing AGR; DAP 22–27 was a period of even faster increases in the AGR; DAP 27–33 was a period during which the AGR peaked; DAP 33–39 shows a relatively constant rate of decrease in the AGR; DAP 39–43 is the period of restricted watering and followed by a 2-day recovery period; DAP 43–51 was a period in which the AGR is flat but fluctuating wildly. Traits can be formed from the values of sPSA, sPSA AGR and sPSA RGR for time-interval end points and the mean values of the sPSA AGR and sPSA RGR over each of the time intervals. Thus, there are potentially 33 extracted traits:

1. sPSA, sPSA AGR and sPSA RGR at DAPs 18, 22, 27, 33, 39, 43, 51;
2. the sPSA AGR and sPSA RGR for each of the intervals DAP 18–22, 22–27, 27–33, 33–39, 39–43, 43–51.

#### Analyzing per-cart growth traits

Of the 33 extracted traits, those for sPSA AGR and sPSA RGR at DAP end points will be omitted, leaving 19 extracted traits to be analyzed. The per-cart model fitting involves fitting a mixed model to each of these traits and incorporates terms to account for (i) the spatial variation that affected the experiment and (ii) the effect of treatments on the phenotypic response. The mixed model is of the following form:

2 $${\mathbf{y}} = {\mathbf{X{\varvec{\upbeta}}}} + {\mathbf{Zu}} + {\mathbf{e}}$$

where **y** is the response vector of values for the trait being analysed, **β** is the vector of fixed effects, **u** is the vector of random effects, and **e** is the vector of residual effects. Both **X** and **Z** are design matrices, corresponding to **β** and **u**, respectively.

For the maximal model for a per-cart trait for the tomato experiment, the fixed-effect vector **β** is partitioned into subvectors as follows: $$\left[ {\begin{array}{*{20}c} \mu & {{{\varvec{\upbeta}}}_{{\text{B}}} } & {{{\varvec{\upbeta}}}_{{\text{Z}}} } & {{{\varvec{\upbeta}}}_{{\text{A}}} } & {{{\varvec{\upbeta}}}_{{{\text{ZA}}}} } \\ \end{array} } \right]$$, where *μ* is the overall mean parameter and the **β** subvectors are, respectively, the subvectors of Block (B) parameters, Zn (Z) parameters, AMF (A) parameters, and parameters for Zn-AMF combinations (ZA). There are no random effects and the residual vector **e** is assumed to be normally distributed with mean vector **0** and variance *σ*^2^**I**_32_, where *σ*^2^ is the residual variance and **I**_32_ is the identity matrix of order 32 i.e. the residuals are independently distributed with the same variance for all. To check this latter assumption residual-versus-fitted-values and normal probability plots were obtained for all traits (see Additional file 1: Figures S7–S25). There was no evidence of substantial departure from the equal variance and normality assumptions.

The R packages asreml [37] and asremlPlus [38] were used to fit the mixed model to the 19 extracted traits. Wald *F*-statistics, with Kenward and Roger [39] calculation of their denominator degrees of freedom, were obtained to assess the significance of the fixed effects involving Zn and AMF. These packages were also used to produce predicted values and their standard errors, the latter used to compute LSD (*α* = 0.05) values for comparing pairs of predicted values.

An additional analysis was performed for each of sPSA, sPSA AGR and sPSA RGR. For each, their extracted traits were combined and a joint analysis conducted.

The following tips are offered, based on our experience with using the SET method:

1. When the smoothing DF are two, a straight line is fitted, and when the smoothing DF equals the number of observed time points the fitted curve goes through all of the observed points ([40], Section 3.3.4). Hence, the smoothing DF are usually greater than two and less than the number of observed time points, the actual value depending on the amount of smoothing desired.
2. When looking for bias arising from the smoothing of individuals, perhaps using the median deviations plots, pay particular attention to either end of the observed range of times and when there are sudden changes in trend.
3. Collect data before and after the times during which the interest in growth is concentrated. Always smooth all of the data for a plant, even if interest is restricted to a subinterval of the observed times. One reason for this is to avoid the bias in the smoothing that can occur at either end of the period of observation.
4. When examining deviations plots it is expected that there will be periodic patterns in the deviations, these being due to the removed transient effects.
5. Facet profile plots so that differences between the facets are maximized.
6. Choose intervals based on the growth dynamics, it not being necessary for the intervals to be equal in size. Although the original trait and its AGR and RGR should be examined, it often happens that the AGR is the most important guide. Focus on broad patterns in the trends.
7. Examine the profile plots for likely sources of differential variance and for anomalous individuals.

### Longitudinal analysis

The aim of the longitudinal analysis was to obtain estimates of the trend over time of the PSA, PSA AGR and PSA RGR for each combination of Zn and AMF by fitting a model to describe the behaviour of the complete set of values for one of the traits, without any pre-smoothing of the data. Only the first two and the fourth steps of a SET analysis were relevant to the longitudinal analysis, because it was carried out on the raw, cleaned data. It has two components: an analysis and a prediction component. For the analysis component, both the PSA and the logarithm of the PSA are analysed, the analysis of ln(PSA) only being necessary if the estimation of the PSA RGR is required. For the prediction component, the predicted values over time are obtained for the PSA and ln(PSA) from the results of the analysis component. The estimates of the trends over time are obtained for (i) the PSA AGR, using the predicted values from the analysis of the PSA, and for (ii) the PSA RGR, using the predicted values from the analysis of the ln(PSA). For each GR, the predicted values for consecutive DAPs are differenced. Calculating the GRs in this way maintains a consistency between a GR and its parent trait and allows the calculation of the standard errors and LSD values from those for the parent trait.

In the analysis component, as in the per-cart analysis, splines were used to describe the trend. Variation at the cart (subject) level was modelled using (i) RRMSs, in which random nonlinear trends between individual carts were specified by fitting natural cubic smoothing splines with random intercepts and slopes, with a knot per observed DAP and homogeneous splines fitted so that the amount of smoothing was the same for all carts, (ii) unequal cart variance between DAPs, and (iii) first-order autoregressive correlation between different DAPs. An FRMS was used to describe the trend over time for each combination of Zn and AMF; it employed heterogeneous splines, that is, splines for which the amount of smoothing was allowed to differ between the combinations. However, the same number of equally spaced knots was specified for these splines, although four analyses were conducted with one of 10, 15, 20 or 35 knots.

The mixed model on which the analysis of these two traits is based is of the form given in Eq. (2). The maximal model for this analysis includes a subject-specific RRMS, a subject being a Block-Cart combination. It has the fixed-effect vector **β** partitioned into subvectors as follows: $$\left[ {\begin{array}{*{20}c} \mu & {{{\varvec{\upbeta}}}_{{\text{B}}} } & {{{\varvec{\upbeta}}}_{{\text{Z}}} } & {{{\varvec{\upbeta}}}_{{\text{A}}} } & {{{\varvec{\upbeta}}}_{{{\text{ZA}}}} } & {{{\varvec{\upbeta}}}_{{\text{D}}} } & {{{\varvec{\upbeta}}}_{{{\text{DB}}}} } & {{{\varvec{\upbeta}}}_{{{\text{DZ}}}} } & {{{\varvec{\upbeta}}}_{{{\text{DA}}}} } & {{{\varvec{\upbeta}}}_{{{\text{DZA}}}} } \\ \end{array} } \right]$$, where *μ* is the overall mean parameter and the **β** subvectors are, respectively, the subvectors of Block (B) parameters, Zn (Z) parameters, AMF (A) parameters, parameters for Zn-AMF combinations (ZA), DAP (D) parameters, parameters for DAP-Zn combinations (DZ), parameters for DAP-AMF combinations (DA), and parameters for DAP-Zn-AMF combinations (DZA). The incidence matrix **X** is partitioned to conform to the partition of **β**. That is, the maximal fixed model involves a full three-factor interaction model for the factors Zn, AMF and DAPs, which provides an unsmoothed representation of the differences in the trend over the DAPs for the different Zn-AMF combinations. To specify the RRMS, and using “xD” to signify a centred, numeric covariate for the categorical factor DAPs, the random effects vector **u** is partitioned as $$\left[ {\begin{array}{*{20}c} {{\mathbf{u}}_{{{\text{BC}}}} } & {{\mathbf{u}}_{{{\text{BCxD}}}} } & {{\mathbf{u}}_{{\text{BCspl(xD)}}} } \\ \end{array} } \right]$$, where **u**_BC_ is the subvector of Block-Cart random effects, **u**_BCxD_ is the subvector of random DAP slopes over xDAP for each Block-Cart combinations and **u**_BCspl(xD)_ is the subvector of random spline coefficients of the spline basis functions for xDAP for each Block-Cart combination. The incidence matrix **Z** is partitioned to conform to the partition of **u**. The vector $$\left[ {\begin{array}{*{20}c} {{\mathbf{u}}_{{{\text{BC}}}} } & {{\mathbf{u}}_{{{\text{BCxD}}}} } \\ \end{array} } \right]$$ is assumed to be normally distributed with mean vector **0** and variance ${{\varvec{\Sigma}}}_{{{\text{xD}}}} \otimes {\mathbf{I}}_{32}$, where ${\varvec{\Sigma}}$_xD_ is 2 × 2 matrix specifying the variances and covariance of the intercepts and slopes and **I**_32_ is an identity matrix of order 32, the number of Block-Cart combinations. The vector **u**_BCspl(xD)_ is assumed to be normally distributed with mean vector **0** and variance $\sigma_{{\text{BCspl(xD)}}}^{2} {\mathbf{G}}_{{\text{BCspl(xD)}}}$, where **G**_BCspl(xD)_ is a matrix derived from the knot points for the fitted spline. The residual vector **e** is assumed to be normally distributed with mean vector **0** and variance ${\varvec{\Sigma}}$_1120_, where, for **y** ordered by Block-Cart then DAP, all elements are zero except for 32 diagonal blocks, one for each Block-Cart combination, The variance matrix for the *i*th Block-Cart, ${\varvec{\Sigma}}_{i}$, is 35 × 35 and allows for different variances for different DAPs and first-order autoregressive correlation between DAPs i.e. correlation that decreases according to a power law as the number of intervening DAPs between a pair of DAPs increases. Formally,

$${{\varvec{\Sigma}}}_{i} = \left[ {\begin{array}{*{20}c} {\sigma_{1} } & 0 & \cdots & \cdots & 0 & 0 \\ 0 & {\sigma_{2} } & \cdots & \cdots & 0 & 0 \\ \vdots & \vdots & \ddots & {} & \vdots & \vdots \\ \vdots & \vdots & {} & \ddots & \vdots & \vdots \\ 0 & 0 & \cdots & \cdots & {\sigma_{34} } & 0 \\ 0 & 0 & \cdots & \cdots & 0 & {\sigma_{35} } \\ \end{array} } \right]\left[ {\begin{array}{*{20}c} 1 & {\rho^{1} } & \cdots & \cdots & {\rho^{33} } & {\rho^{34} } \\ {\rho^{1} } & 1 & \cdots & \cdots & {\rho^{32} } & {\rho^{33} } \\ \vdots & \vdots & \ddots & {} & \vdots & \vdots \\ \vdots & \vdots & {} & \ddots & \vdots & \vdots \\ {\rho^{33} } & {\rho^{32} } & \cdots & \cdots & {\sigma_{34} } & {\rho^{1} } \\ {\rho^{34} } & {\rho^{33} } & \cdots & \cdots & {\rho^{1} } & {\sigma_{35} } \\ \end{array} } \right]\left[ {\begin{array}{*{20}c} {\sigma_{1} } & 0 & \cdots & \cdots & 0 & 0 \\ 0 & {\sigma_{2} } & \cdots & \cdots & 0 & 0 \\ \vdots & \vdots & \ddots & {} & \vdots & \vdots \\ \vdots & \vdots & {} & \ddots & \vdots & \vdots \\ 0 & 0 & \cdots & \cdots & {\sigma_{34} } & 0 \\ 0 & 0 & \cdots & \cdots & 0 & {\sigma_{35} } \\ \end{array} } \right]$$

where $\sigma_{j}$ is the cart standard deviation on the *j*th DAP and $\rho^{{\left| {t_{k} - t_{j} } \right|}}$ is the correlation between DAPs *t*_*k*_ and *t*_*j*_, being the correlation between consecutive DAPs, *ρ*, raised to the power equal to the number of DAPs between the two DAPs. In terms of variation, this model allows for Block differences, random variation between carts, random variation between carts in the curved trend over the DAPs that each follows and random deviations from this trend on a particular DAP for a particular cart; this last variation varies from one DAP to the next and there is correlation between observations on different DAPs that is strongest for consecutive DAPs.

The model selection strategy for a response proceeded in stages as follows:

1. *Select the model to describe the spatial and temporal variation present in the response* The maximal mixed model was fitted to the response. Then tests of whether the random model could be simplified were carried out: is unequal variance between times needed? Is the autocorrelation significant? Can the spline component for variation in time trends between carts be removed to leave a random linear term? If variance components were estimated to be very close to zero, they were either removed, if they were single-component terms, or fixed at $1 \times 10^{ - 4}$, if they were one of the DAP variances. The latter is justified in that it is indicating that the variation in variance for that DAP is derived entirely from variance in the curved trend. Unsmoothed predictions for the combinations of Zn, AMF and DAPs are obtained from this model.
2. *Explore the amount of smoothing to apply to the FRMS describing the time trend of each combination of Zn and AMF* For this, the three-factor model for Zn, AMF and DAPs was reparameterized to specify an FRMS that includes fixed linear and random spline terms, based on xDAP a centred, numeric covariate for the DAPs, and random deviations terms for DAPs; heterogeneous spline terms for each combination of Zn and AMF were specified leading to separate variance components for each combination and hence different amounts of smoothing for them. That is, the partition of **β** was modified to $$\left[ {\begin{array}{*{20}c} \mu & {{{\varvec{\upbeta}}}_{{\text{B}}} } & {{{\varvec{\upbeta}}}_{{{\text{ZA}}}} } & {{{\varvec{\upbeta}}}_{{{\text{xDZA}}}} } \\ \end{array} } \right],$$ where the **β**_ZA_ contains the intercept parameters for each combination of Zn and AMF and **β**_xDZA_ contains the fixed slope parameters over xDAP for each combination of Zn and AMF. The random vector **u** is partitioned into $$\left[ {\begin{array}{*{20}c} {{\mathbf{u}}_{{\text{het(ZA)spl(xD)}}} } & {{\mathbf{u}}_{{{\text{ZAD}}}} } & {{\mathbf{u}}_{{{\text{BC}}}} } & {{\mathbf{u}}_{{{\text{BCxD}}}} } & {} \\ \end{array} {\mathbf{u}}_{{\text{BCspl(xD)}}} } \right]$$, where **u**_het(ZA)spl(xD)_ is the subvector of random spline coefficients of the spline basis functions for xDAP with heterogeneous variance components for each Zn-AMF combination and **u**_ZAD_ is the subvector of random deviations from the fitted curved trend for each Zn-AMF-DAPs combinations The degree of smoothing was altered by fitting the FRMS with 10, 15, 20 or 35 equally-spaced knots for the Zn by AMF by spl(xDAP) term, **u**_ZAspl(xD)_. For each fit, a test for the significance of the random deviations was conducted.
3. *Choose the number of knots by obtaining the predictions for different amounts of smoothing for each trait and calculate GRs from each set of predictions* The predictions for the observed values of xDAP in combination with the levels of Zn and AMF were obtained, along with the predictions and LSD (5%) for comparing predictions at the same DAP. The predictions and half-LSD intervals were plotted for each of the four knot numbers and subjectively compared. The calculation of the predicted growth rates were obtained by taking differences between consecutive predictions of either the PSA or the ln(PSA). The LSD (5%) for the predicted growth rates were calculated from those of the corresponding primary response, PSA or ln(PSA). Also, to examine the fit of these (smoothed) predictions, plots of the trend deviations were produced, the trend deviations being the difference between the unsmoothed predictions and the smoothed predictions. These are analogous to the median deviations used in the SET.
4. *Look to simplify the FRMS describing the effect of Zn and AMF on the trend over DAPs* Firstly, for the chosen number of knots, a test of significance was conducted to determine if the ZAD random deviations were significant; if they were not significant, they were dropped from the model. Then tests of significance were made to compare models with heterogeneous spline terms for each Zn-AMF combination, heterogeneous spline terms for each AMF level, heterogeneous spline terms for each Zn level and a single, homogeneous spline term across all Zn-AMF combinations. If none of the models with heterogeneous spline terms was significant, then a third parameterization of the model was fitted, this one incorporating interactions between (i) Zn, (ii) AMF, and (iii) the Zn by AMF interaction with xDAP and spl(xDAP) and DAPs, where each spline term has a single, homogeneous spline component. In this case the partition of **β** was modified to $$\left[ {\begin{array}{*{20}c} \mu & {{{\varvec{\upbeta}}}_{{\text{B}}} } & {{{\varvec{\upbeta}}}_{{\text{Z}}} } & {{{\varvec{\upbeta}}}_{{\text{A}}} } & {{{\varvec{\upbeta}}}_{{{\text{ZA}}}} } & {{{\varvec{\upbeta}}}_{{{\text{xDZ}}}} } & {{{\varvec{\upbeta}}}_{{{\text{xDZA}}}} } & {{{\varvec{\upbeta}}}_{{{\text{xDZA}}}} } \\ \end{array} } \right]$$ and that for the random vector **u** to $$\left[ {\begin{array}{*{20}c} {{\mathbf{u}}_{{\text{Zspl(xD)}}} } & {{\mathbf{u}}_{{\text{Aspl(xD)}}} } & {{\mathbf{u}}_{{\text{ZAspl(xD)}}} } & {{\mathbf{u}}_{{{\text{ZD}}}} } & {{\mathbf{u}}_{{{\text{AD}}}} } & {{\mathbf{u}}_{{{\text{ZAD}}}} } & {{\mathbf{u}}_{{{\text{BC}}}} } & {{\mathbf{u}}_{{{\text{BCxD}}}} } & {} \\ \end{array} {\mathbf{u}}_{{\text{BCspl(xD)}}} } \right]$$. The difference between this and the previous parameterization is that main effect terms for Zn and AMF have been included for each of the intercepts, slopes and splines. For each response, a test of significance was conducted to determine if the ZAD random deviations were significant; if they were not significant, they were dropped from the model and tests of the ZD and MD random deviations conducted, dropping any nonsignificant deviations. On completion of these tests, a test was conducted to establish whether the ZAspl(xD) spline term was significant; if it was not significant, it was dropped from the model and tests of the Zspl(xD) and Mspl(xD) spline terms conducted, dropping any nonsignificant spline terms. The predictions from the fitted models were obtained for the Zn-AMF combinations over the observed DAPs and the growth rates calculated from them.

Again, the R packages asreml [37] and asremlPlus [38] were used to fit the mixed models to both response variables. Testing for variance terms used Restricted Maximum Likelihood Ratio Tests (REMLRT), the calculation of the *p*-value being adjusted when the test involved a variance component constrained to be nonnegative [21]. Wald *F*-statistics, with Kenward and Roger [39] calculation of their denominator degrees of freedom, were obtained to assess the significance of the fixed effects. These packages were also used to produce predicted values and their standard errors, the latter used to compute LSD (*α* = 0.05) values for comparing pairs of predicted values.

To investigate the adequacy of the fitted models, residual versus-fitted-values, boxplots of the residual for each DAP and normal probability plots of the residuals were obtained and are presented in Additional file 3: Figures S10–S15. The residual versus-fitted-values and residual boxplots appear to be satisfactory, there not being any evidence of heterogeneity of variance. However, the normal probability plots indicate that the normality assumption underlying the longitudinal analysis is not met. On the other hand, the shape made by the residuals in the plot indicates that the data are symmetrically distributed. It is concluded that the results of the analyses will be approximately correct, especially given the large number of observed values in each analysis.

## Supplementary information

**Additional file 1.** Supporting material for the analyses of the tomato experiment based on the smoothing and extraction of traits (SET). Three median deviations plots for choosing the smoothing DF and method for the tomato experiment. Three plots comparing direct and log smoothing for six smoothing DF. A table of *p*-values for determining the significance of the effect of Zn and AMF on the extracted per-cart traits. Residual-versus-fitted-values and normal probability plots for each of the per-cart traits.

**Additional file 2.** A comparison of the results from separate and joint analyses of per-cart traits. A description of the joint analysis of the per-cart traits. The results of the joint analysis. A comparison of the predictions and LSDs from the separate and joint analyses.

**Additional file 3.** Supporting material for the longitudinal analysis. Four plots comparing the predicted trends for four different knot numbers and four plots showing the deviations between unsmoothed and smoothed predictions. A table of Wald F-statistic *p*-values and a summary table of the variance model hypothesis testing for each of PSA and ln(PSA) for the full variance model. A table of Wald F-statistic *p*-values for each of PSA and ln(PSA) for the reduced variance model. Two plots comparing the predictions and standard errors obtained under the full and reduced variance models. Residual-versus-fitted-values, residual boxplots for different DAPS and normal probability plots for the longitudinal analysis of PSA and ln(PSA).

**Additional file 4.**R scripts and data for preparing the tomato data and carrying out the reported analyses. The data is provided in the file tomato.dat.csv, but in R is also available with the growthPheno package. The script global.r provides settings, constants and functions that are used across all scripts and is executed at the beginning of most scripts. The script SET.r gives the code for obtaining the smoothed longitudinal data (Steps 1–4 of the SET process). Cart.dat.r extracts the per-cart.traits (Step 5 of the SET process). Cart.anal.r analyses the per-cart data and Cart.predict.r obtains the predictions based on the selected models (Step 6 of the SET-based analysis). Cart.joint.r performs the extra joint analysis of per-cart traits. Longi.anal.r fits several models to all the tomato data for PSA and ln(PSA) in order to establish a variance model for each and then, for the selected variance model, the number of knots for the splines describing the curved trend for each combination of Zn and AMF is varied (Stages 1–2). Longi.predict.r obtains the predictions for the different numbers of knots (Stage 3). Longi.trend.r investigates the effect of Zn and AMF on the time trend when 10 knots are used and does diagnostic checking of the residuals (Stage 4); it also fits a reduced variance model that assumes equal variances for different DAPs and zero correlation between DAPs.

## Data Availability

The tomato data set is incorporated into the R package growthPheno [24], publicly available on CRAN. It has been provided in Additional file 4, as have the R scripts for preparing and analyzing the data as described in the paper.

## Abbreviations

AGR: Absolute growth rate
AMF: Arbuscular mycorrhizal fungi
DAP: Days after planting
DF: Degrees of freedom
FRM: Fixed regression model
FRMS: Fixed regression model with splines
GR: Growth rate
GWAS: Genome wide association study
HTP: High throughput phenotyping
LSD: Least significant difference
PSA: Projected shoot area
QTL: Quantitative trait loci
REMLRT: Restricted maximum likelihood ratio test
RGR: Relative growth rate
RRM: Random regression model
RRMS: Random regression model with splines
SET: Smoothing and extraction of traits
sPSA: Smoothed projected shoot area
TUE: Transpiration use efficiency
WUI: Water use index
