# Supplementary material for: Smoothing and extraction of traits in the growth analysis of noninvasive phenotypic data
Source: Plant Methods. 2020 Mar 10;16:36. doi: 10.1186/s13007-020-00577-6 (PMC7065360; doi:10.1186/s13007-020-00577-6)
Supplement: Supplementary file 1 — Additional file 1. Supporting material for the analyses of the tomato experiment based on the smoothing and extraction of traits (SET). Three median deviations plots for choosing the smoothing DF and method for the tomato experiment. Three plots comparing direct and log smoothing for six smoothing DF. A table of p-values for determining the significance of the effect of Zn and AMF on the extracted per-cart traits. Residual-versus-fitted-values and normal probability plots for each of the per-cart traits. [file 13007_2020_577_MOESM1_ESM.pdf]

Smoothing and extraction of traits in the growth analysis of noninvasive phenotypic data

by C. Brien, N. Jewell, S.J. Watt-Williams, T. Garnett and B. Berger.
https://dx.doi.org/10.1186/s13007-020-00577-6

Additional File 1: Additional material for the analyses of the tomato experiment based on the smoothing and extraction of traits (SET)

Plots of median deviations

In each of Figures S1–S3, each line corresponds to a smoothing method. Median values above zero indicate that the smoothed value is under-estimating the trend or that there is a positive transient effect; median values below zero indicate the opposite. The dashed envelopes provide a guide to the magnitude of the deviations, as a proportion of the median of the response plotted.

Plots comparing raw, directly smoothed and logarithmically smoothed profile plots for six smoothing DF

In each of Figures S4–S6, the left hand panels were obtained by direct smoothing of either the PSA, PSA AGR or PSA RGR values using six smoothing DF, whilst the right hand panels are based on logarithmic smoothing. The central panel is the raw, observed data. The dashed vertical lines divide the imaging period into intervals of reasonably homogeneous growth dynamics.

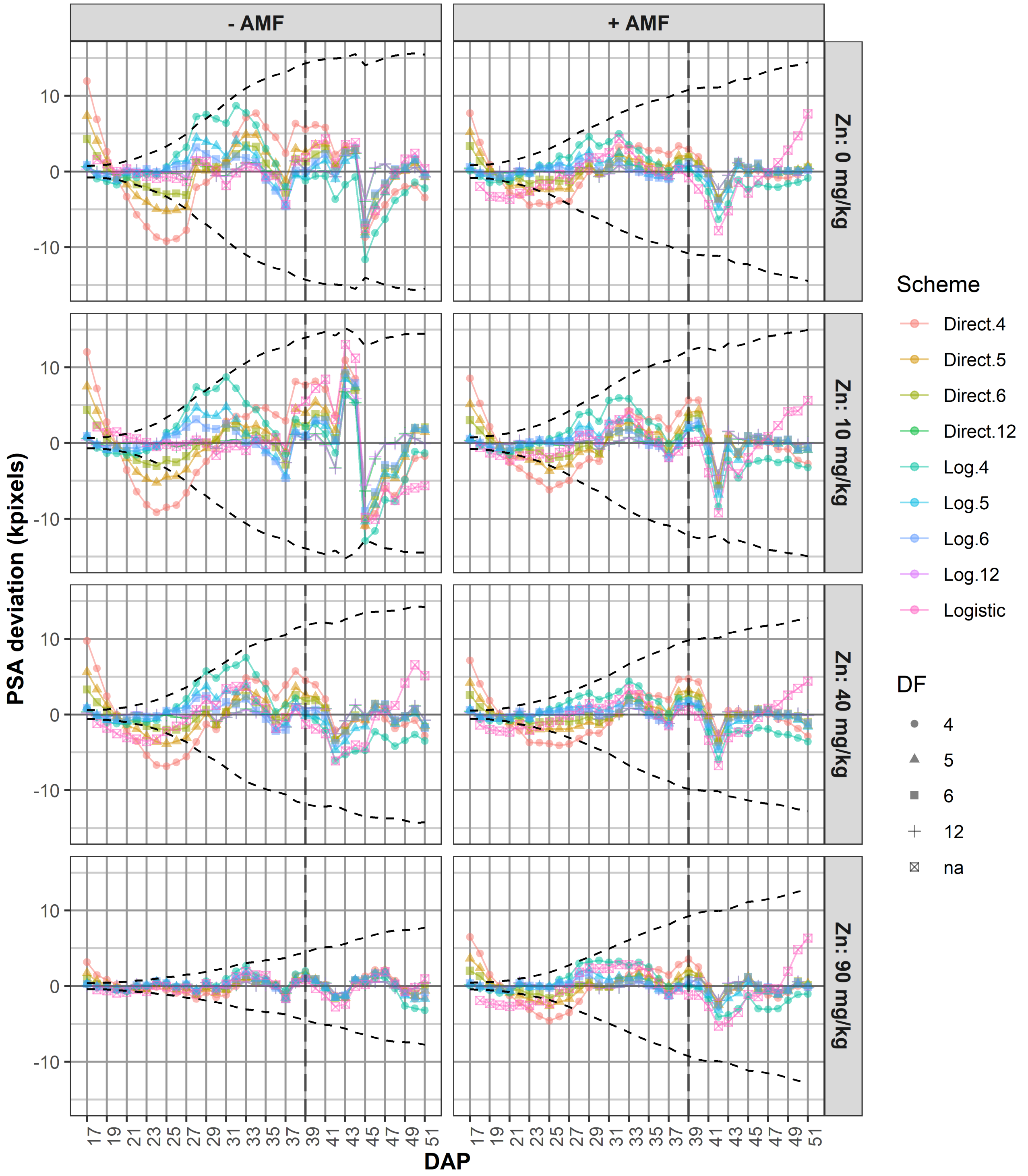

Figure S1: Plots of the median of deviations for PSA. The dashed envelopes are 0.10 times the raw median PSA. The colours differ with the Zn treatments.

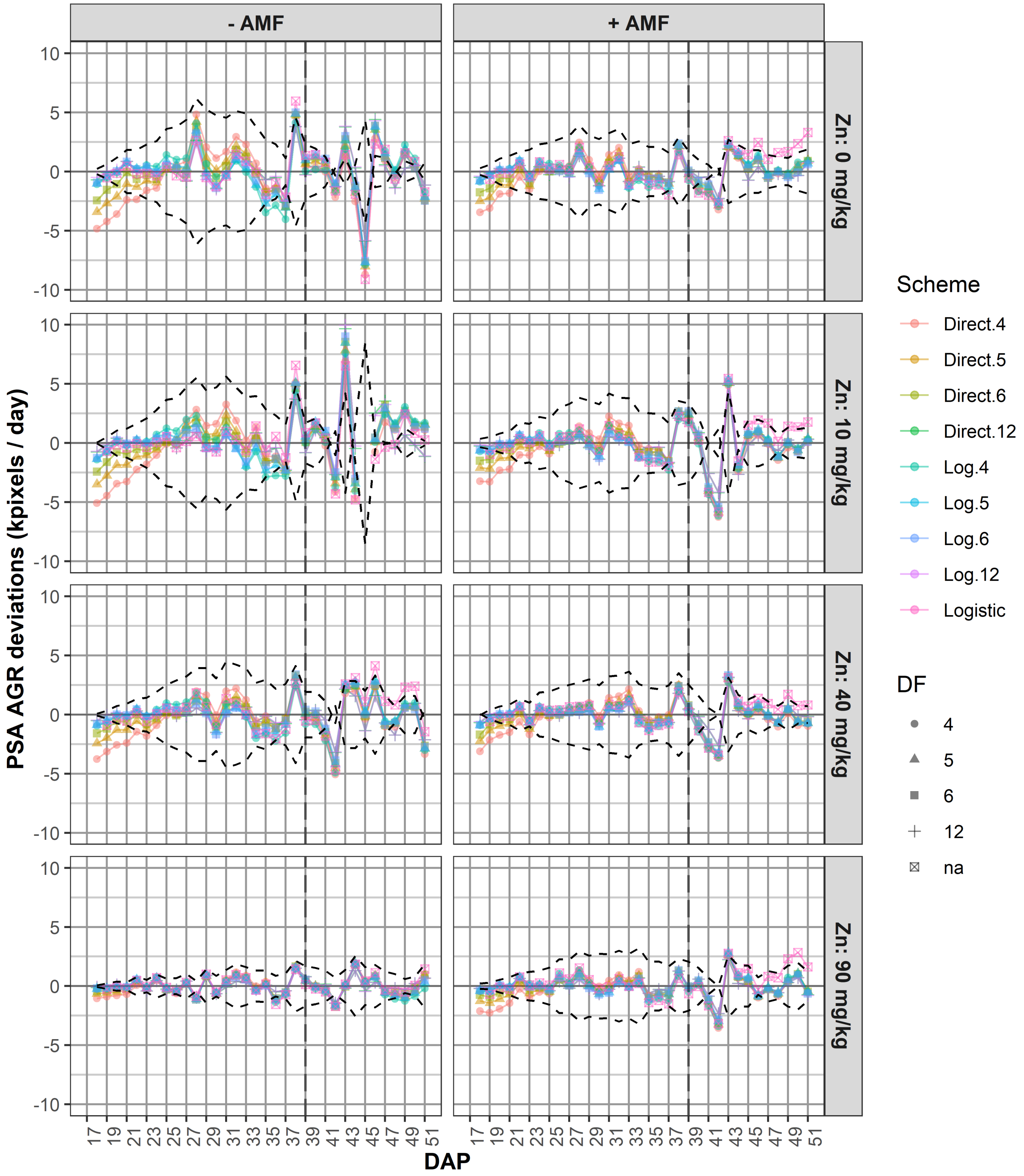

Figure S2: Plots of the median of deviations for PSA AGR. The dashed envelopes are 0.5 times the raw median PSA. The colours differ with the Zn treatments.

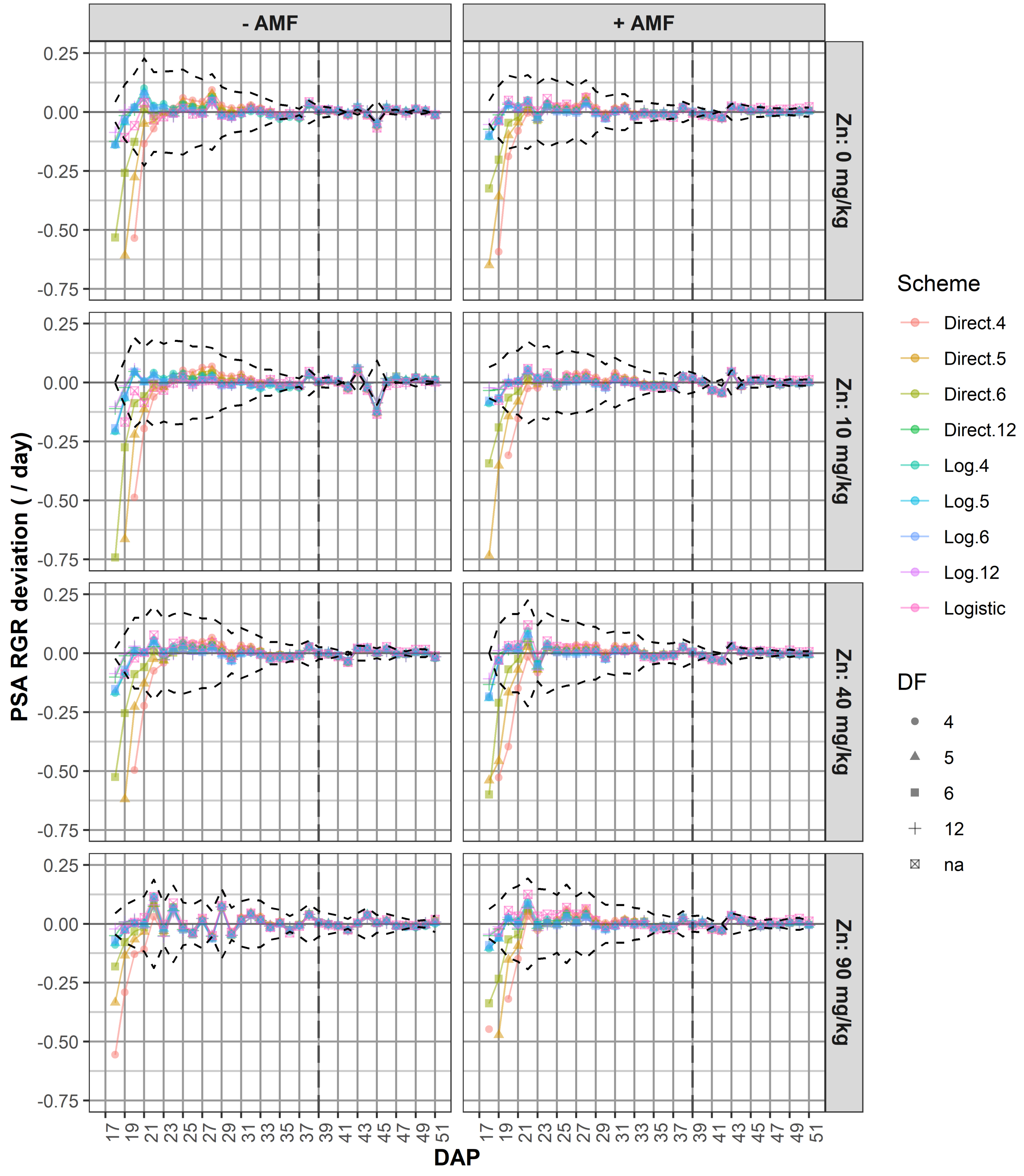

Figure S3: Plots of the median of deviations for PSA RGR. The dashed envelopes are 0.75 times the raw median PSA. The colours differ with the Zn treatments.

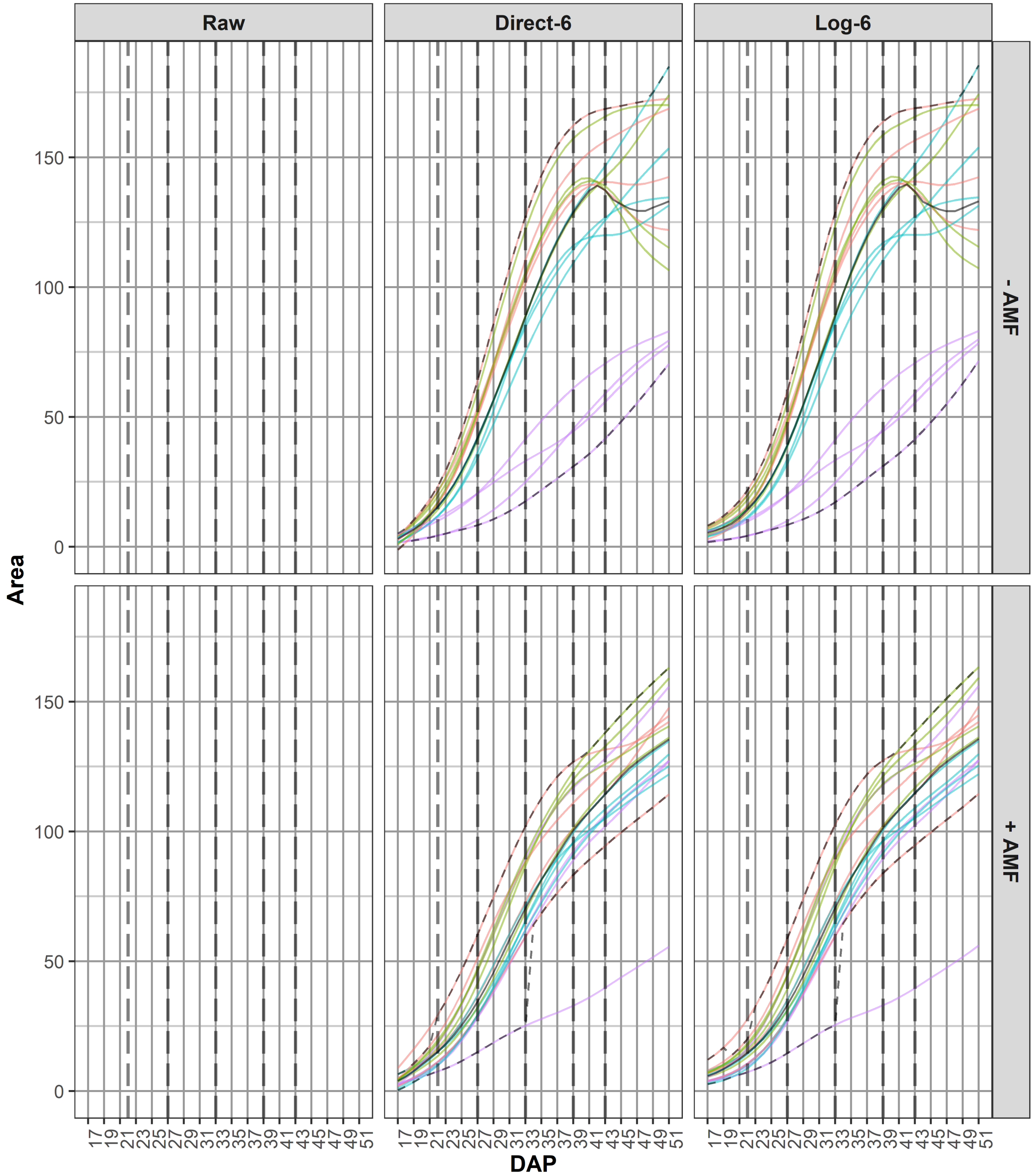

Figure S4: Profile plots of the directly smoothed, raw and log smoothed PSA for six smoothing DF. The black line is the median profile and the dashed lines are the outer whiskers (points outside the whiskers are potential outliers).

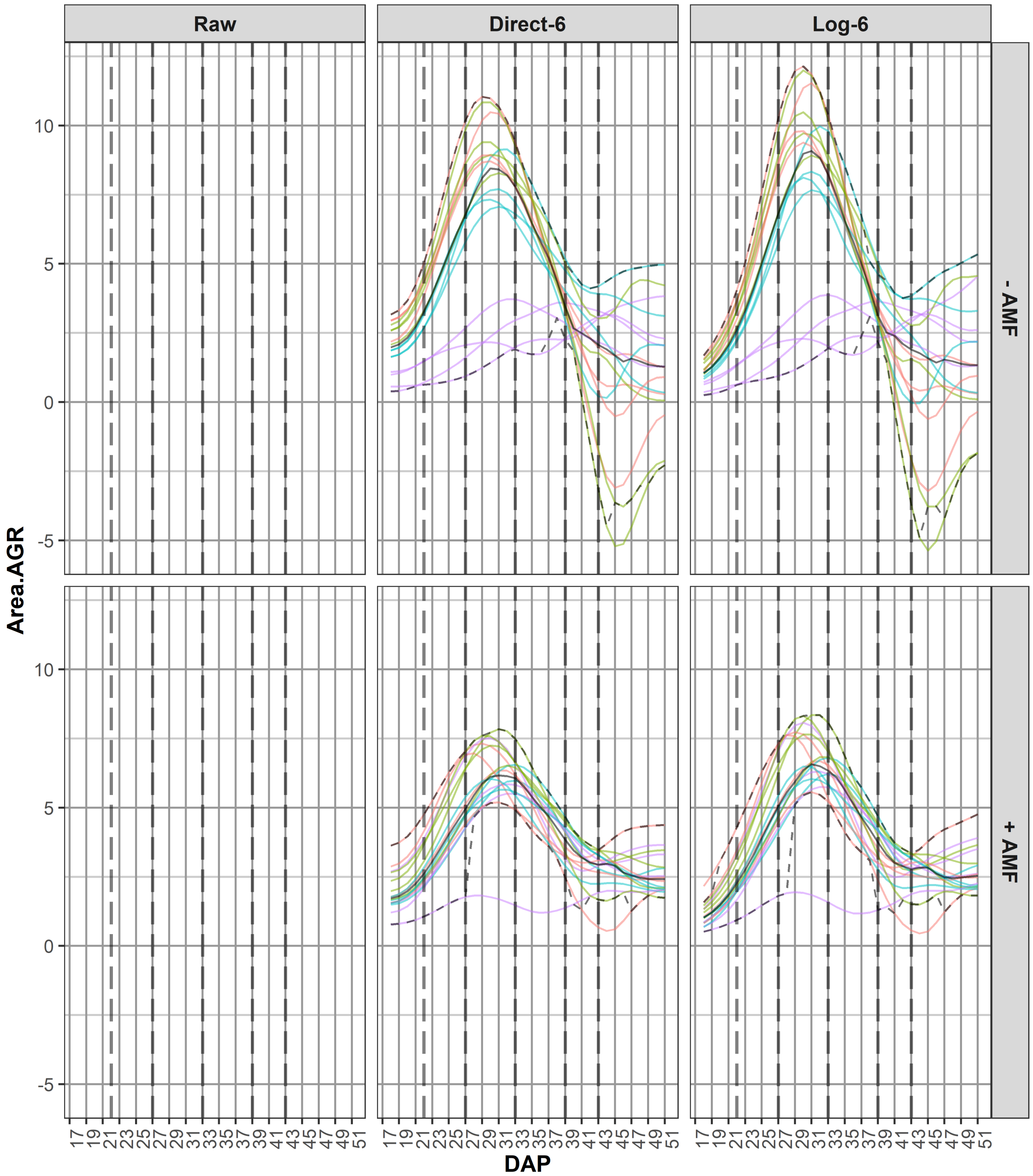

Figure S5: Profile plots of the directly smoothed, raw and log smoothed PSA AGR for six smoothing DF. The black line is the median profile and the dashed lines are the outer whiskers (points outside the whiskers are potential outliers).

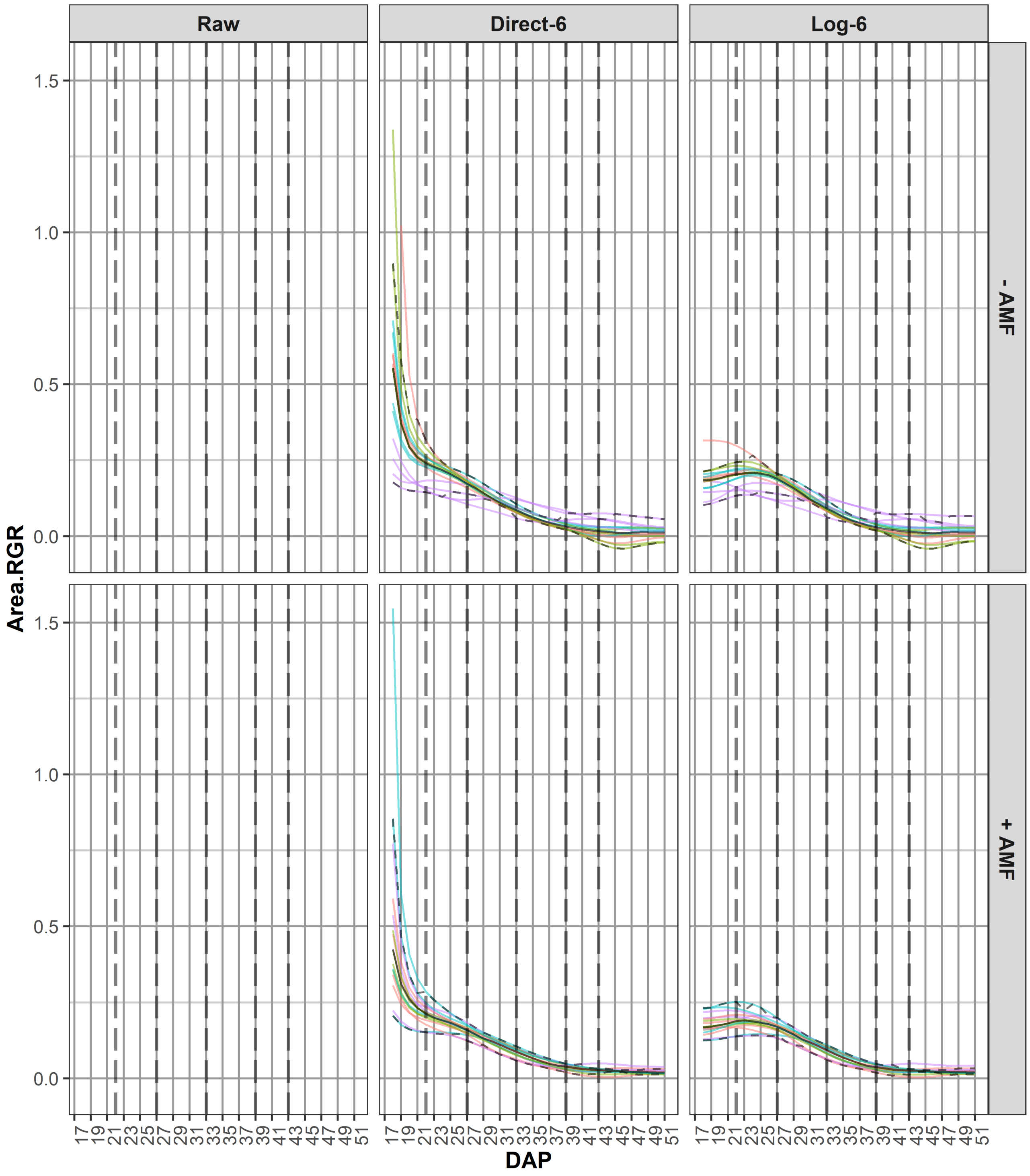

Figure S6: Profile plots of the directly smoothed, raw and log smoothed PSA RGR for six smoothing DF. The black line is the median profile and the dashed lines are the outer whiskers (points outside the whiskers are potential outliers).

Summary of the results of hypothesis tests for the per-cart traits

Table S1 gives the p-values for the Wald F-statistics that test for the interaction of Zn and AMF on the per-cart growth traits. Statistically significant terms ($p \leq 0.05$) are flagged with an asterisk (‘*‘). Where the interaction is significant, the main effect p-values are suppressed (not applicable, na). For example, for sPSA 27, the interaction is significant and so the p-values are not shown for the Zn and AMF main effects. It is concluded that both Zn and AMF affect the sPSA at DAP 27, but that the differences between the Zn levels depend on whether or nor AMF has been inoculated. On the other hand, for sPSA 18, the interaction is not significant; the p-values for the main effects are given and only Zn is significant. That is, there is no effect of AMF on sPSA at DAP 18.

Table S1: The p-values of the Wald F-statistics for the effects of Zn and AMF on per-cart traits

Trait	Zn:AMF	Zn	AMF
sPSA.18	0.883	* 0.017	0.344
sPSA.22	0.522	* <0.001	0.536
sPSA.27	* 0.008	na	na
sPSA.33	* <0.001	na	na
sPSA.39	* <0.001	na	na
sPSA.43	* <0.001	na	na
sPSA.51	* 0.004	na	na
sPSA.AGR.18to22	0.056	* <0.001	0.894
sPSA.AGR.22to27	* <0.001	na	na
sPSA.AGR.27to33	* <0.001	na	na
sPSA.AGR.33to39	* <0.001	na	na
sPSA.AGR.39to43	0.283	0.122	* 0.029
sPSA.AGR.43to51	0.143	0.157	* 0.023
sPSA.RGR.18to22	* 0.013	na	na
sPSA.RGR.22to27	* <0.001	na	na
sPSA.RGR.27to33	0.282	0.235	0.149
sPSA.RGR.33to39	0.107	* 0.003	0.751
sPSA.RGR.39to43	* 0.002	na	na
sPSA.RGR.43to51	* 0.021	na	na

Residual-versus-fitted-values and normal probability plots of the residuals

The plots in Figures S7–S25 are based on the standardized conditional residuals. For the residual-versus-fitted-values plots, the expected pattern is one of a similar vertical distributions of points across all values plotted on the x-axis. Points outside the maroon lines are potential outliers. For normal probability plots, it can be concluded that the data are consistent with being normally distributed if all points lie within the envelopes included in the plot.

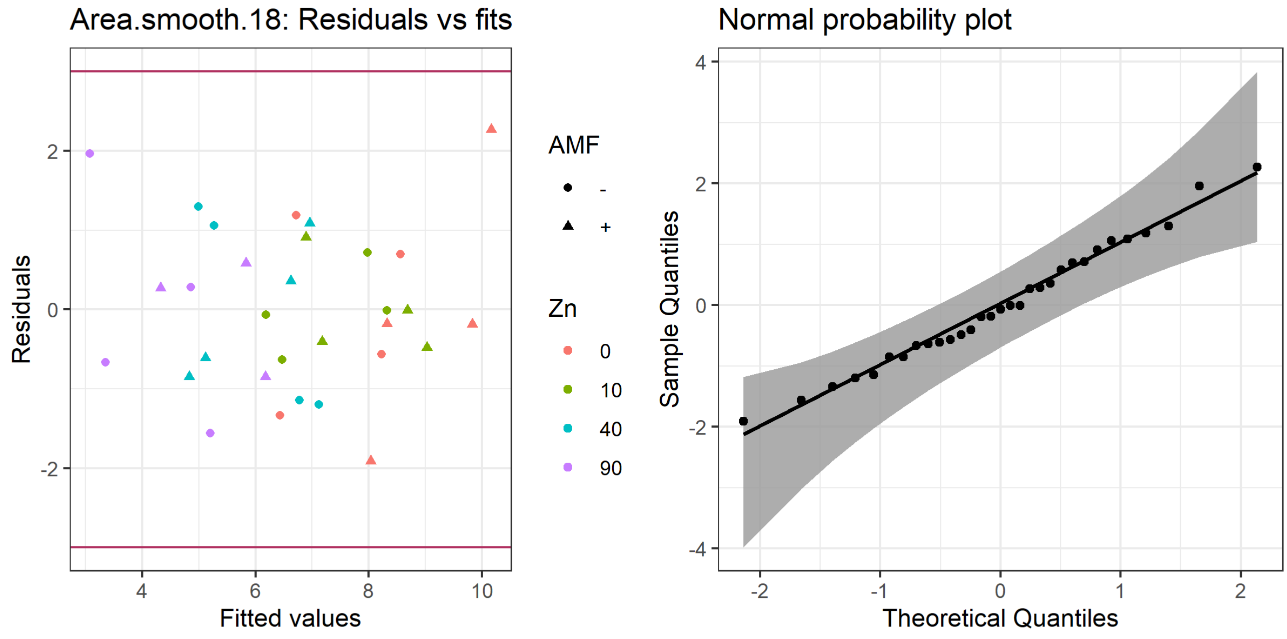

Figure S7: Residual plots for a per-cart trait.

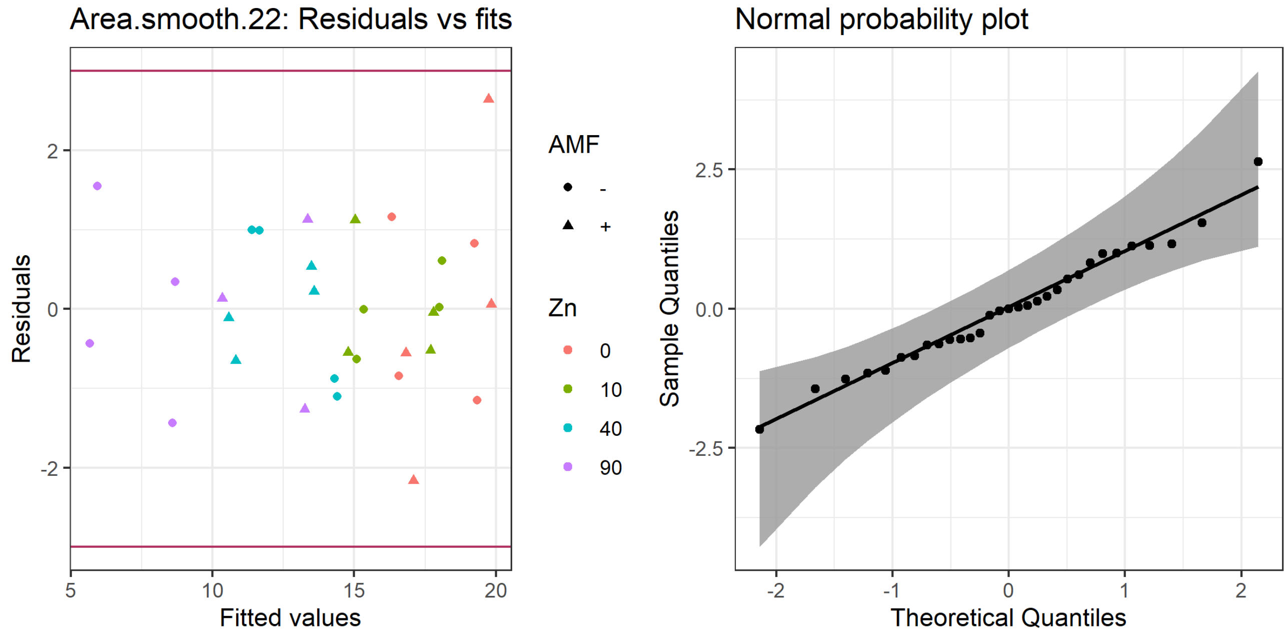

Figure S8: Residual plots for a per-cart trait.

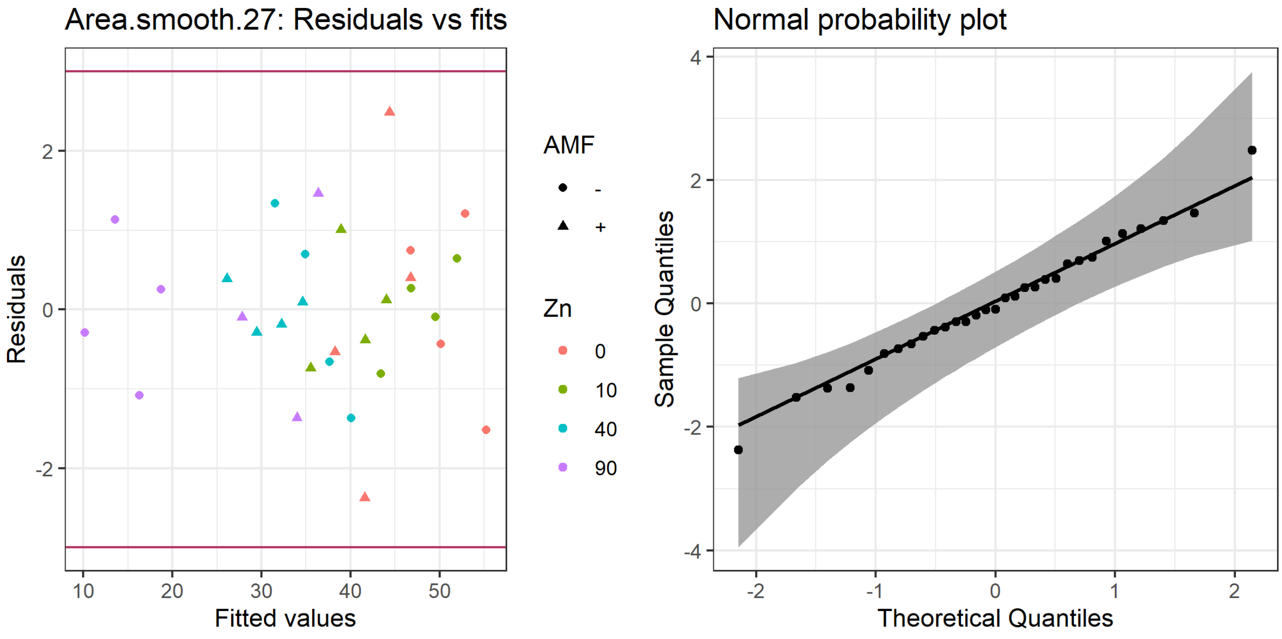

Figure S9: Residual plots for a per-cart trait.

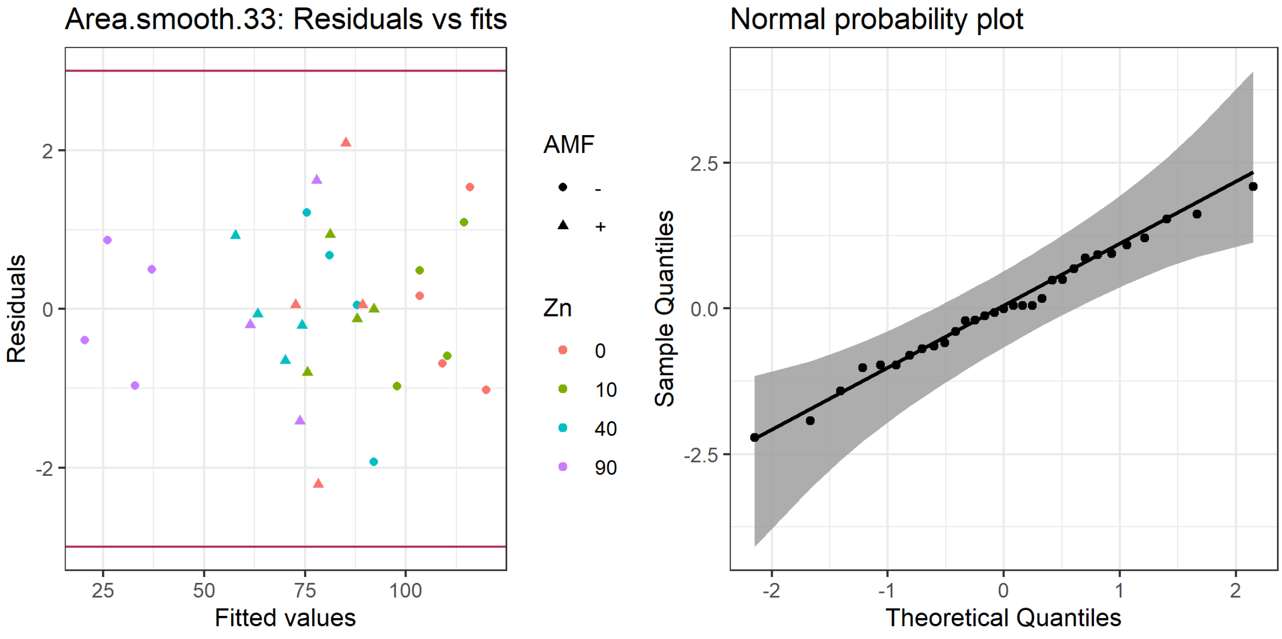

Figure S10: Residual plots for a per-cart trait.

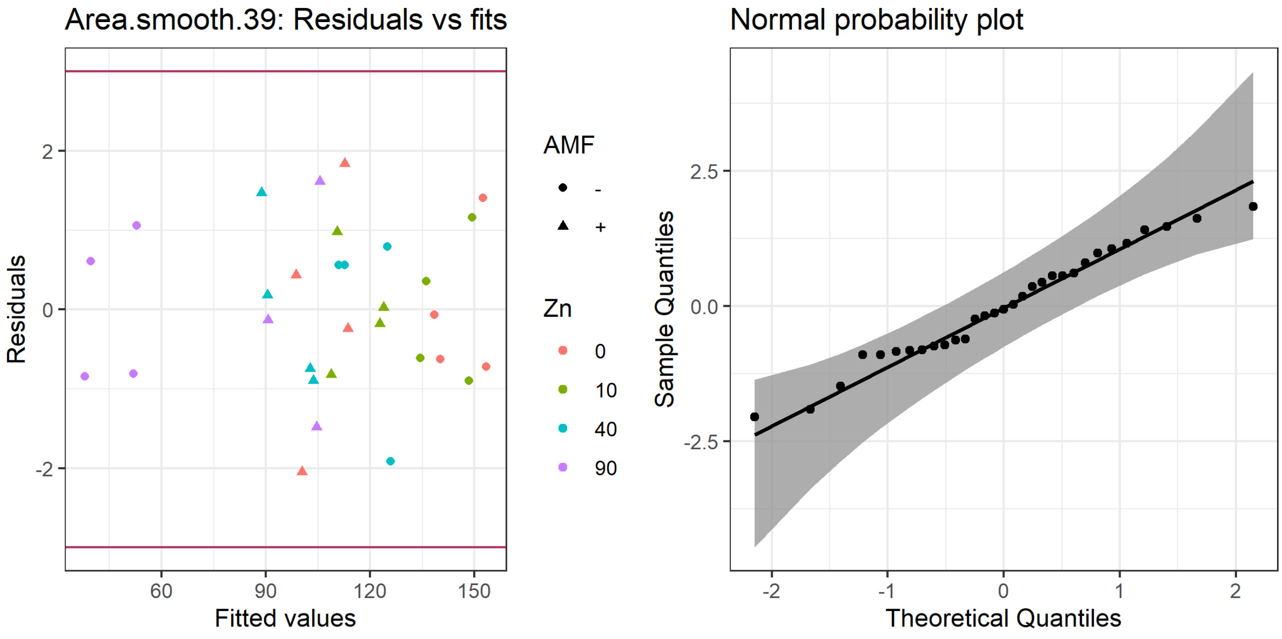

Figure S11: Residual plots for a per-cart trait.

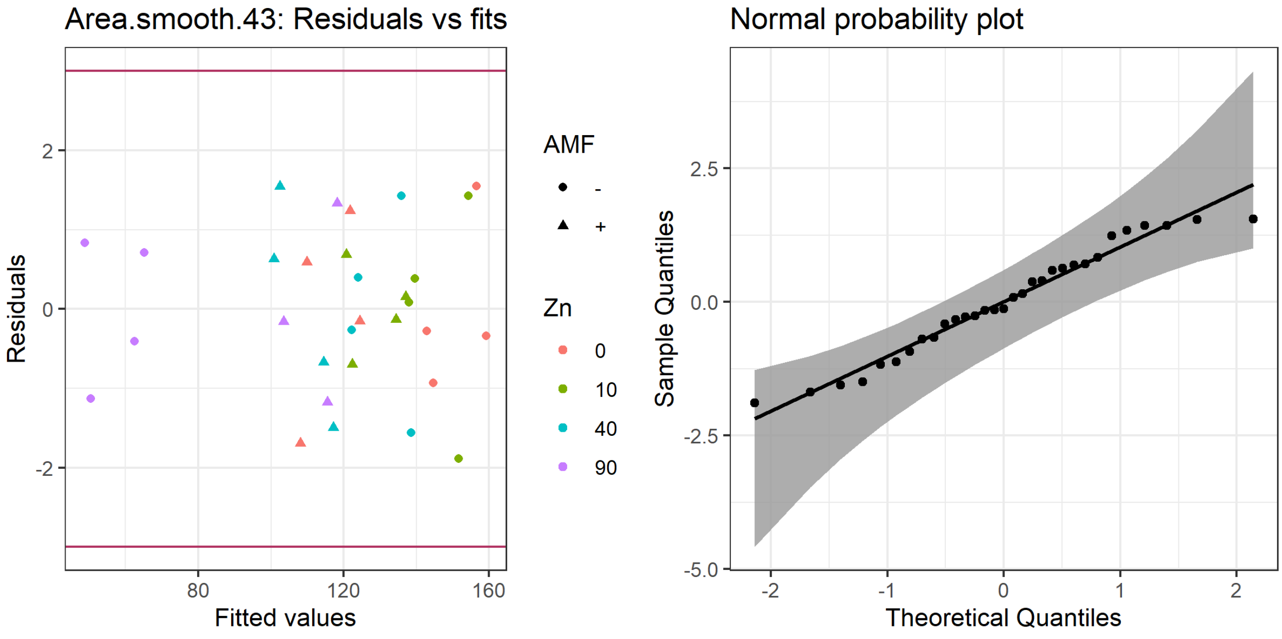

Figure S12: Residual plots for a per-cart trait.

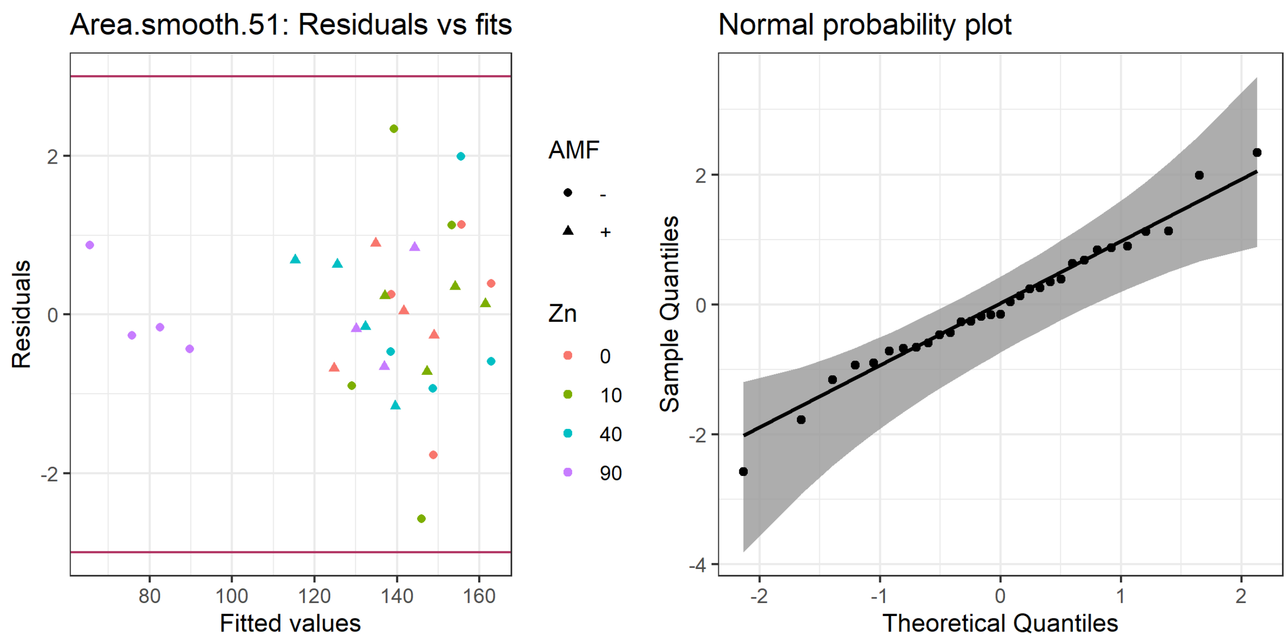

Figure S13: Residual plots for a per-cart trait.

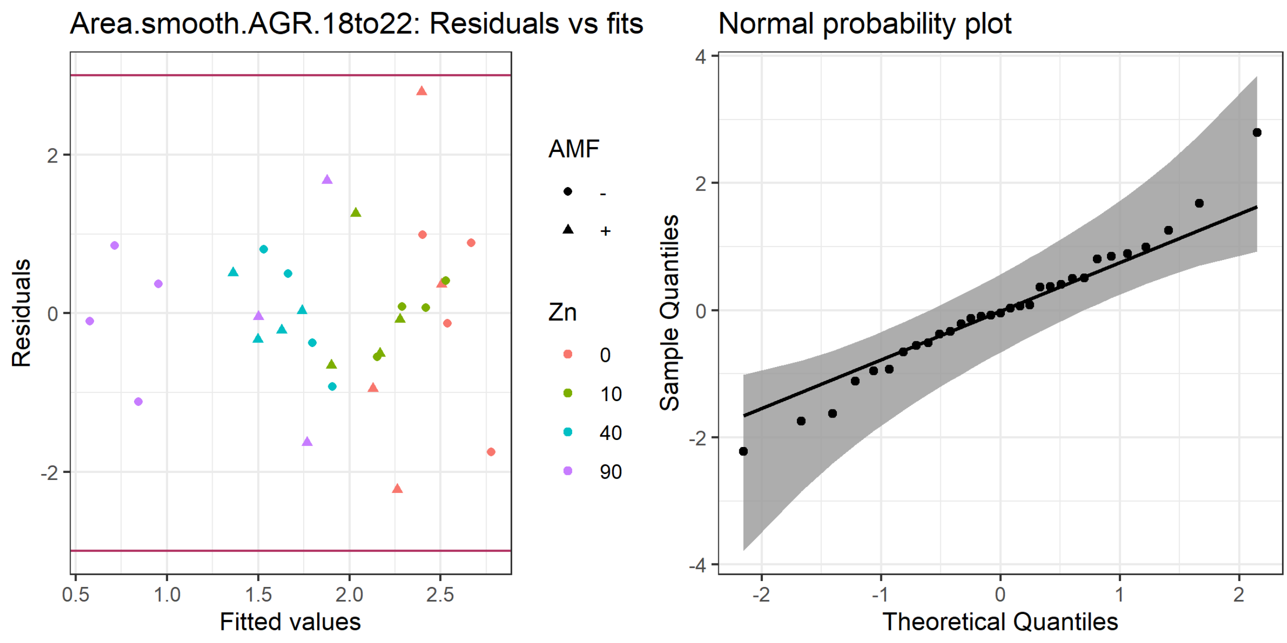

Figure S14: Residual plots for a per-cart trait.

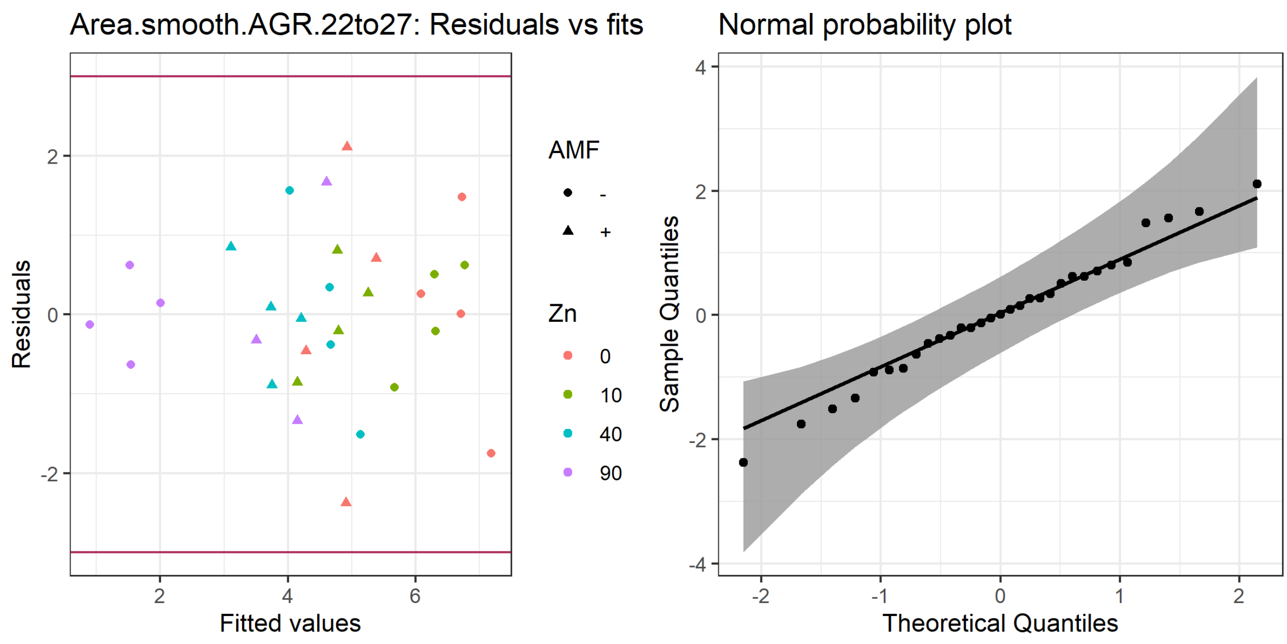

Figure S15: Residual plots for a per-cart trait.

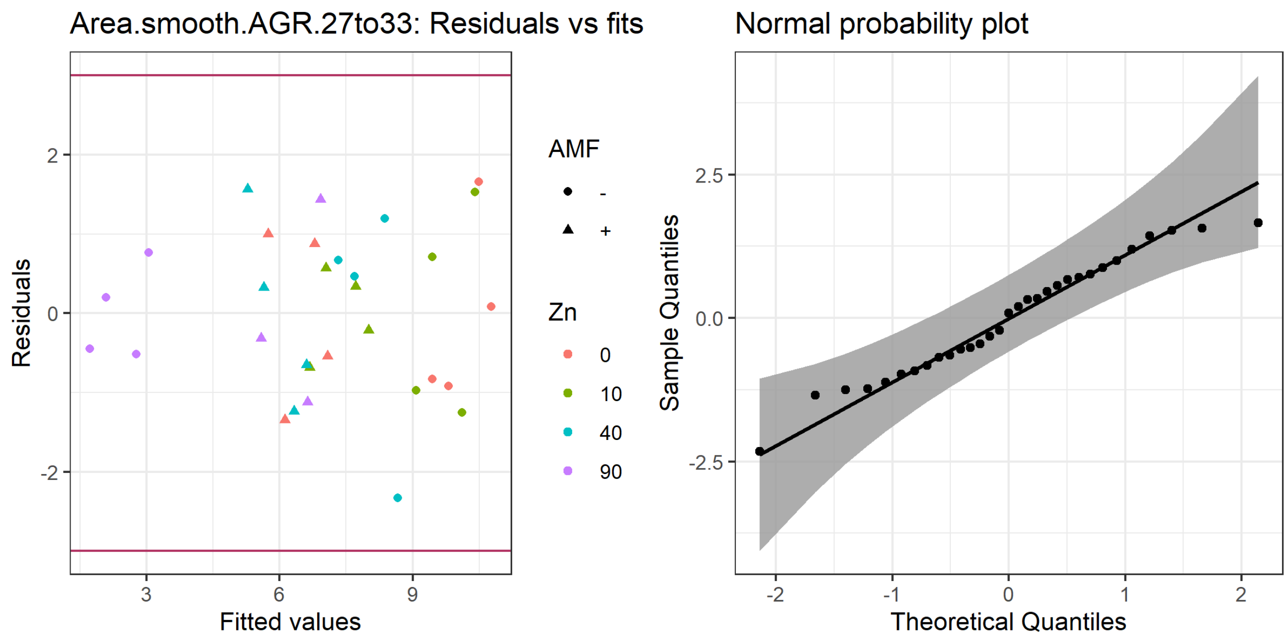

Figure S16: Residual plots for a per-cart trait.

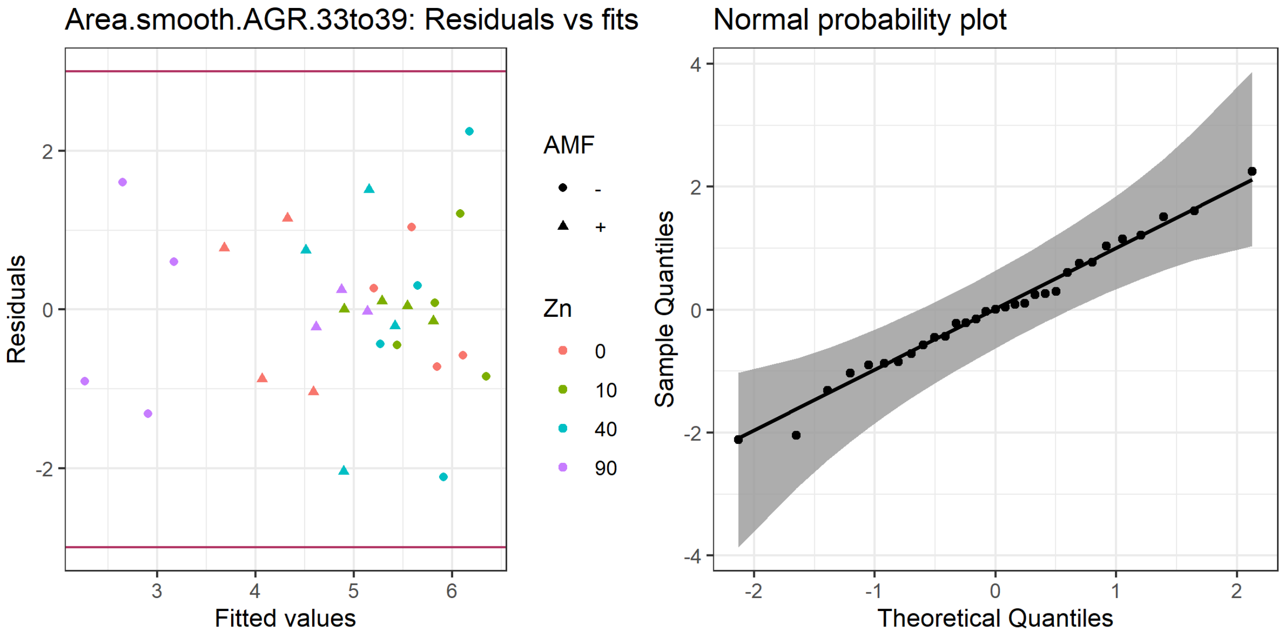

Figure S17: Residual plots for a per-cart trait.

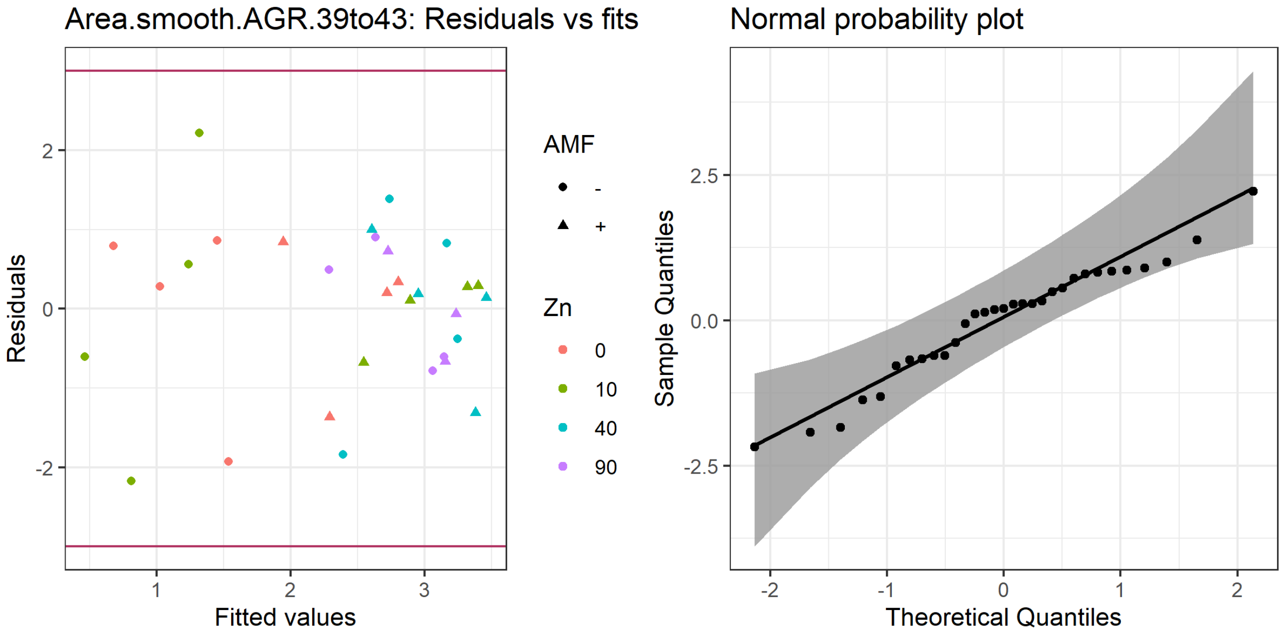

Figure S18: Residual plots for a per-cart trait.

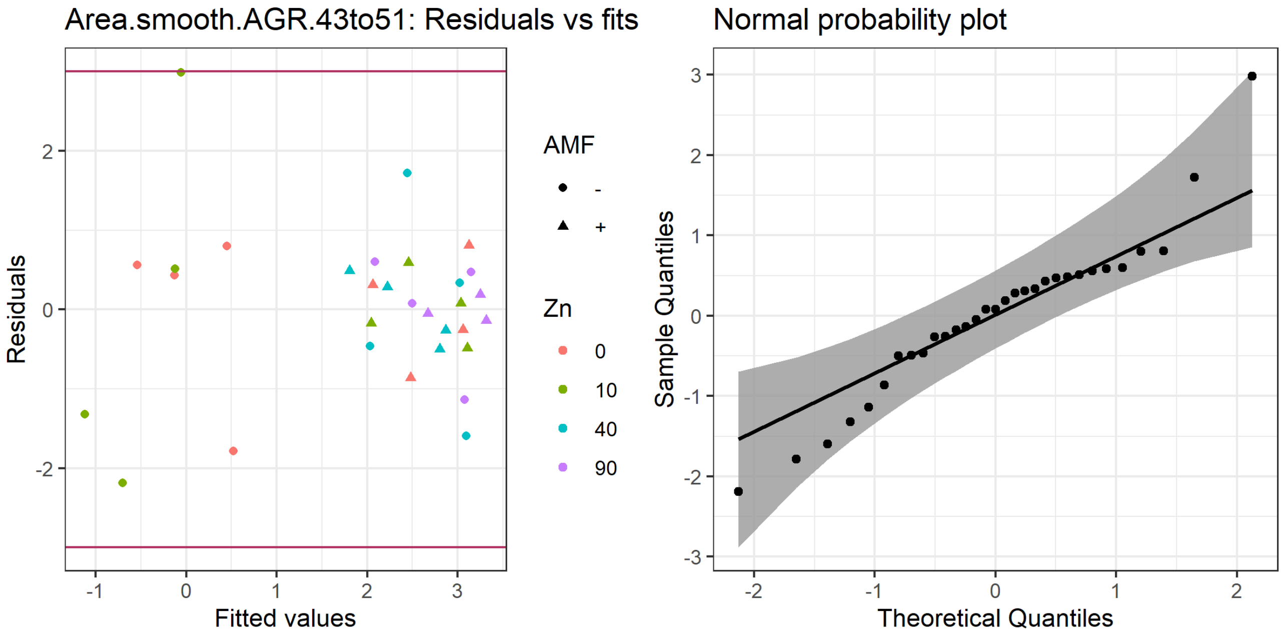

Figure S19: Residual plots for a per-cart trait.

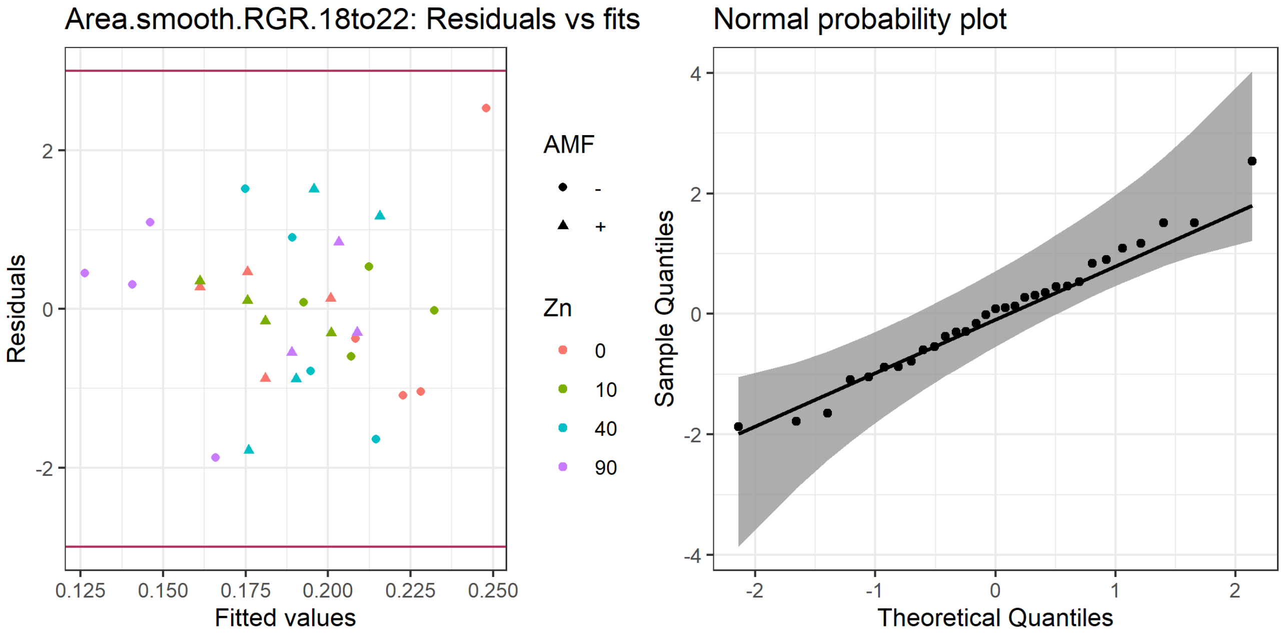

Figure S20: Residual plots for a per-cart trait.

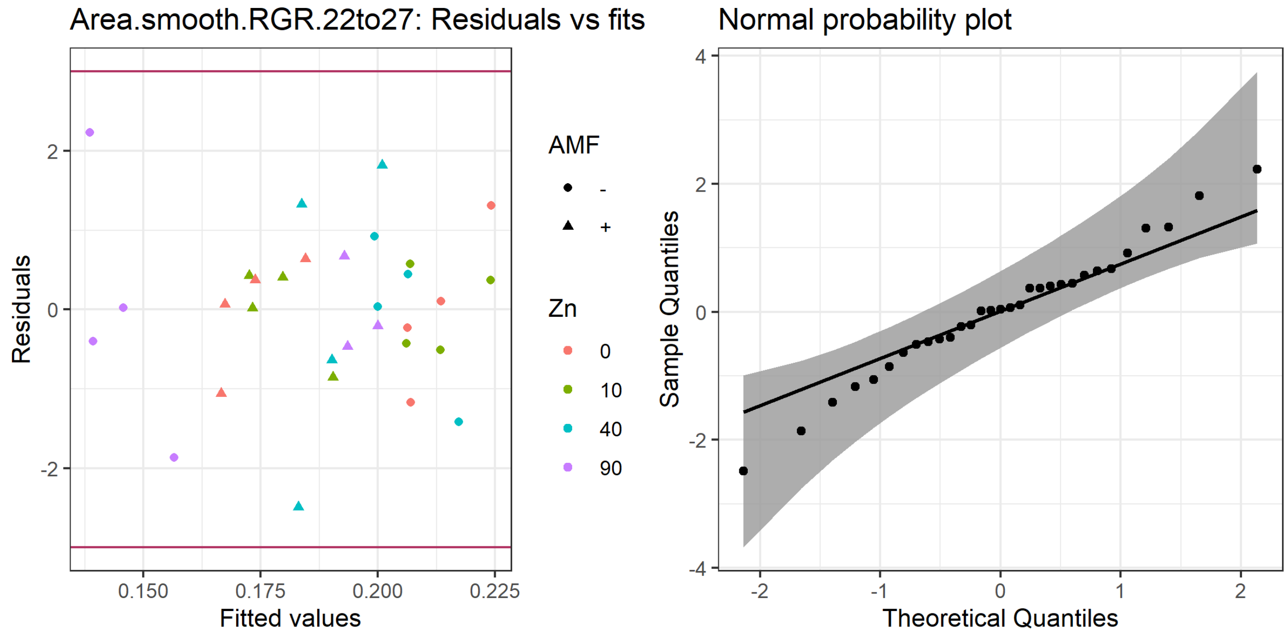

Figure S21: Residual plots for a per-cart trait.

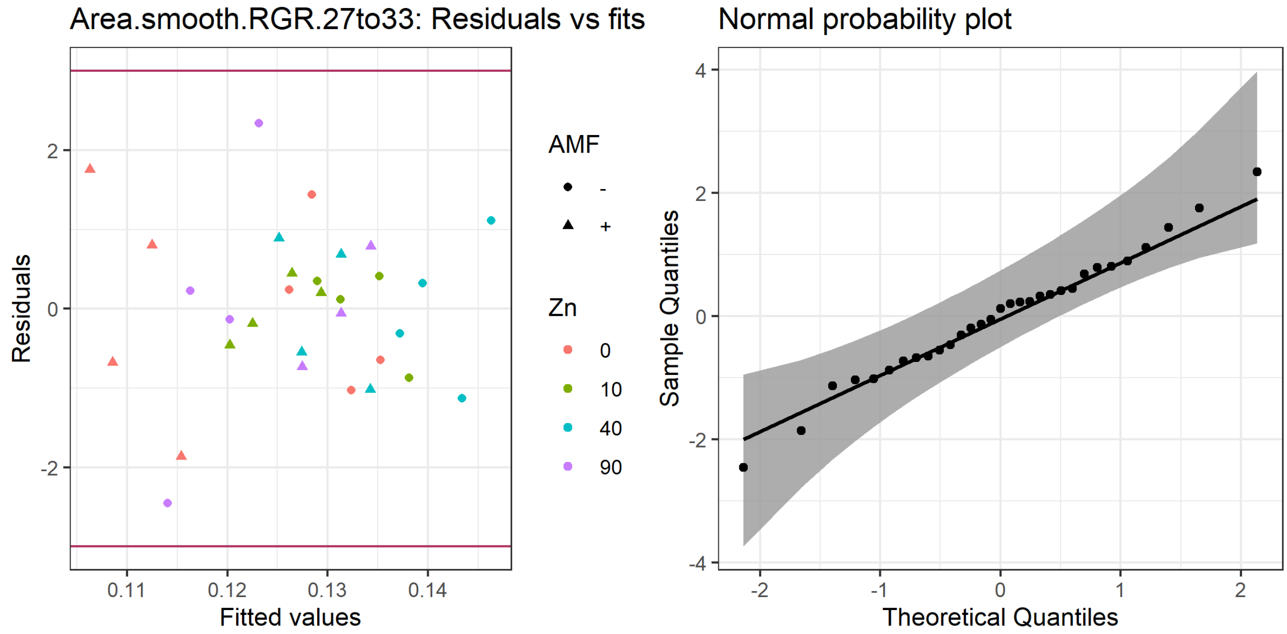

Figure S22: Residual plots for a per-cart trait.

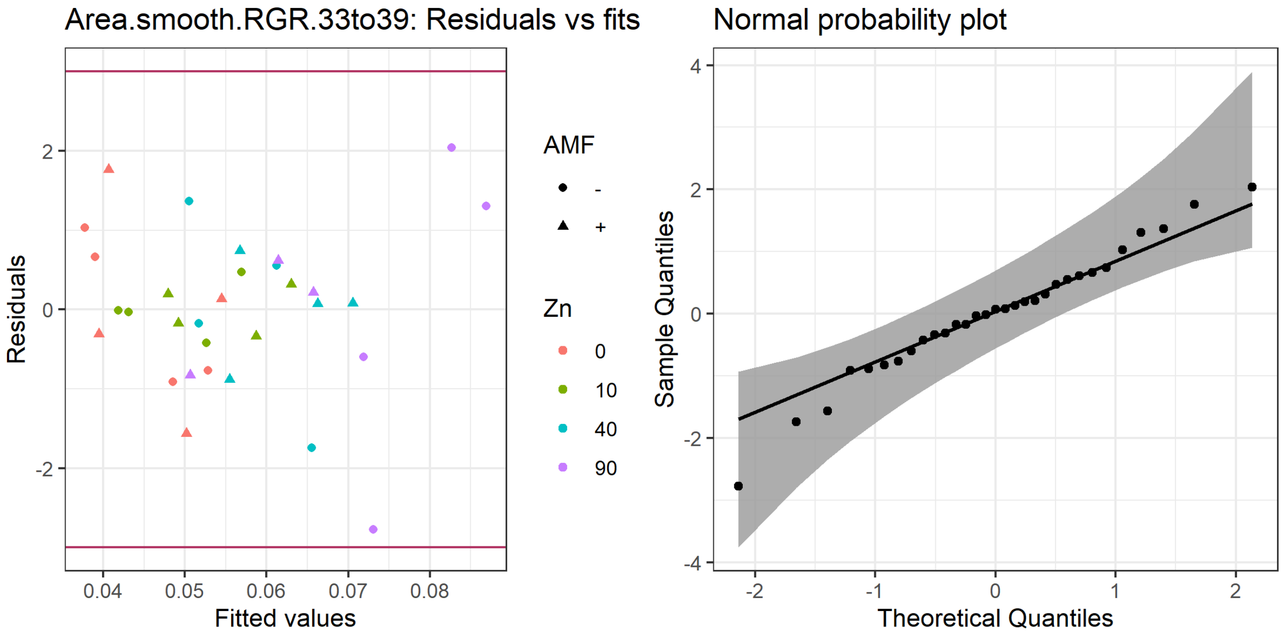

Figure S23: Residual plots for a per-cart trait.

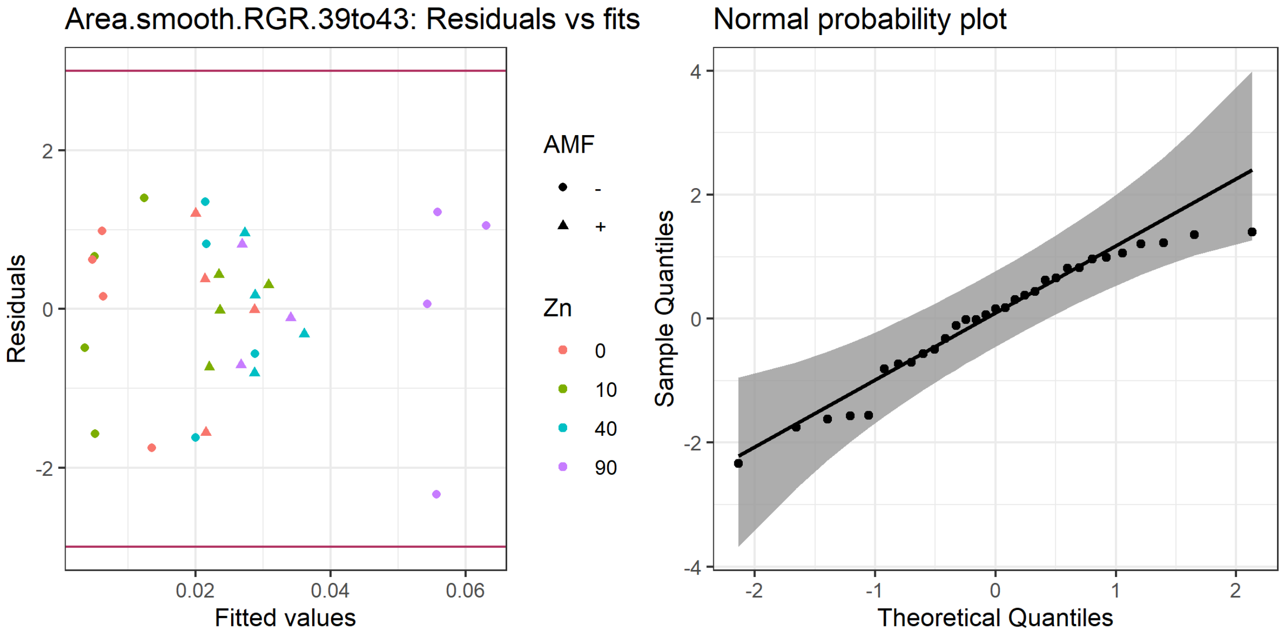

Figure S24: Residual plots for a per-cart trait.

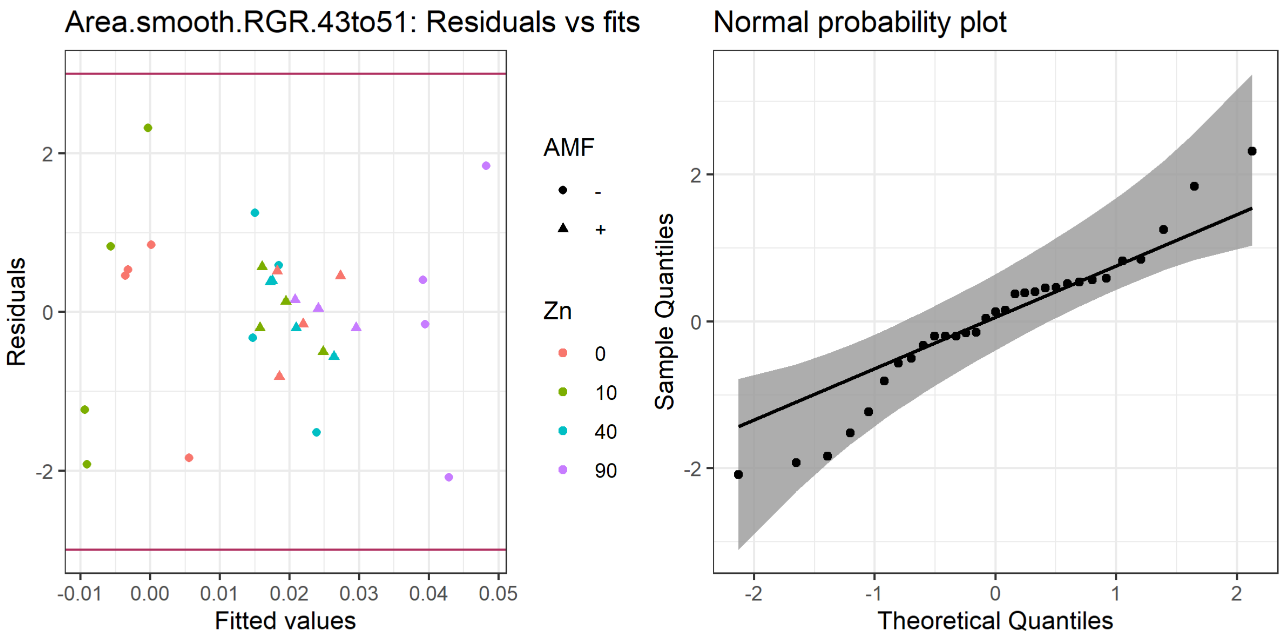

Figure S25: Residual plots for a per-cart trait.
