# Supplementary material for: Smoothing and extraction of traits in the growth analysis of noninvasive phenotypic data
Source: Plant Methods. 2020 Mar 10;16:36. doi: 10.1186/s13007-020-00577-6 (PMC7065360; doi:10.1186/s13007-020-00577-6)
Supplement: Supplementary file 2 — Additional file 2. A comparison of the results from separate and joint analyses of per-cart traits. A description of the joint analysis of the per-cart traits. The results of the joint analysis. A comparison of the predictions and LSDs from the separate and joint analyses. [file 13007_2020_577_MOESM2_ESM.pdf]

# Smoothing and extraction of traits in the growth analysis of noninvasive phenotypic data

by C. Brien, N. Jewell, S.J. Watt-Williams, T. Garnett and B. Berger.

https://dx.doi.org/10.1186/s13007-020-00577-6

# Additional File 2: A comparison of the results from separate and joint analyses of per-cart traits

## Joint analysis of the per-cart traits

In the proposed analysis of the per-cart traits, each trait is analysed separately by fitting a mixed model to it. However, such an analysis does not take into account the correlation between the measurements of the same trait at different times. To address this a joint analysis was performed, the object of which was to identify a model that described the correlations between the times.

### The mixed models for the analysis

The fixed-effects vector $\boldsymbol{\beta}$ is partitioned as $\boldsymbol{\beta}^\top = [\mu\ \boldsymbol{\beta}_\mathrm{B}^\top\ \boldsymbol{\beta}_\mathrm{Z}^\top\ \boldsymbol{\beta}_\mathrm{A}^\top\ \boldsymbol{\beta}_\mathrm{ZA}^\top\ \boldsymbol{\beta}_\mathrm{T}^\top\ \boldsymbol{\beta}_\mathrm{TB}^\top\ \boldsymbol{\beta}_\mathrm{TZ}^\top\ \boldsymbol{\beta}_\mathrm{TA}^\top\ \boldsymbol{\beta}_\mathrm{TZA}^\top]$, where (i) $\mu$ is the overall mean parameter, (ii) $\boldsymbol{\beta}_\mathrm{Z}$, $\boldsymbol{\beta}_\mathrm{A}$ and $\boldsymbol{\beta}_\mathrm{ZA}$ are the Zn main effects, AMF main effects and Zn-AMF interactions effects, (iii) $\boldsymbol{\beta}_\mathrm{T}$ is the Time main effects (either DAP of observation or DAP interval midpoint), and (iv) $\boldsymbol{\beta}_\mathrm{TZ}$, $\boldsymbol{\beta}_\mathrm{TA}$ and $\boldsymbol{\beta}_\mathrm{TZA}$ are the Time interactions with Zn and AMF.

The random-effects vector $\mathbf{u}$ is equal to $\mathbf{u}_\mathrm{BC}$, a vector of Block-Cart random effects.

The residual effects $\mathbf{e}$ are assumed to be normally distributed with mean vector $\mathbf{0}$ and variance matrix $\boldsymbol{\Sigma}_{32t}$, where $t$ is the number of times per-cart traits were extracted for the primary response being analyzed. For PSA, sPSA was extracted for seven DAPs; for PSA AGR and PSA RGR, six time intervals were calculated for each of sPSA AGR and sPSA AGR. A factor Time will be used to index these different times of observation, with the time of observation of the GRs taken as being for the mid-point of the time intervals i.e. the mean of the two endpoints of each time interval. For $\mathbf{y}$ ordered for Block-Cart then Time, $\boldsymbol{\Sigma}_{32t} = \mathbf{J}_{32} \bigotimes \boldsymbol{\Sigma}_\mathrm{T}$, where $\mathbf{J}_{32}$ is a $32 \times 32$ matrix of ones and $\boldsymbol{\Sigma}_\mathrm{T}$ is a $t \times t$ times matrix specifying the variances and covariances between the times. This implies that $\boldsymbol{\Sigma}_{32t}$ is split into $32 \times 32$ blocks each of which consists of $\boldsymbol{\Sigma}_\mathrm{T}$. The general form of $\boldsymbol{\Sigma}_\mathrm{T}$ is:

$$\boldsymbol{\Sigma}_\mathrm{T} = \begin{bmatrix} \sigma_1 & 0 & \cdots & 0 \\ 0 & \sigma_2 & \cdots & 0 \\ \vdots & \vdots & \ddots & \vdots \\ 0 & 0 & \cdots & \sigma_t \end{bmatrix} \begin{bmatrix} 1 & \rho_{12} & \cdots & \rho_{1t} \\ \rho_{21} & 1 & \cdots & \rho_{2t} \\ \vdots & \vdots & \ddots & \vdots \\ \rho_{t1} & \rho_{t2} & \cdots & 1 \end{bmatrix} \begin{bmatrix} \sigma_1 & 0 & \cdots & 0 \\ 0 & \sigma_2 & \cdots & 0 \\ \vdots & \vdots & \ddots & \vdots \\ 0 & 0 & \cdots & \sigma_t \end{bmatrix}$$

where $\sigma_i$ is the variance for the $i$th time and $\rho_{ij}$ is the correlation between the $i$th and $j$th times. Four different models are considered for $\boldsymbol{\Sigma}_\mathrm{T}$:

**Heterogeneous, independent times (idh):** all $\sigma_i$s are allowed to differ, $\rho_{ij} = 0$.

**Homogeneous, power correlation for times (exp):** $\sigma_i = \sigma$ and $\rho_{ij} = \rho^{|i-j|}$.

**Heterogeneous, power correlation for times (exph):** all $\sigma_i$s are allowed to differ, $\rho_{ij} = \rho^{|i-j|}$.

**Unstructured (us):** all $\sigma_i$s are allowed to differ, as are all $\rho_{ij}$s.

All four forms of $\boldsymbol{\Sigma}_\mathrm{T}$ were fitted to the data and REMLRT tests conducted to compare models. Firstly, us and exph models were compared and if significantly different, the us model was selected as required to describe the data. If not significant, exph was compared with both idh and exp models. If both are significant, then the exph models was selected; if only one if idh and exp is significant, then the significant model is selected.

## Results of the analysis

For all three primary responses, the unstructured model was selected because it was significantly different ($p < 0.001$) from the heterogeneous power correlation model. The estimated values of $\mathbf{\Sigma}_{\mathrm{T}}$ are given in Tables S1–S3.

**Table S1: Estimated $\mathbf{\Sigma}_{\mathrm{T}}$ for sPSA**

| | | | | | | |
|---|---|---|---|---|---|---|
| 1.000 | 0.958 | 0.851 | 0.743 | 0.596 | 0.339 | -0.093 |
| 0.958 | 1.000 | 0.955 | 0.851 | 0.714 | 0.449 | -0.045 |
| 0.851 | 0.955 | 1.000 | 0.940 | 0.815 | 0.575 | 0.028 |
| 0.743 | 0.851 | 0.940 | 1.000 | 0.948 | 0.768 | 0.222 |
| 0.596 | 0.714 | 0.815 | 0.948 | 1.000 | 0.900 | 0.407 |
| 0.339 | 0.449 | 0.575 | 0.768 | 0.900 | 1.000 | 0.723 |
| -0.093 | -0.045 | 0.028 | 0.222 | 0.407 | 0.723 | 1.000 |

**Table S2: Estimated $\mathbf{\Sigma}_{\mathrm{T}}$ for sPSA AGR**

| | | | | | |
|---|---|---|---|---|---|
| 1.000 | 0.921 | 0.499 | -0.435 | -0.561 | -0.368 |
| 0.921 | 1.000 | 0.672 | -0.428 | -0.450 | -0.345 |
| 0.499 | 0.672 | 1.000 | 0.142 | -0.089 | 0.022 |
| -0.435 | -0.428 | 0.142 | 1.000 | 0.580 | 0.515 |
| -0.561 | -0.450 | -0.089 | 0.580 | 1.000 | 0.816 |
| -0.368 | -0.345 | 0.022 | 0.515 | 0.816 | 1.000 |

**Table S3: Estimated $\mathbf{\Sigma}_{\mathrm{T}}$ for sPSA RGR**

| | | | | | |
|---|---|---|---|---|---|
| 1.000 | 0.725 | 0.377 | 0.424 | 0.298 | 0.198 |
| 0.725 | 1.000 | 0.757 | 0.586 | 0.490 | 0.156 |
| 0.377 | 0.757 | 1.000 | 0.860 | 0.545 | 0.297 |
| 0.424 | 0.586 | 0.860 | 1.000 | 0.644 | 0.421 |
| 0.298 | 0.490 | 0.545 | 0.644 | 1.000 | 0.798 |
| 0.198 | 0.156 | 0.297 | 0.421 | 0.798 | 1.000 |

They demonstrate that the assumption of equal correlation for times the same distance apart is not tenable.

# Comparison of results from separate and joint analyses

Firstly, we compare, in Tables S4–S6, the means of the LSDs calculated from the separate and joint analyses for comparing the difference between Zn-AMF combinations with a time. The LSD values for one time are not all in equal, because the removal of a plant means that the treatments are not equally replicated; however, the differences are small. The differences between the two analyses are less than 2%.

**Table S4: Mean LSDs from separate and joint analyses for sPSA**

| | Separate analyses | Joint analysis | Difference (%) |
|---|---|---|---|
| Area.smooth.18 | 3.3 | 3.3 | -1.19 |
| Area.smooth.22 | 5.7 | 5.6 | -1.19 |
| Area.smooth.27 | 10.9 | 10.8 | -1.19 |
| Area.smooth.33 | 15.7 | 15.5 | -1.19 |
| Area.smooth.39 | 15.1 | 14.9 | -1.19 |
| Area.smooth.43 | 14.9 | 14.8 | -1.19 |
| Area.smooth.51 | 28.1 | 27.8 | -1.19 |

**Table S5: Mean LSDs from separate and joint analyses for sPSA AGR**

| | Separate analyses | Joint analysis | Difference (%) |
|---|---|---|---|
| Area.smooth.AGR.18to22 | 0.67 | 0.66 | -1.36 |
| Area.smooth.AGR.22to27 | 1.15 | 1.13 | -1.36 |
| Area.smooth.AGR.27to33 | 1.10 | 1.09 | -1.36 |
| Area.smooth.AGR.33to39 | 0.84 | 0.83 | -1.36 |
| Area.smooth.AGR.39to43 | 1.68 | 1.66 | -1.36 |
| Area.smooth.AGR.43to51 | 2.52 | 2.48 | -1.36 |

**Table S6: Mean LSDs from separate and joint analyses for sPSA RGR**

| | Separate analyses | Joint analysis | Difference (%) |
|---|---|---|---|
| Area.smooth.RGR.18to22 | 0.046 | 0.045 | -1.36 |
| Area.smooth.RGR.22to27 | 0.029 | 0.028 | -1.36 |
| Area.smooth.RGR.27to33 | 0.023 | 0.022 | -1.36 |
| Area.smooth.RGR.33to39 | 0.017 | 0.017 | -1.36 |
| Area.smooth.RGR.39to43 | 0.016 | 0.016 | -1.36 |
| Area.smooth.RGR.43to51 | 0.020 | 0.020 | -1.36 |

Secondly, we compare the predictions and LSDs obtained from the separate and joint analyses in Tables S7–S9. The LSDs are the mean LSD for a time. There is very little difference between the values obtained from the two analyses.

**Table S7: Comparison of predictions and mean LSDs for sPSA**

| | | | Separate analyses | | Joint analyses | |
|---|---|---|---|---|---|---|
| DAP | AMF | Zn | Prediction | LSD (5%) | Prediction | LSD (5%) |
| 18 | - | 0 | 7.48349 | 3.3258 | 7.48349 | 3.2864 |
| 18 | - | 10 | 7.23739 | 3.3258 | 7.23739 | 3.2864 |
| 18 | - | 40 | 6.04026 | 3.3258 | 6.04026 | 3.2864 |
| 18 | - | 90 | 4.12758 | 3.3258 | 4.12758 | 3.2864 |
| 18 | + | 0 | 9.08390 | 3.3258 | 9.08390 | 3.2864 |
| 18 | + | 10 | 7.94306 | 3.3258 | 7.94306 | 3.2864 |
| 18 | + | 40 | 5.88241 | 3.3258 | 5.88241 | 3.2864 |
| 18 | + | 90 | 5.09575 | 3.3258 | 5.09575 | 3.2864 |
| 22 | - | 0 | 17.86788 | 5.6949 | 17.86788 | 5.6274 |
| 22 | - | 10 | 16.62808 | 5.6949 | 16.62808 | 5.6274 |
| 22 | - | 40 | 12.93739 | 5.6949 | 12.93739 | 5.6274 |
| 22 | - | 90 | 7.21250 | 5.6949 | 7.21250 | 5.6274 |
| 22 | + | 0 | 18.37357 | 5.6949 | 18.37357 | 5.6274 |
| 22 | + | 10 | 16.32219 | 5.6949 | 16.32219 | 5.6274 |
| 22 | + | 40 | 12.11094 | 5.6949 | 12.11094 | 5.6274 |
| 22 | + | 90 | 11.88015 | 5.6949 | 11.88015 | 5.6274 |
| 27 | - | 0 | 51.24286 | 10.9155 | 51.24286 | 10.7861 |
| 27 | - | 10 | 47.93267 | 10.9155 | 47.93267 | 10.7861 |
| 27 | - | 40 | 36.04308 | 10.9155 | 36.04308 | 10.7861 |
| 27 | - | 90 | 14.66772 | 10.9155 | 14.66772 | 10.7861 |
| 27 | + | 0 | 42.74331 | 10.9155 | 42.74331 | 10.7861 |
| 27 | + | 10 | 40.02209 | 10.9155 | 40.02209 | 10.7861 |
| 27 | + | 40 | 30.60120 | 10.9155 | 30.60120 | 10.7861 |
| 27 | + | 90 | 32.34847 | 10.9155 | 32.34847 | 10.7861 |
| 33 | - | 0 | 112.01847 | 15.7225 | 112.01847 | 15.5361 |
| 33 | - | 10 | 106.49127 | 15.7225 | 106.49127 | 15.5361 |
| 33 | - | 40 | 84.11786 | 15.7225 | 84.11786 | 15.5361 |
| 33 | - | 90 | 29.12448 | 15.7225 | 29.12448 | 15.5361 |
| 33 | + | 0 | 81.33478 | 15.7225 | 81.33478 | 15.5361 |
| 33 | + | 10 | 84.19125 | 15.7225 | 84.19125 | 15.5361 |
| 33 | + | 40 | 66.38026 | 15.7225 | 66.38026 | 15.5361 |
| 33 | + | 90 | 70.00257 | 15.7225 | 70.00257 | 15.5361 |
| 39 | - | 0 | 146.14435 | 15.0862 | 146.14435 | 14.9073 |
| 39 | - | 10 | 142.03229 | 15.0862 | 142.03229 | 14.9073 |
| 39 | - | 40 | 118.62116 | 15.0862 | 118.62116 | 14.9073 |
| 39 | - | 90 | 45.60211 | 15.0862 | 45.60211 | 14.9073 |
| 39 | + | 0 | 106.31225 | 15.0862 | 106.31225 | 14.9073 |
| 39 | + | 10 | 116.49478 | 15.0862 | 116.49478 | 14.9073 |
| 39 | + | 40 | 96.35160 | 15.0862 | 96.35160 | 14.9073 |
| 39 | + | 90 | 98.27789 | 15.0862 | 98.27789 | 14.9073 |
| 43 | - | 0 | 150.83970 | 14.9343 | 150.83970 | 14.7572 |
| 43 | - | 10 | 145.86938 | 14.9343 | 145.86938 | 14.7572 |
| 43 | - | 40 | 130.15988 | 14.9343 | 130.15988 | 14.7572 |
| 43 | - | 90 | 56.72693 | 14.9343 | 56.72693 | 14.7572 |
| 43 | + | 0 | 116.06750 | 14.9343 | 116.06750 | 14.7572 |
| 43 | + | 10 | 128.64563 | 14.9343 | 128.64563 | 14.7572 |
| 43 | + | 40 | 108.74468 | 14.9343 | 108.74468 | 14.7572 |
| 43 | + | 90 | 109.77001 | 14.9343 | 109.77001 | 14.7572 |
| 51 | - | 0 | 151.44971 | 28.0834 | 151.44971 | 27.7505 |
| 51 | - | 10 | 141.86055 | 28.0834 | 141.86055 | 27.7505 |
| 51 | - | 40 | 151.35577 | 28.0834 | 151.35577 | 27.7505 |
| 51 | - | 90 | 78.34587 | 28.0834 | 78.34587 | 27.7505 |
| 51 | + | 0 | 137.51969 | 28.0834 | 137.51969 | 27.7505 |
| 51 | + | 10 | 149.93282 | 28.0834 | 149.93282 | 27.7505 |
| 51 | + | 40 | 128.14947 | 28.0834 | 128.14947 | 27.7505 |
| 51 | + | 90 | 132.76307 | 28.0834 | 132.76307 | 27.7505 |

**Table S8: Comparison of predictions and mean LSDs for sPSA AGR**

| | | | Separate analyses | | Joint analyses | |
|---|---|---|---|---|---|---|
| DAP | AMF | Zn | Prediction | LSD (5%) | Prediction | LSD (5%) |
| 18–22 | - | 0 | 2.59610 | 0.6714 | 2.59610 | 0.6622 |
| 18–22 | - | 10 | 2.34767 | 0.6714 | 2.34767 | 0.6622 |
| 18–22 | - | 40 | 1.72428 | 0.6714 | 1.72428 | 0.6622 |
| 18–22 | - | 90 | 0.77123 | 0.6714 | 0.77123 | 0.6622 |
| 18–22 | + | 0 | 2.32242 | 0.6714 | 2.32242 | 0.6622 |
| 18–22 | + | 10 | 2.09478 | 0.6714 | 2.09478 | 0.6622 |
| 18–22 | + | 40 | 1.55713 | 0.6714 | 1.55713 | 0.6622 |
| 18–22 | + | 90 | 1.69610 | 0.6714 | 1.69610 | 0.6622 |
| 22–27 | - | 0 | 6.67500 | 1.1459 | 6.67500 | 1.1303 |
| 22–27 | - | 10 | 6.26092 | 1.1459 | 6.26092 | 1.1303 |
| 22–27 | - | 40 | 4.62114 | 1.1459 | 4.62114 | 1.1303 |
| 22–27 | - | 90 | 1.49104 | 1.1459 | 1.49104 | 1.1303 |
| 22–27 | + | 0 | 4.87395 | 1.1459 | 4.87395 | 1.1303 |
| 22–27 | + | 10 | 4.73998 | 1.1459 | 4.73998 | 1.1303 |
| 22–27 | + | 40 | 3.69805 | 1.1459 | 3.69805 | 1.1303 |
| 22–27 | + | 90 | 4.09366 | 1.1459 | 4.09366 | 1.1303 |
| 27–33 | - | 0 | 10.12927 | 1.1011 | 10.12927 | 1.0860 |
| 27–33 | - | 10 | 9.75977 | 1.1011 | 9.75977 | 1.0860 |
| 27–33 | - | 40 | 8.01246 | 1.1011 | 8.01246 | 1.0860 |
| 27–33 | - | 90 | 2.40946 | 1.1011 | 2.40946 | 1.0860 |
| 27–33 | + | 0 | 6.43191 | 1.1011 | 6.43191 | 1.0860 |
| 27–33 | + | 10 | 7.36153 | 1.1011 | 7.36153 | 1.0860 |
| 27–33 | + | 40 | 5.96318 | 1.1011 | 5.96318 | 1.0860 |
| 27–33 | + | 90 | 6.27568 | 1.1011 | 6.27568 | 1.0860 |
| 33–39 | - | 0 | 5.68765 | 0.8368 | 5.68765 | 0.8254 |
| 33–39 | - | 10 | 5.92350 | 0.8368 | 5.92350 | 0.8254 |
| 33–39 | - | 40 | 5.75055 | 0.8368 | 5.75055 | 0.8254 |
| 33–39 | - | 90 | 2.74627 | 0.8368 | 2.74627 | 0.8254 |
| 33–39 | + | 0 | 4.16291 | 0.8368 | 4.16291 | 0.8254 |
| 33–39 | + | 10 | 5.38392 | 0.8368 | 5.38392 | 0.8254 |
| 33–39 | + | 40 | 4.99522 | 0.8368 | 4.99522 | 0.8254 |
| 33–39 | + | 90 | 4.71255 | 0.8368 | 4.71255 | 0.8254 |
| 39–43 | - | 0 | 1.17384 | 1.6814 | 1.17384 | 1.6585 |
| 39–43 | - | 10 | 0.95927 | 1.6814 | 0.95927 | 1.6585 |
| 39–43 | - | 40 | 2.88468 | 1.6814 | 2.88468 | 1.6585 |
| 39–43 | - | 90 | 2.78121 | 1.6814 | 2.78121 | 1.6585 |
| 39–43 | + | 0 | 2.43881 | 1.6814 | 2.43881 | 1.6585 |
| 39–43 | + | 10 | 3.03771 | 1.6814 | 3.03771 | 1.6585 |
| 39–43 | + | 40 | 3.09827 | 1.6814 | 3.09827 | 1.6585 |
| 39–43 | + | 90 | 2.87303 | 1.6814 | 2.87303 | 1.6585 |
| 43–51 | - | 0 | 0.07625 | 2.5153 | 0.07625 | 2.4809 |
| 43–51 | - | 10 | -0.50110 | 2.5153 | -0.50110 | 2.4809 |
| 43–51 | - | 40 | 2.64949 | 2.5153 | 2.64949 | 2.4809 |
| 43–51 | - | 90 | 2.70237 | 2.5153 | 2.70237 | 2.4809 |
| 43–51 | + | 0 | 2.68152 | 2.5153 | 2.68152 | 2.4809 |
| 43–51 | + | 10 | 2.66090 | 2.5153 | 2.66090 | 2.4809 |
| 43–51 | + | 40 | 2.42560 | 2.5153 | 2.42560 | 2.4809 |
| 43–51 | + | 90 | 2.87413 | 2.5153 | 2.87413 | 2.4809 |

**Table S9: Comparison of predictions and mean LSDs for sPSA RGR**

| | | | Separate analyses | | Joint analyses | |
|---|---|---|---|---|---|---|
| DAP | AMF | Zn | Prediction | LSD (5%) | Prediction | LSD (5%) |
| 18–22 | - | 0 | 0.22677 | 0.0456 | 0.22677 | 0.0450 |
| 18–22 | - | 10 | 0.21103 | 0.0456 | 0.21103 | 0.0450 |
| 18–22 | - | 40 | 0.19333 | 0.0456 | 0.19333 | 0.0450 |
| 18–22 | - | 90 | 0.14470 | 0.0456 | 0.14470 | 0.0450 |
| 18–22 | + | 0 | 0.17960 | 0.0456 | 0.17960 | 0.0450 |
| 18–22 | + | 10 | 0.17964 | 0.0456 | 0.17964 | 0.0450 |
| 18–22 | + | 40 | 0.19441 | 0.0456 | 0.19441 | 0.0450 |
| 18–22 | + | 90 | 0.20741 | 0.0456 | 0.20741 | 0.0450 |
| 22–27 | - | 0 | 0.21272 | 0.0288 | 0.21272 | 0.0284 |
| 22–27 | - | 10 | 0.21255 | 0.0288 | 0.21255 | 0.0284 |
| 22–27 | - | 40 | 0.20573 | 0.0288 | 0.20573 | 0.0284 |
| 22–27 | - | 90 | 0.14503 | 0.0288 | 0.14503 | 0.0284 |
| 22–27 | + | 0 | 0.17307 | 0.0288 | 0.17307 | 0.0284 |
| 22–27 | + | 10 | 0.17897 | 0.0288 | 0.17897 | 0.0284 |
| 22–27 | + | 40 | 0.18944 | 0.0288 | 0.18944 | 0.0284 |
| 22–27 | + | 90 | 0.19927 | 0.0288 | 0.19927 | 0.0284 |
| 27–33 | - | 0 | 0.13055 | 0.0227 | 0.13055 | 0.0224 |
| 27–33 | - | 10 | 0.13338 | 0.0227 | 0.13338 | 0.0224 |
| 27–33 | - | 40 | 0.14161 | 0.0227 | 0.14161 | 0.0224 |
| 27–33 | - | 90 | 0.11843 | 0.0227 | 0.11843 | 0.0224 |
| 27–33 | + | 0 | 0.11069 | 0.0227 | 0.11069 | 0.0224 |
| 27–33 | + | 10 | 0.12463 | 0.0227 | 0.12463 | 0.0224 |
| 27–33 | + | 40 | 0.12954 | 0.0227 | 0.12954 | 0.0224 |
| 27–33 | + | 90 | 0.12956 | 0.0227 | 0.12956 | 0.0224 |
| 33–39 | - | 0 | 0.04449 | 0.0170 | 0.04449 | 0.0167 |
| 33–39 | - | 10 | 0.04862 | 0.0170 | 0.04862 | 0.0167 |
| 33–39 | - | 40 | 0.05724 | 0.0170 | 0.05724 | 0.0167 |
| 33–39 | - | 90 | 0.07863 | 0.0170 | 0.07863 | 0.0167 |
| 33–39 | + | 0 | 0.04619 | 0.0170 | 0.04619 | 0.0167 |
| 33–39 | + | 10 | 0.05471 | 0.0170 | 0.05471 | 0.0167 |
| 33–39 | + | 40 | 0.06226 | 0.0170 | 0.06226 | 0.0167 |
| 33–39 | + | 90 | 0.05742 | 0.0170 | 0.05742 | 0.0167 |
| 39–43 | - | 0 | 0.00774 | 0.0160 | 0.00774 | 0.0158 |
| 39–43 | - | 10 | 0.00658 | 0.0160 | 0.00658 | 0.0158 |
| 39–43 | - | 40 | 0.02298 | 0.0160 | 0.02298 | 0.0158 |
| 39–43 | - | 90 | 0.05721 | 0.0160 | 0.05721 | 0.0158 |
| 39–43 | + | 0 | 0.02293 | 0.0160 | 0.02293 | 0.0158 |
| 39–43 | + | 10 | 0.02500 | 0.0160 | 0.02500 | 0.0158 |
| 39–43 | + | 40 | 0.03022 | 0.0160 | 0.03022 | 0.0158 |
| 39–43 | + | 90 | 0.02827 | 0.0160 | 0.02827 | 0.0158 |
| 43–51 | - | 0 | -0.00026 | 0.0204 | -0.00026 | 0.0202 |
| 43–51 | - | 10 | -0.00610 | 0.0204 | -0.00610 | 0.0202 |
| 43–51 | - | 40 | 0.01803 | 0.0204 | 0.01803 | 0.0202 |
| 43–51 | - | 90 | 0.04243 | 0.0204 | 0.04243 | 0.0202 |
| 43–51 | + | 0 | 0.02148 | 0.0204 | 0.02148 | 0.0202 |
| 43–51 | + | 10 | 0.01901 | 0.0204 | 0.01901 | 0.0202 |
| 43–51 | + | 40 | 0.02051 | 0.0204 | 0.02051 | 0.0202 |
| 43–51 | + | 90 | 0.02371 | 0.0204 | 0.02371 | 0.0202 |
