# Supplementary material for: Smoothing and extraction of traits in the growth analysis of noninvasive phenotypic data
Source: Plant Methods. 2020 Mar 10;16:36. doi: 10.1186/s13007-020-00577-6 (PMC7065360; doi:10.1186/s13007-020-00577-6)
Supplement: Supplementary file 3 — Additional file 3. Supporting material for the longitudinal analysis. Four plots comparing the predicted trends for four different knot numbers and four plots showing the deviations between unsmoothed and smoothed predictions. A table of Wald F-statistic p-values and a summary table of the variance model hypothesis testing for each of PSA and ln(PSA) for the full variance model. A table of Wald F-statistic p-values for each of PSA and ln(PSA) for the reduced variance model. Two plots comparing the predictions and standard errors obtained under the full and reduced variance models. Residual-versus-fitted-values, residual boxplots for different DAPS and normal probability plots for the longitudinal analysis of PSA and ln(PSA). [file 13007_2020_577_MOESM3_ESM.pdf]

Smoothing and extraction of traits in the growth analysis of noninvasive phenotypic data

by C. Brien, N. Jewell, S.J. Watt-Williams, T. Garnett and B. Berger.

https://dx.doi.org/10.1186/s13007-020-00577-6

Additional File 3: Supporting material for the longitudinal analyses

Plots comparing predictions obtained with differing numbers of knots

The plots in Additional Figures S1–S8 compare the smoothing achieved with 10, 15, 20, 35 knots for the PSA, backtransformed PSA (from the analysis of the ln(PSA)), PSA AGR and PSA RGR (from the predicted ln(PSA)).

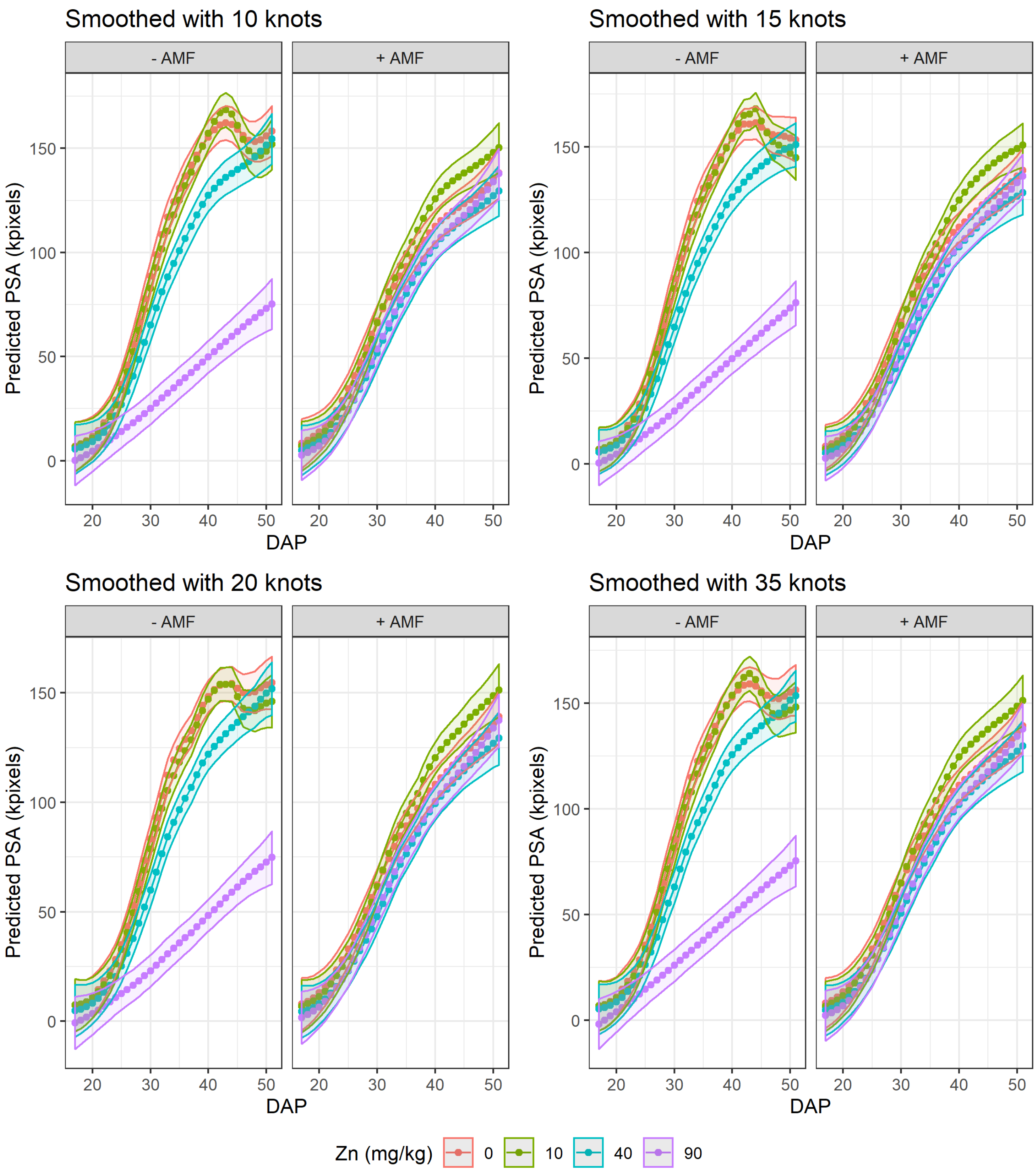

Figure S1: Plot of predicted PSA for combinations of Zn and AMF that compares the smoothing achieved with 10, 15, 20 and 35 knots. The error ribbons are the predicted values ± 0.5 LSD ($\alpha = 0.05$). Separated ribbons indicate a significant difference, in contrast to overlapping ribbons for which there is not a significant difference.

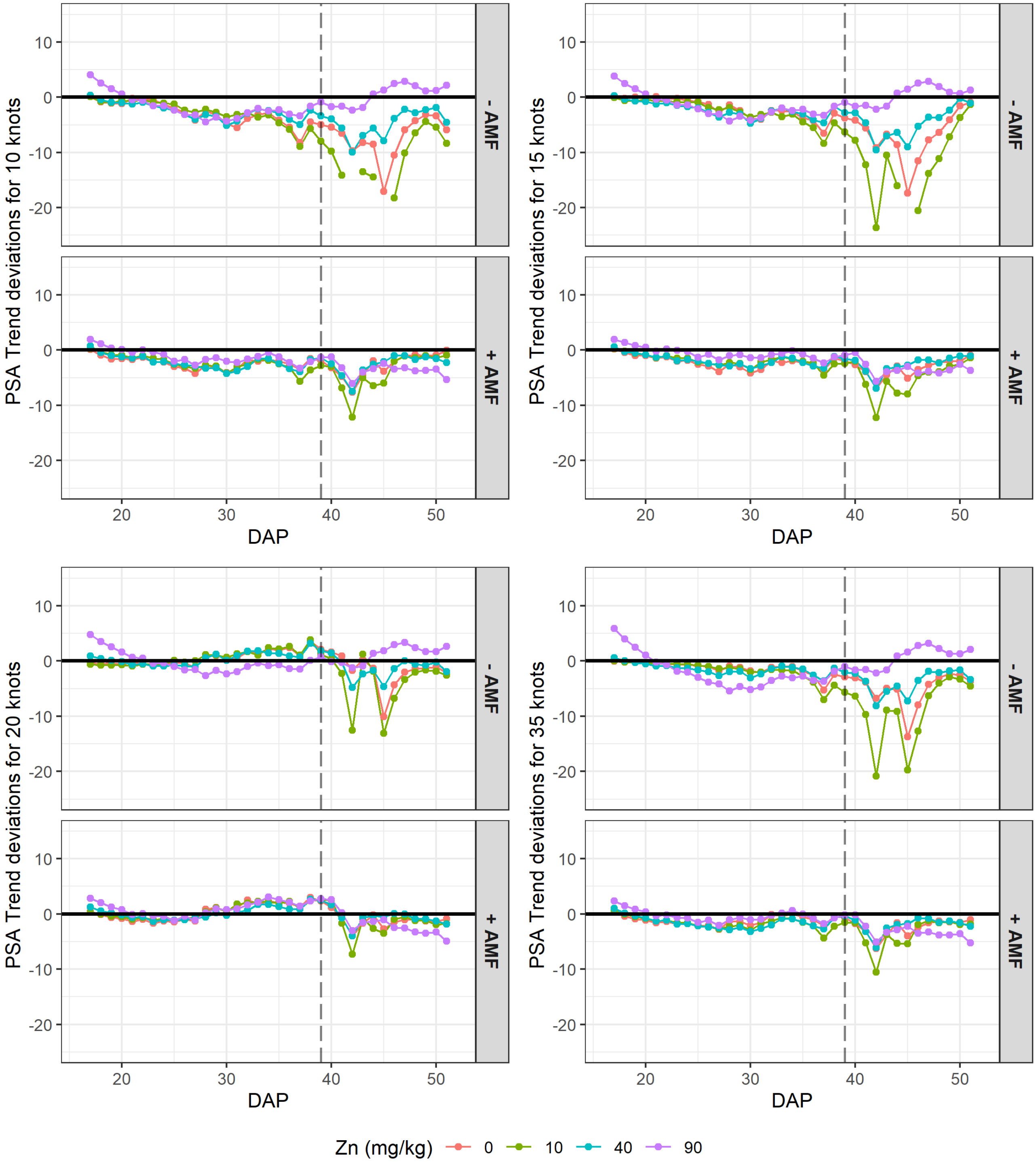

Figure S2: Plot of deviations between unsmoothed and smoothed predicted PSA for combinations of Zn and AMF that compares the smoothing achieved with 10, 15, 20 and 35 knots.

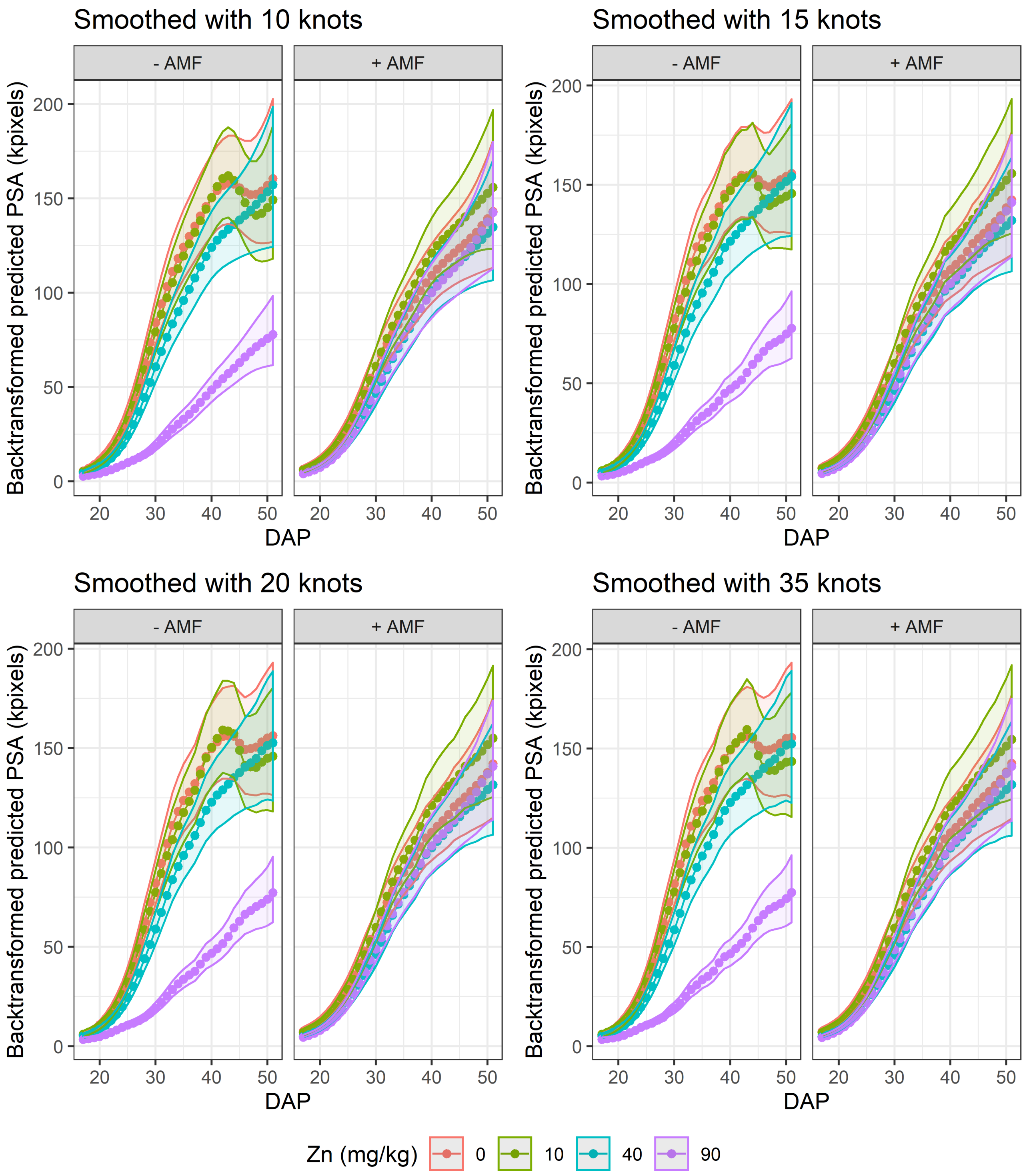

Figure S3: Plot of backtransformed predicted PSA for combinations of Zn and AMF that compares the smoothing achieved with 10, 15, 20 and 35 knots. The error ribbons are the predicted values ± 0.5 LSD ($\alpha = 0.05$). Separated ribbons indicate a significant difference, in contrast to overlapping ribbons for which there is not a significant difference.

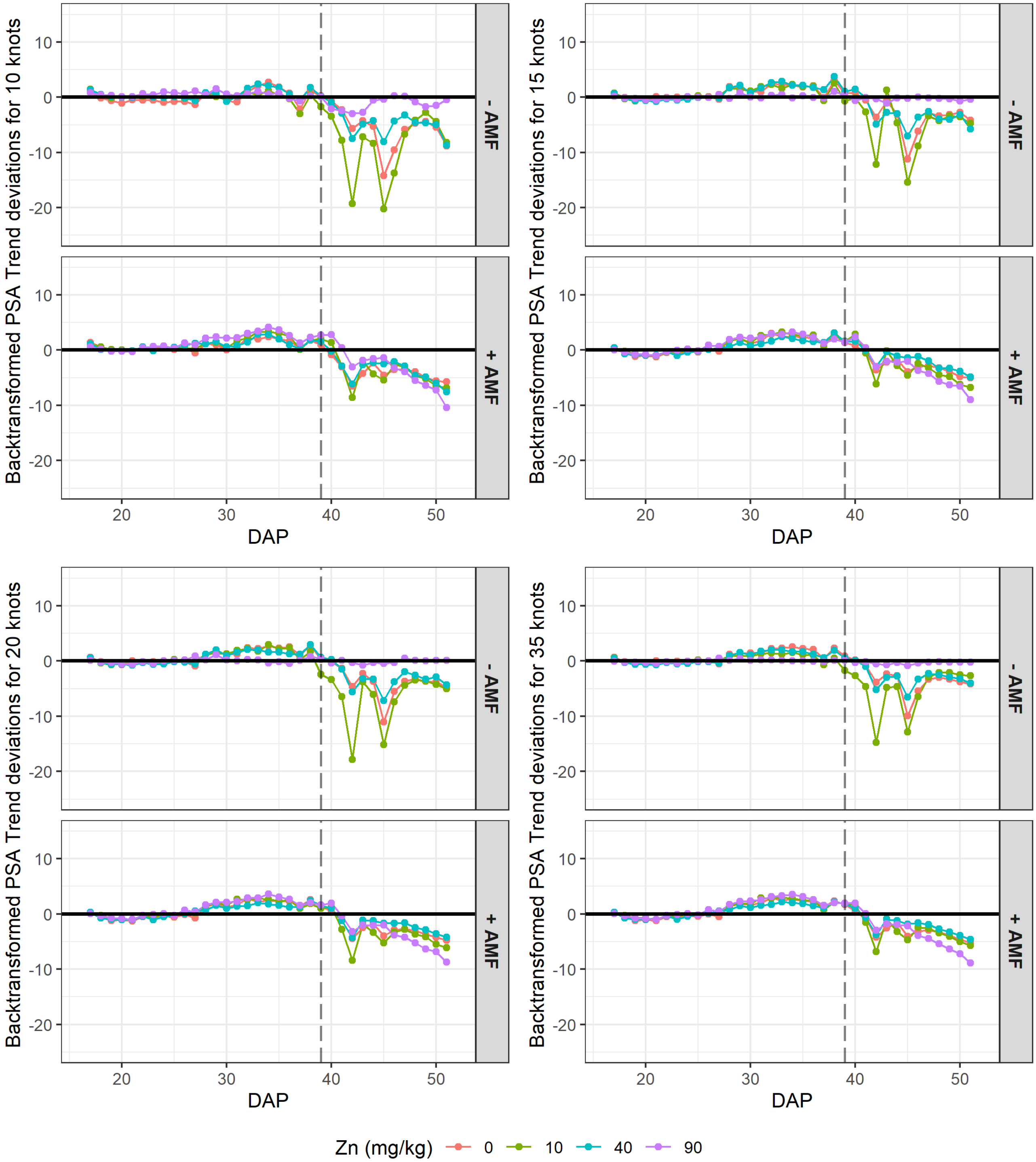

Figure S4: Plot of deviations between unsmoothed and smoothed backtransformed predicted PSA for combinations of Zn and AMF that compares the smoothing achieved with 10, 15, 20 and 35 knots.

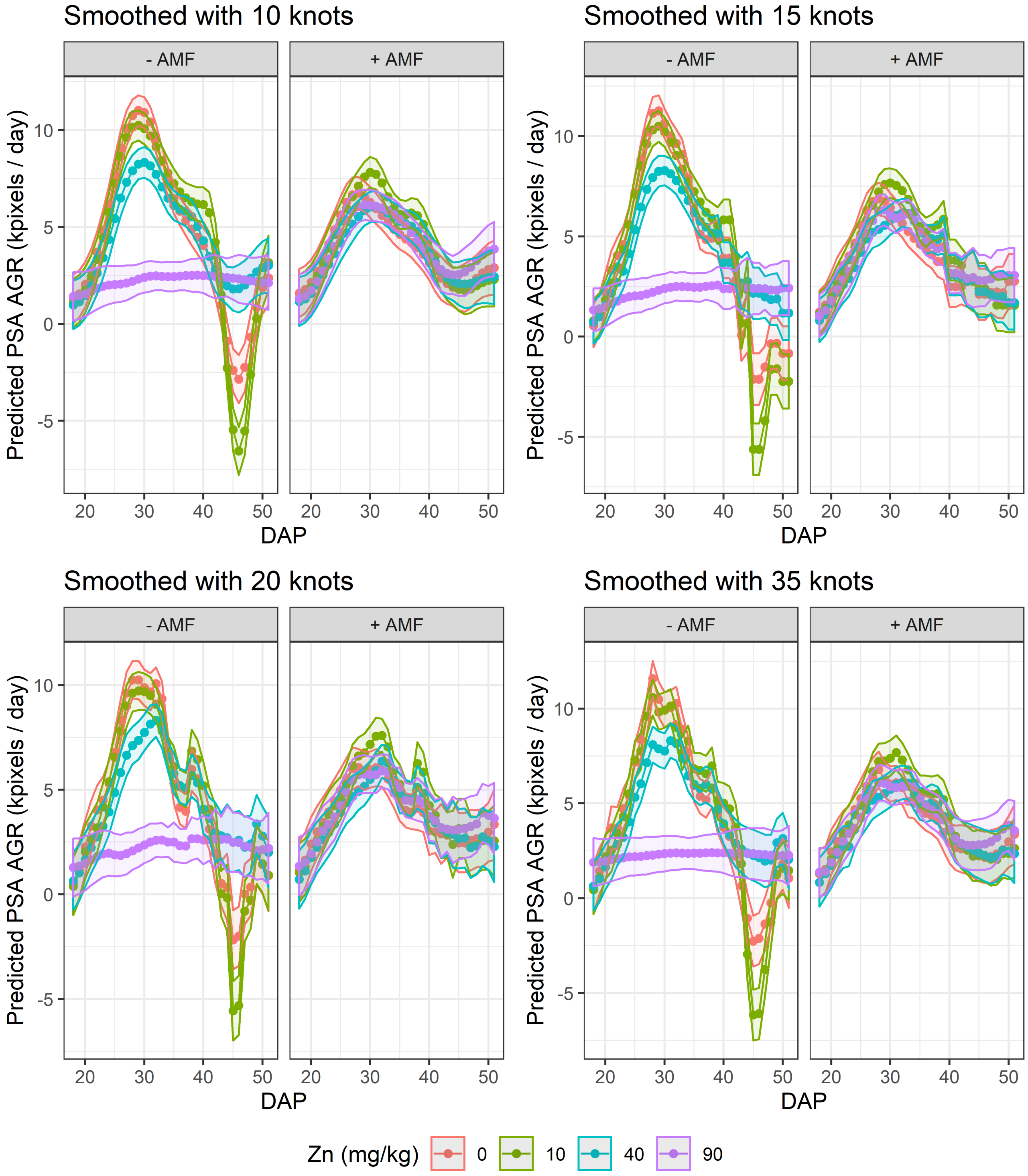

Figure S5: Plot of predicted PSA AGR for combinations of Zn and AMF that compares the smoothing achieved with 10, 15, 20 and 35 knots. The error ribbons are the predicted values ± 0.5 LSD ($\alpha = 0.05$). Separated ribbons indicate a significant difference, in contrast to overlapping ribbons for which there is not a significant difference.

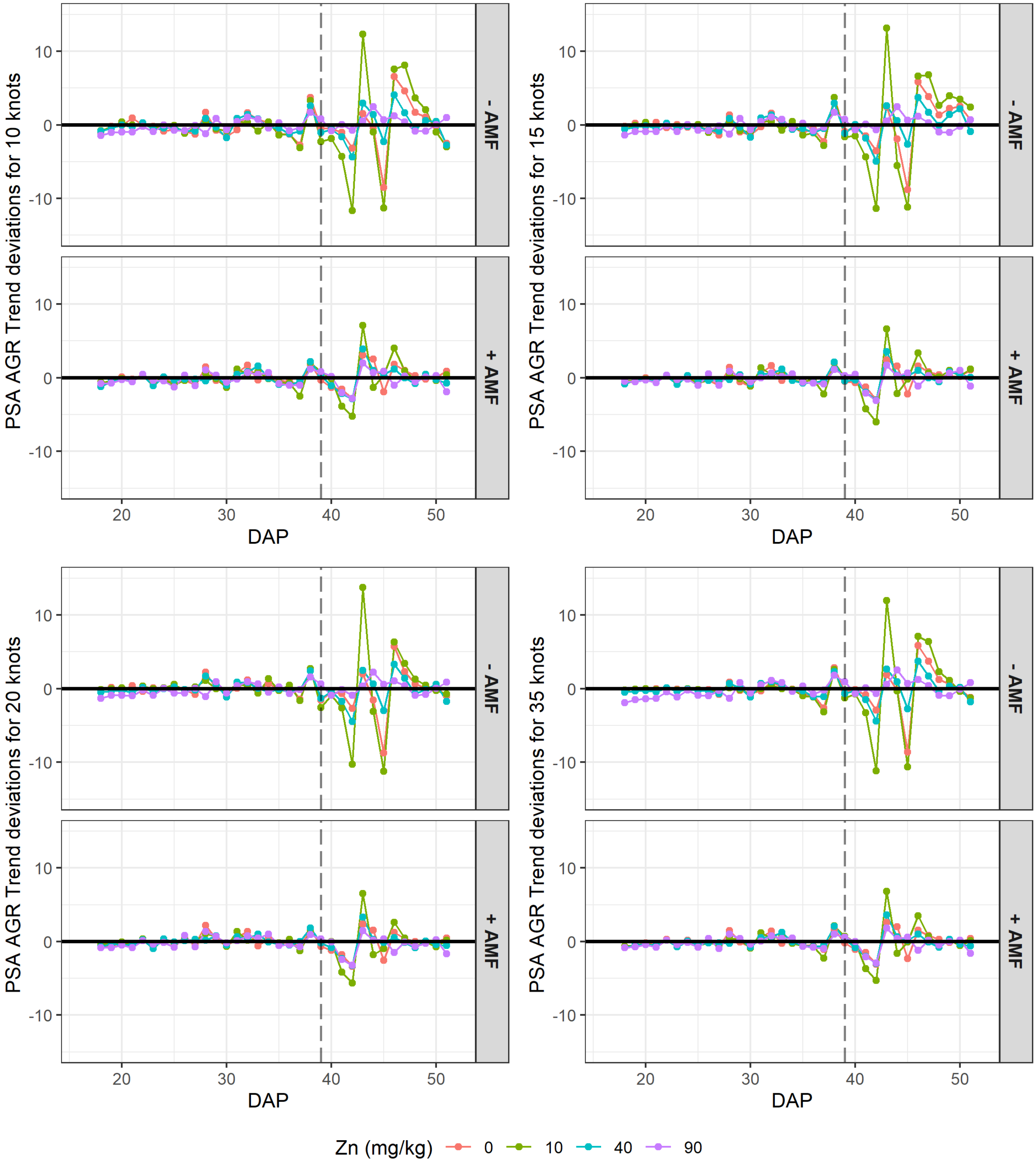

Figure S6: Plot of deviations between unsmoothed and smoothed predicted PSA AGR for combinations of Zn and AMF that compares the smoothing achieved with 10, 15, 20 and 35 knots.

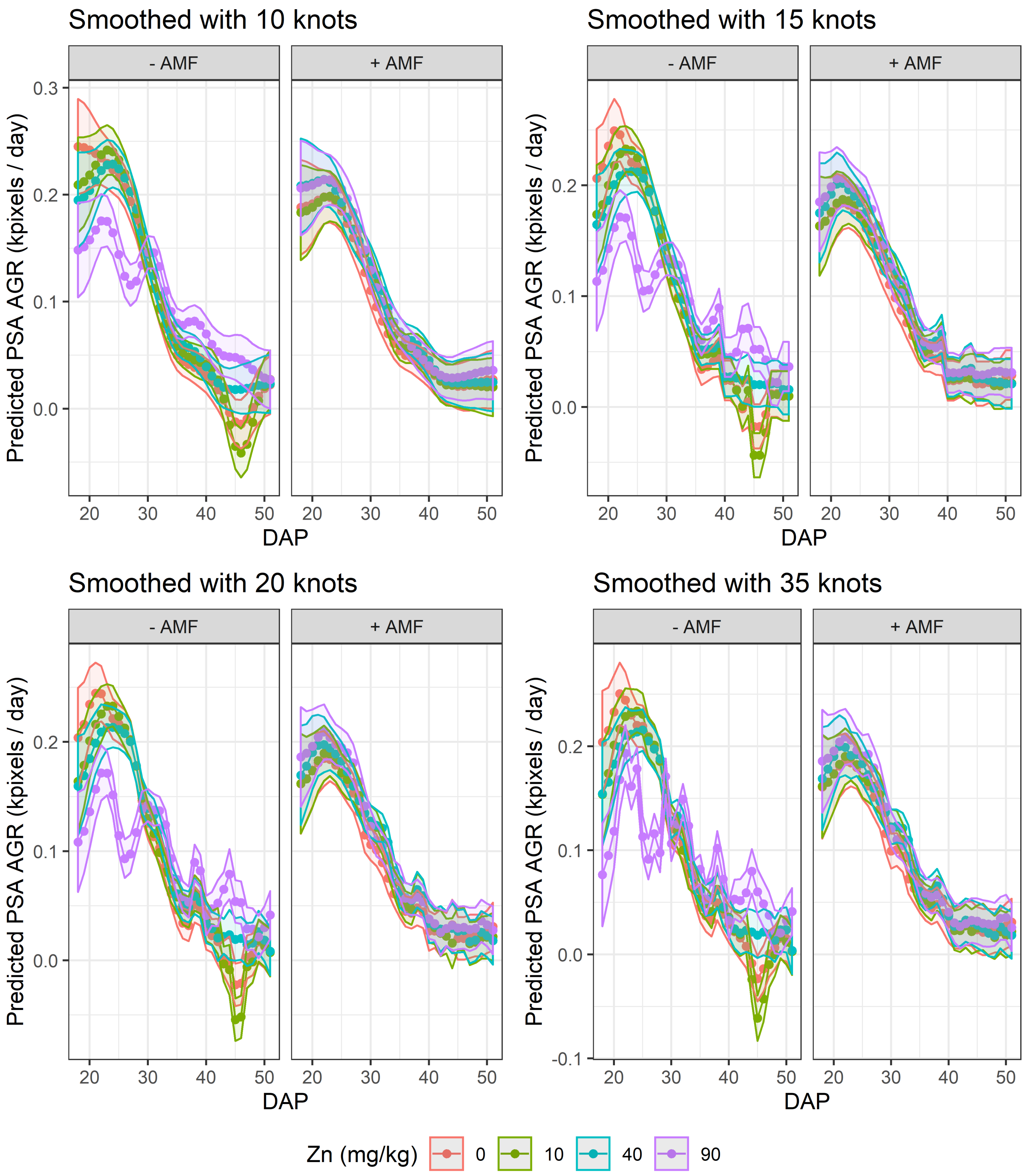

Figure S7: Plot of predicted PSA RGR for combinations of Zn and AMF that compares the smoothing achieved with 10, 15, 20 and 35 knots. The error ribbons are the predicted values ± 0.5 LSD ($\alpha = 0.05$). Separated ribbons indicate a significant difference, in contrast to overlapping ribbons for which there is not a significant difference.

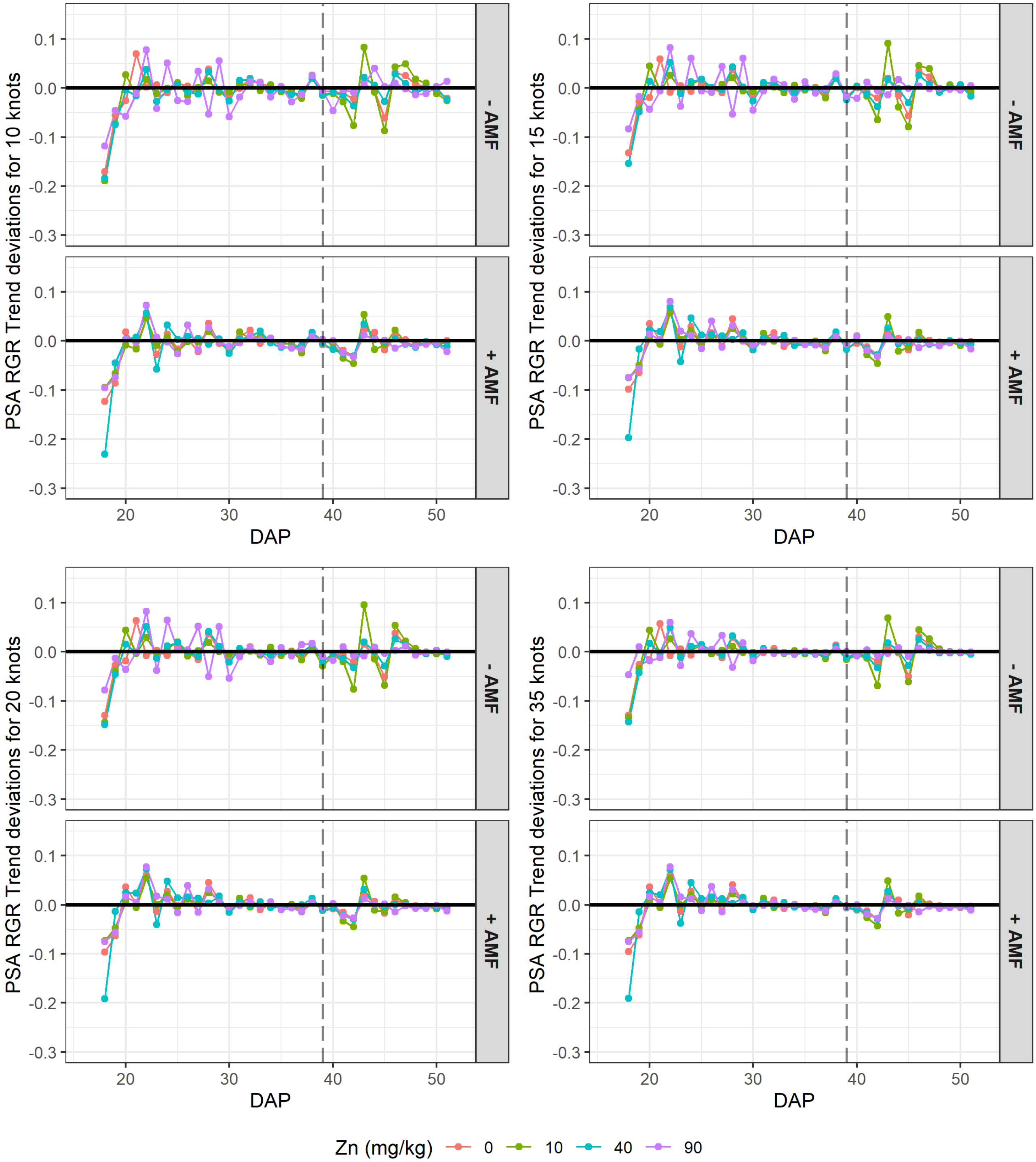

Figure S8: Plot of deviations between unsmoothed and smoothed predicted PSA RGR for combinations of Zn and AMF that compares the smoothing achieved with 10, 15, 20 and 35 knots.

Summary of hypothesis testing for the full variance model

Table S1: The p-values of the Wald F-statistics for PSA

Term	DF	Denominator DF	Wald F	p
(Intercept)	1	21.2	3027.00	0.000
Block	3	20.0	4.47	0.015
Treatments	7	21.2	25.03	0.000
Treatments:xDAP	8	23.8	229.40	0.000

Table S2: The p-values of the tests for the variance model for PSA

Term/change	DF	p	Action
Remove bound intercept-slope covariance			
DAP heterogeneity	35	0.000	
DAP autocorrelation	1	0.000	
Change to hetergeneous splines for Treatments:DAP			
Add random deviations			
Treatments:DAP	1	0.080	
Heterogeneous Treatment splines	6	0.001	

Table S3: The p-values of the Wald F-statistics for ln(PSA)

Term	DF	Denominator DF	Wald F	p
(Intercept)	1	21.8	16990.00	0.000
Block	3	20.0	2.75	0.069
AMF	1	20.2	1.40	0.250
Zn	3	20.0	27.78	0.000
xDAP	1	25.0	1995.00	0.000
AMF:Zn	3	20.0	12.14	0.000
AMF:xDAP	1	23.6	0.43	0.518
Zn:xDAP	3	23.0	1.53	0.233
AMF:Zn:xDAP	3	23.0	0.26	0.854

Summary of hypothesis testing for the reduced variance model

Comparison of Additional Tables S5 and S6 with Additional Tables S1 and S3 will reveal that the p-values differ between the reduced and full models.

Table S4: The p-values of the tests for the variance model for ln(PSA)

Term/change	DF	p	Action
Intercept-slope covariance	1	1.000	
DAP heterogeneity	38	0.000	
DAP autocorrelation	1	0.000	
Change to hetergeneous splines for Treatments:DAP			
Add random deviations			
Treatments:DAP	1	0.108	
Heterogeneous Treatment splines	6	0.990	
Heterogeneous AMF splines	1	0.123	
Heterogeneous Zn splines	3	0.866	
Change to AMF*ZN*xDAP splines			
spl(xDAP, k = 10):Zn	1		
spl(xDAP, k = 10):AMF	1		
Add random deviations			
AMF:DAP:Zn	1		
AMF:Zn:DAP			
spl(xDAP, k = 10):AMF:Zn	1	0.002	

Table S5: The p-values of the Wald F-statistics for PSA

Term	DF	Denominator DF	Wald F	p
(Intercept)	1	20.3	2557.00	0.000
Block	3	20.0	3.47	0.035
Treatments	7	20.3	21.62	0.000
Treatments:xDAP	8	23.3	204.40	0.000

Table S6: The p-values of the Wald F-statistics for ln(PSA)

Term	DF	Denominator DF	Wald F	p
(Intercept)	1	21.5	18250.00	0.000
Block	3	20.0	2.92	0.059
AMF	1	20.8	1.53	0.229
Zn	3	20.3	30.07	0.000
xDAP	1	27.1	1903.00	0.000
AMF:Zn	3	20.3	12.99	0.000
AMF:xDAP	1	24.6	0.46	0.502
Zn:xDAP	3	23.6	1.88	0.161
AMF:Zn:xDAP	3	23.6	0.08	0.969

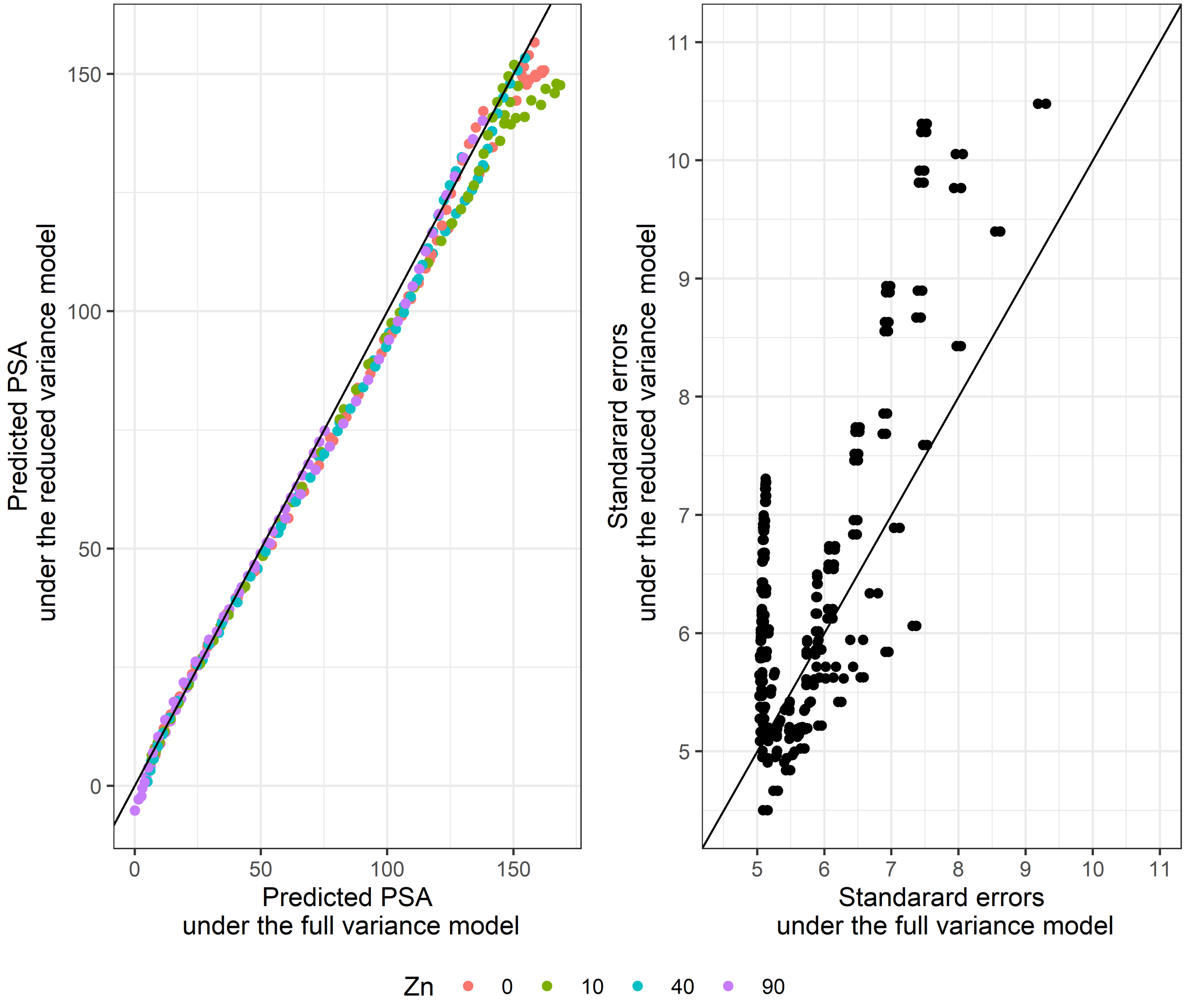

Figure S9: Comparison of predictions and standard errors under full and reduced models for PSA.

Plots comparing predictions and standard errors under full and reduced models for PSA

Additional Figure S9 shows the predicted values for and their standard errors for two variance models: (i) a full model with unequal variances and first-order autocorrelations between times (x-axis), and (ii) a reduced model with equal variances and no correlation between times (y-axis). Clearly the models lead to different predictions and standard errors. Indeed the full model generally has lower standard errors.

Residual-versus-fitted-values, residual boxplots and normal probability plots of the residuals

The plots in Figures S10–S15 are based on the standardized conditional residuals from the analyses that investigate the effect of Zn and AMF on the trend over the DAPs (Stage 4). For the residual-versus-fitted-values plots, the expected pattern is one of a similar vertical distribution of points across all values plotted on the x-axis. Points outside the maroon lines are potential outliers. For normal probability plots, it can be concluded that the data are consistent with being normally distributed if all points lie within the envelopes included in the plot.

```
Spline: design points closer than 0.0034 have been merged.
Spline: design points closer than 0.0034 have been merged.
Spline: design points closer than 0.0034 have been merged.
Spline: design points closer than 0.0034 have been merged.
```

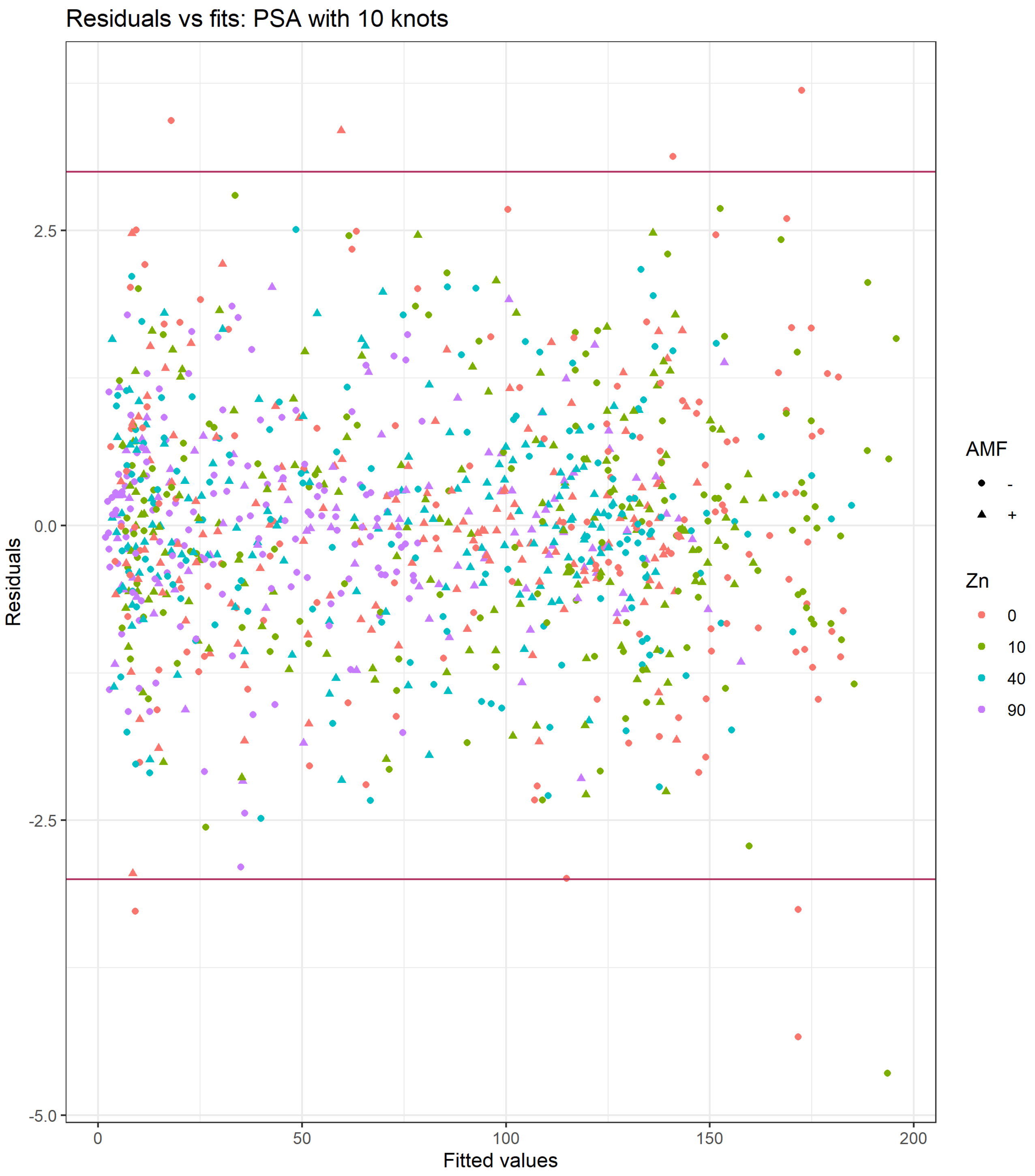

Figure S10: Residual plots for a trait.

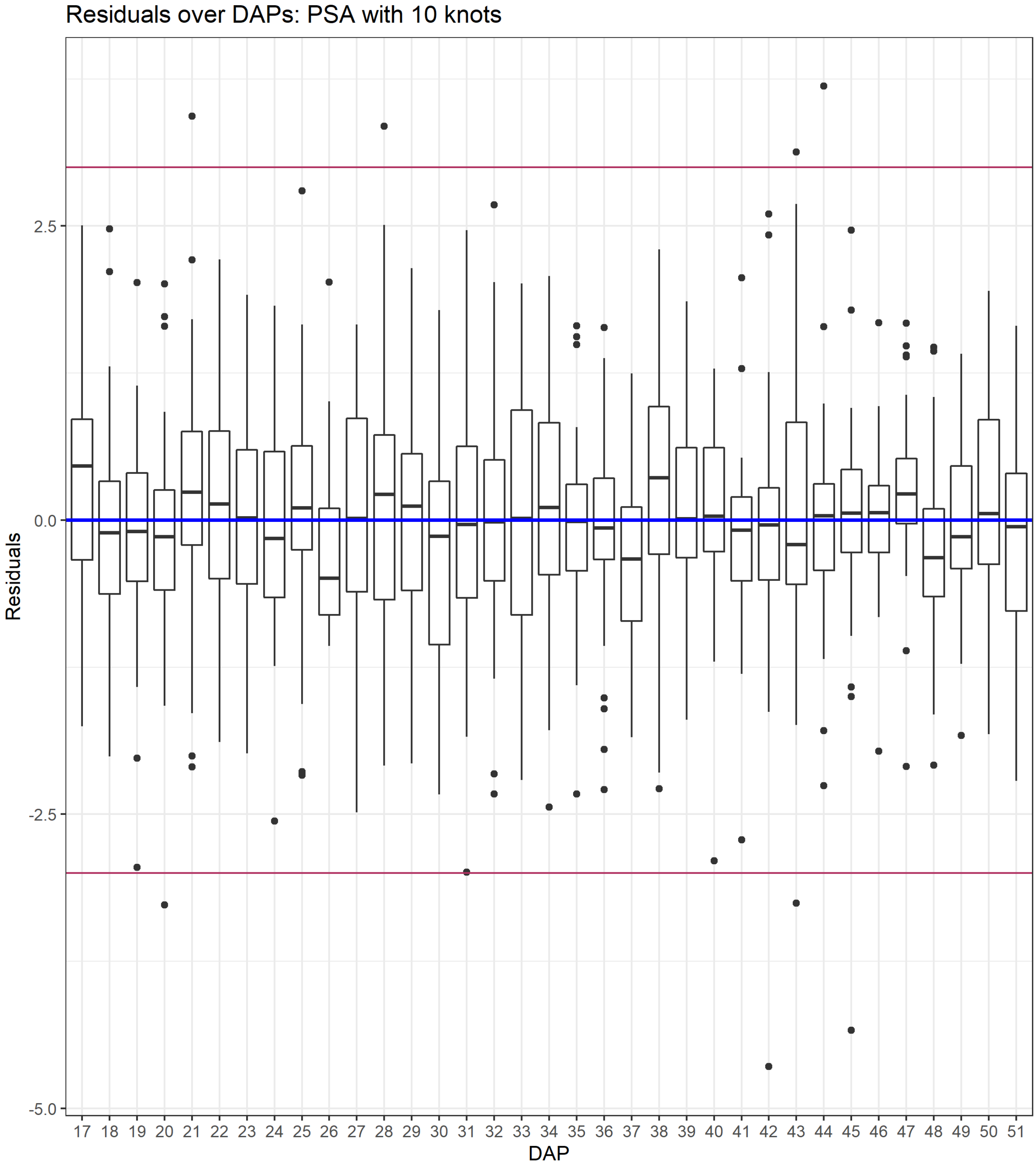

Figure S11: Residual plots for a trait.

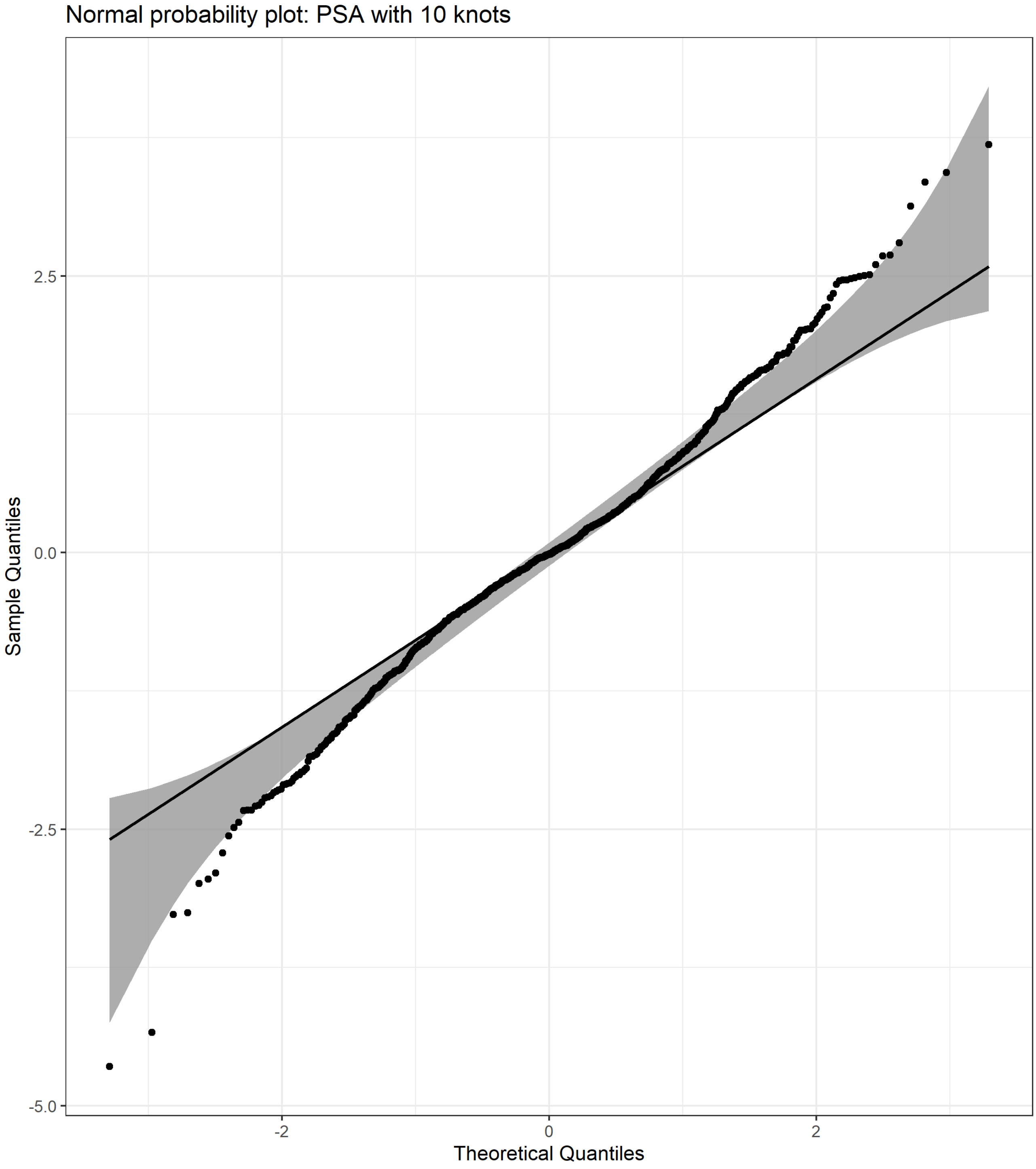

Figure S12: Residual plots for a trait.

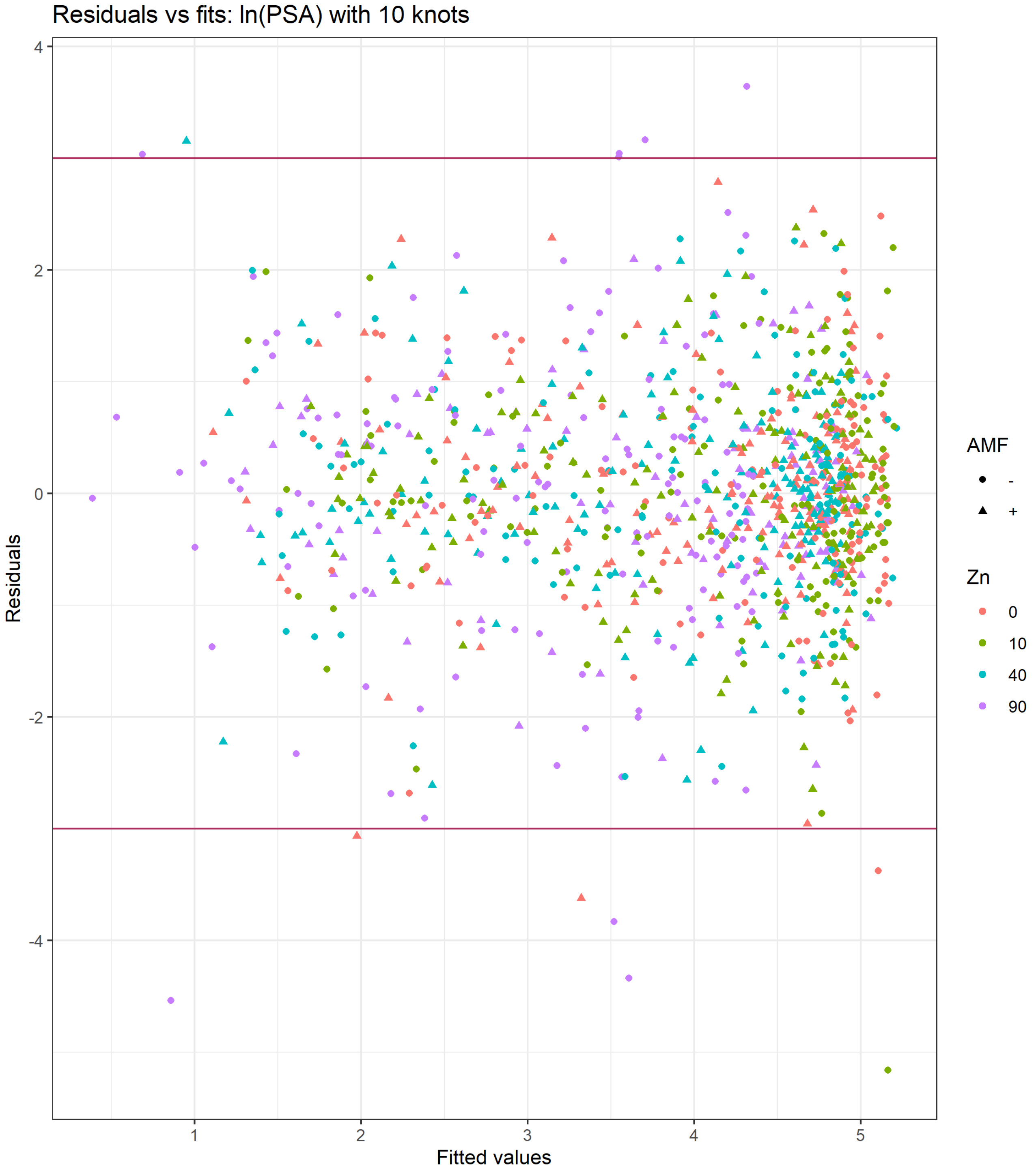

Figure S13: Residual plots for a trait.

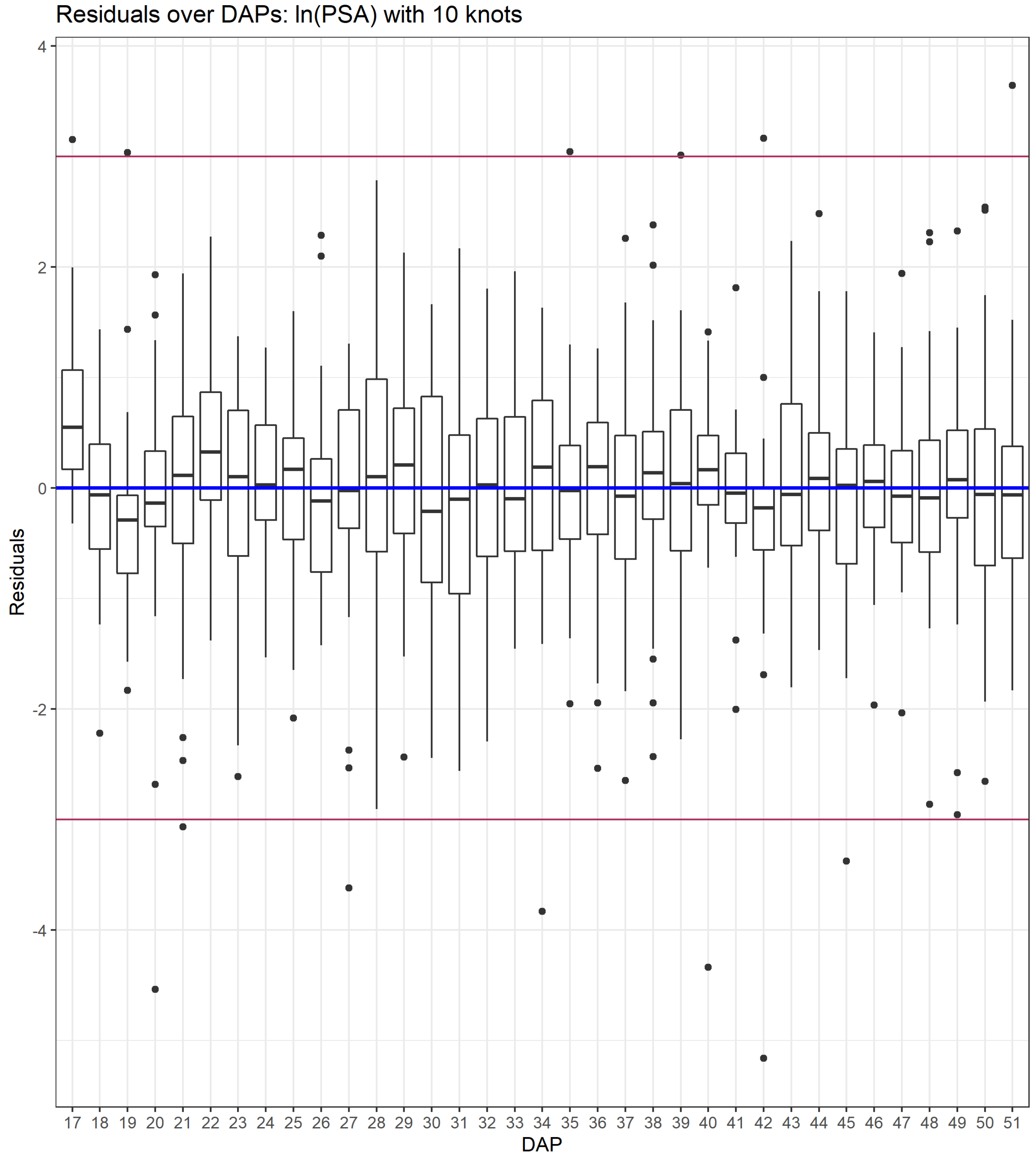

Figure S14: Residual plots for a trait.

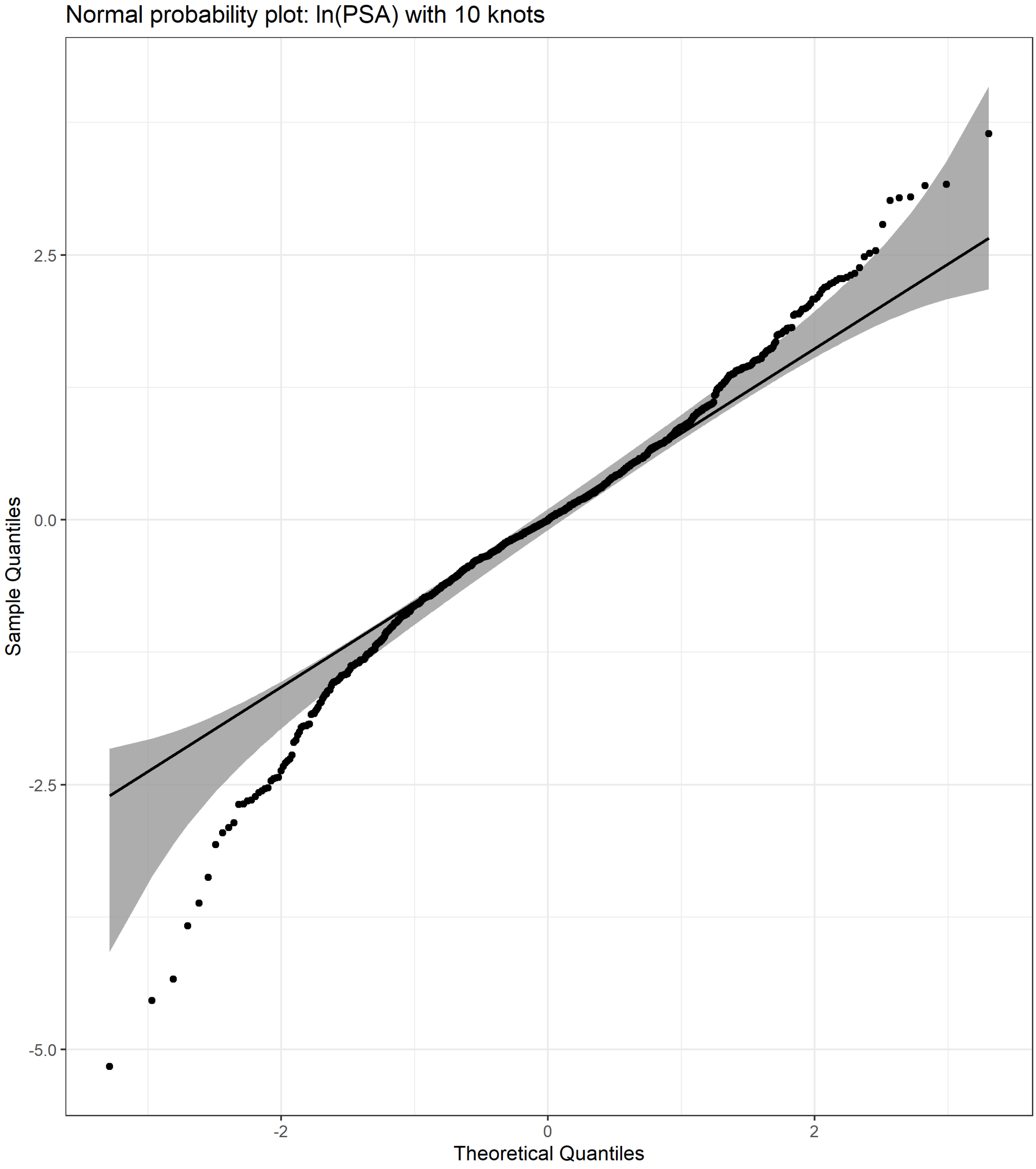

Figure S15: Residual plots for a trait.
